# Combinatorial *Cis*-regulation in *Saccharomyces* Species

**DOI:** 10.1534/g3.115.024331

**Published:** 2016-01-12

**Authors:** Aaron T. Spivak, Gary D. Stormo

**Affiliations:** Department of Genetics, Center for Genome Sciences and Systems Biology, Washington University School of Medicine, St Louis, Missouri 63108

**Keywords:** gene regulation, transcription factors, combinatorial regulation, transcriptional control

## Abstract

Transcriptional control of gene expression requires interactions between the *cis*-regulatory elements (CREs) controlling gene promoters. We developed a sensitive computational method to identify CRE combinations with conserved spacing that does not require genome alignments. When applied to seven *sensu stricto* and *sensu lato Saccharomyces* species, 80% of the predicted interactions displayed some evidence of combinatorial transcriptional behavior in several existing datasets including: (1) chromatin immunoprecipitation data for colocalization of transcription factors, (2) gene expression data for coexpression of predicted regulatory targets, and (3) gene ontology databases for common pathway membership of predicted regulatory targets. We tested several predicted CRE interactions with chromatin immunoprecipitation experiments in a wild-type strain and strains in which a predicted cofactor was deleted. Our experiments confirmed that transcription factor (TF) occupancy at the promoters of the CRE combination target genes depends on the predicted cofactor while occupancy of other promoters is independent of the predicted cofactor. Our method has the additional advantage of identifying regulatory differences between species. By analyzing the *S. cerevisiae* and *S. bayanus* genomes, we identified differences in combinatorial *cis*-regulation between the species and showed that the predicted changes in gene regulation explain several of the species-specific differences seen in gene expression datasets. In some instances, the same CRE combinations appear to regulate genes involved in distinct biological processes in the two different species. The results of this research demonstrate that (1) combinatorial *cis*-regulation can be inferred by multi-genome analysis and (2) combinatorial *cis*-regulation can explain differences in gene expression between species.

The combination of *cis*-regulatory elements (CREs) in a promoter is an important determinant of gene expression patterns (Pilpel *et al.* 2001; Balaji *et al.* 2006; Gertz and Cohen 2009; Kazemian *et al.* 2013; Nandi *et al.* 2013; Wang *et al.* 2013), but we have only a limited understanding of how interactions between regulatory elements affect gene expression. There is clear evidence that certain combinations of CREs produce nonadditive effects on gene expression (Pramila *et al.* 2002), but it remains very challenging to discover which CREs interact on a genome scale (Balaji *et al.* 2006; Aguilar and Oliva 2008; He *et al.* 2009; Girgis and Ovcharenko 2012; Ha *et al.* 2012; Kazemian *et al.* 2013; Nandi *et al.* 2013; Jiang and Singh 2014). Understanding eukaryotic gene expression requires identifying the CRE combinations that interact to produce nonadditive effects on gene expression.

Gene regulation studies using synthetic promoters made from random combinations of CREs have been successful in discovering new synergistic combinations (Gertz and Cohen 2009). However, the number of possible CRE combinations that could interact to regulate gene expression is too large to explore comprehensively with existing experimental techniques. There are approximately 200 transcription factors (TFs) in *Saccharomyces cerevisiae* for which the DNA-binding specificity is known (de Boer and Hughes 2012; Spivak and Stormo 2012; Hughes and de Boer 2013). If only pairwise interactions between CREs are considered, there are nearly 20,000 possible CRE combinations to evaluate. There is a clear need to efficiently and sensitively identify CRE combinations with nonadditive influence over gene expression.

To address this need, several computational methods have been developed to identify pairs of interacting CREs. When CREs interact to control gene expression, previous evidence indicates that the CREs will cluster near each other in the genome (Pilpel *et al.* 2001; Pramila *et al.* 2002). Efforts to identify combinatorial CRE pairs have exploited this feature by scanning the genome for CRE co-occurrences (GuhaThakurta and Stormo 2001; Chiang *et al.* 2003; Beer and Tavazoie 2004; Das *et al.* 2004; Kato *et al.* 2004; Balaji *et al.* 2006; Krogan *et al.* 2006; Hu *et al.* 2007; Girgis and Ovcharenko 2012; Ha *et al.* 2012; Guturu *et al.* 2013; Kazemian *et al.* 2013; Nandi *et al.* 2013; Jiang and Singh 2014) or by examining ChIP data for TF colocalization (Aguilar and Oliva 2008). However, TF colocalization alone is only a weak indicator of combinatorial regulation (Badis *et al.* 2009) and chance cooccurrence of CREs confound analyzes of single genome sequences. Separate methods have been developed that reduce the number of chance co-occurrences between CREs and enrich for functional CRE interactions by limiting the search space to conserved regions in multiple-species alignments (Chiang *et al.* 2003; Kellis *et al.* 2003; Xie *et al.* 2008; Jiang and Singh 2014). However, aligning promoter sequences from multiple species can eliminate functional binding sites if regulation is not conserved between species or if regulation is conserved but there is turnover of individual sites. This is not a trivial caveat, as comparative genomic studies have revealed extensive gain and loss of CREs between *Saccharomyces* species (Doniger and Fay 2007).

Although individual CREs are often not conserved between species (Hooper *et al.* 2007; Xie *et al.* 2010; Zheng *et al.* 2011; Shibata *et al.* 2012; Reece-Hoyes *et al.* 2013), functional interactions between CREs are often conserved among distantly related species (Tuch *et al.* 2008a; Gerke *et al.* 2009; Cherry *et al.* 2012; Jiang and Singh 2014). Furthermore, studies of gene regulatory evolution have found that interactions between transcription factors are conserved even if the TFs regulate different sets of genes between species (Tuch *et al.* 2008a). Therefore, cooccurrence of *cis*-regulatory elements in multiple unaligned genomes can be used to identify interacting CREs. Incorporating this feature into a prediction method avoids many of the limitations inherent to previous strategies.

We have developed a computational method for identifying coregulatory CREs and provide strong evidence that conservation of a spacing bias between CREs, that is observed in multiple species, indicates combinatorial gene regulation. We use this observation to identify many new instances of significantly co-occurring CREs and to predict combinatorial *cis*-regulation in the yeast genome. We tested the accuracy of our predictions using ChIP-Seq to assay DNA occupancy genome-wide for a few TFs predicted to interact in our computational screen. We made knockout strains of the predicted cofactor and assayed TF occupancy in this cofactor deletion strain. These experiments show that TF occupancy is dependent on the predicted cofactor at specific promoters, but not genome-wide. Finally, we examined the role of CRE combinations in predicting regulatory differences between species. Attempts to predict regulatory divergence genome-wide have generally found little correlation between CRE gain/loss and gene expression (Zhang *et al.* 2004; Tirosh *et al.* 2008). However, gain/loss of CRE combinations can better explain species-specific differences observed in gene expression data.

## Materials and Methods

### Multi-species analysis of CRE co-occurrence

Position weight matrices (PWMs), curated from 11 different literature sources that describe the DNA-binding specificity of 196 *S. cerevisiae* transcription factors (TFs), were obtained from the ScerTF database (Spivak and Stormo 2012). PWMs were adjusted to account for the genome composition for each species. We then predicted binding sites (CREs) within the genomes of *S. cerevisiae* and six other *sensu stricto* and *sensu lato Saccharomyces* species: *S. bayanus*, *S. castellii*, *S*. *kluyveri*, *S. kudriavzevii*, *S. mikatae*, and *S. paradoxus*. The use of multiple species increases the sample size and therefore makes it easier to detect co-occurring CREs. This method is similar to that of Chiang *et al.* (2003) but differs in two important ways. They used word (hexamer) pairs whereas we use PWMs which should increase the sensitivity by better modeling the specificities of TFs. In addition we do not use alignments between species and are not requiring that the occurrences are orthologous, although we expect that many of them are and that such occurrences increase the signal-to-noise and allow us to find CRE pairs that differ between species. CREs were predicted as DNA sites within 25-fold of the consensus sequence predicted affinity, based on the PWM. This is a conservative cutoff that will miss some functional sites (Tanay 2006) but it reduces the false positive rate compared to more relaxed cutoffs. For each pair of PWMs, we calculate the co-occurrence of CREs within each promoter region, defined to be 600 bp upstream of each coding region, in each genome. The observed pattern of CRE co-occurrence in a genome is recorded as a distribution of spacings between CRE pairs in every promoter.

If two CREs interact, evidence indicates that the distribution of spacings between CREs will be skewed toward shorter distances (Drazinic *et al.* 1996; Krogan *et al.* 2006; Tirosh and Barkai 2007). In order to take advantage of this observation, we developed a genome simulation method to determine the expected distribution of CRE spacings while maintaining the total occurrence for each CRE and the spatial localization of CREs within each promoter because those are not randomly distributed (functional sites are more common near the promoter than far away) (Sarafova and Siu 2000). We do this by using permutations that shuffle the CRE annotation associated with each predicted binding site to maintain the number of binding sites associated with each TF and the number and locations of binding sites for every promoter. This shuffling procedure is conducted 1000 times, and the resultant distributions are combined to produce an average expectation.

As expected, the number of co-occurrences in the simulations with a motif spacing of *d* is closely approximated (see Supporting Information, Figure S1) by:

$$S\left(d\right)=\;\frac{2*N(B-d)}{B^{2}}$$

where *N* is the observed number of CRE co-occurrences in the genome and *B* is maximum possible distance between regulatory elements on a promoter (here defined as 600 bp, minus the combined length of the two CREs being evaluated). Having shown that the observed data are well modeled by the formula, we directly test the likelihood of the observed data under a Poisson model with mean and variance parameterized by *S(d)* to determine if the number of observed co-occurrences for a CRE pair is significantly more than expected. We use 25 bp as the limit between CREs to consider them to be interacting. In this step, we apply a Bonferroni correction to account for the multiple hypotheses tested. CRE combinations that do not significantly co-occur (*P* > 0.01) in this step are removed from the analysis.

The subset of CRE pairs that were found to significantly co-occur in multiple *Saccharomyces* genomes when compared against the genome-wide null model were then compared against a null model derived from promoter-by-promoter simulations of CRE co-occurrence. These simulations are conducted similarly to the genome simulations described above, with the exception that these simulations permute the predicted binding sites at each promoter independently. In this way the number of co-occurrences of each CRE pair within all of the promoters is constant between the simulations and the observed data, and we can examine explicitly the intermotif spacing distribution between CREs in greater detail. Although the individual promoter simulations can be time-intensive, most of the possible CRE combinations are removed in the first step of the analysis, which dramatically reduced the search space. The promoter simulations are necessary to distinguish CREs that co-occur near each other from CREs that regulate a common set of genes but are independently distributed at those genes. We compare the observed distribution to the expected distribution derived from 1000 simulation experiments using a chi-square test. The genome-wide analysis examines both CRE spacing and co-occurrence, while the promoter simulations only examine the spacing between CREs. The promoter simulations correct for a source of bias inherent in the genome-wide analysis. Therefore, a multiple hypothesis correction was not applied in this step.

### Corroborating evidence

#### ChIP-chip analysis:

That two predicted CREs occur near one another more frequently than expected does not necessarily mean that they interact to affect gene expression. Immunoprecipitation experiments provide corroborating evidence that TFs are actually bound to the predicted CREs. If two TFs coordinately regulate a set of genes, then both factors need to bind the promoters of those genes. A notable compilation of experiments was conducted by Harbison *et al.* (2004) who collected data for over 100 yeast TFs under several different growth conditions. We also analyzed an earlier ChIP-chip dataset (Arbeitman *et al.* 2002) and a more recent ChIP-chip dataset (Venters *et al.* 2011). A hyper-geometric test was used to determine if a significant number of probes are bound by both TFs for a candidate CRE pair. ChIP occupancy data provides evidence that two TFs both bind to the same promoters in the same environmental growth condition.

#### Target gene expression analysis:

CRE combinations that functionally interact to coordinately regulate target gene expression should generate similar expression profiles among the genes they regulate (Pilpel *et al.* 2001). We calculate the similarity of expression profiles for predicted coregulated genes and for genes predicted to be regulated by only one of the CREs in a pair to assess a functional consequence from the CRE co-occurrences.

Three expression datasets were used to determine if predicted target genes of both CREs were coexpressed across multiple cell cycle time points (Pramila *et al.* 2006), environmental conditions (Gasch *et al.* 2000) or gene deletion conditions (Hughes *et al.* 2000). For each dataset, a Pearson’s correlation coefficient (PCC) was calculated between gene expression profiles for all pairs of predicted target genes, which produced a distribution of PCC values describing the expression profile similarities of the target genes. This distribution of PCC values for predicted targets of the CRE combination was compared with the distribution of PCC values calculated for expression profiles of the targets in which each CRE was predicted to act in isolation. The distribution of PCC values for predicted target genes of both CREs is compared to the two distributions of PCC values for predicted targets of only one CRE using a one-sided Mann–Whitney–Wilcoxin test.

An alternative way to employ gene expression data to identify relationships between TFs is to ask whether a similar set of target genes is significantly up/downregulated in deletion mutants for each of the TFs. Reimand *et al.* (2010) undertook an analysis to identify differentially expressed genes in TF perturbation experiments (Alon 2007). This provides data of potential regulatory target genes for each TF in our analysis. For each CRE combination with a conserved spacing bias, we determined whether there was a significant overlap between target gene sets in TF perturbation experiments using a hyper-geometric test.

#### Target gene pathway analysis:

Previous analyzes of coexpressed genes have found that when a set of genes is coregulated by a combination of TFs, the genes are often involved in a common process or even share a common protein complex (Pilpel *et al.* 2001; Breitkreutz *et al.* 2008). Therefore, one way to corroborate a CRE pair identified from the co-occurrence screen is to determine if the combination regulates a set of genes with a common biological process. The target genes identified in the co-occurrence screen are used here to define a set of genes with binding sites for both TFs in a potential CRE pair. These predicted target genes are examined to determine whether the genes share a common biological pathway. The GO process (Ashburner *et al.* 2000) and KEGG pathway (Kanehisa and Goto 2000) databases are queried with the target gene set to retrieve all the processes associated with each target gene. A hyper-geometric test is used to determine whether the target genes share a common pathway or process. Target genes may have multiple annotations, so a Bonferroni correction is applied for all of the annotations associated with the target gene set.

### Experimental tests of interactions

#### Yeast strains and growth conditions:

Yeast strains with *c-myc* epitopes fused to the C-terminus of the TFs assayed in this study were obtained from the Young Lab (Harbison *et al.* 2004). For each of the assayed TFs, a knockout strain was generated in which the predicted cofactor was replaced with a kanamycin resistance marker obtained from the yeast deletion collection (Giaever *et al.* 2002). Alleles in the knockout strain were replaced using the yeast gene deletion collection strains as a template with the PCR-based recombination strategy detailed by Giaever *et al.* (Giaever *et al.* 2002).

NRG1-myc, SUT1-myc, and SWI4-myc strains were grown at 30° in yeast, peptone, dextrose(YPD)-rich media to exponential midlog phase (OD_600_ ∼0.8). GCN4-myc and RTG3-myc strains were grown in YPD-rich media to OD_600_ ∼0.7, after which rapamycin was added to the media to a final concentration of 100 nM and the cultures were harvested after 20 min (Harbison *et al.* 2004). Strains were grown in 1 l volumes and subsequently split into three equal volumes for chromatin immunoprecipitation. These strains, which harbor both an epitope-tagged transcription factor and a deletion mutant, were assayed in biological triplicate and grown separately in 330 ml culture volumes.

#### Chromatin immunoprecipitation:

Chromatin immunoprecipitations were performed essentially as described previously in the literature (Aparicio *et al.* 2004). However, slight modifications were made to the existing protocol to improve yield and reproducibility. Cell cultures were grown to midlog phase (OD_600_ ∼0.8) and cross-linked in a final concentration of 1% formaldehyde for 15 min. The reaction was quenched with 150 ml 2.5 M glycine (50 ml for the 330 ml cultures) and incubated at room temperature for 10 min. The cell cultures were centrifuged at 2000 × g at 4° for 10 min in a Sorvall RC58 centrifuge. This pellet was washed twice with deionized, distilled H_2_O and recentrifuged. The final pellet was frozen at −80° overnight.

A cell extract was prepared by first adding lysis buffer (Tachibana *et al.* 2005) with protease inhibitor to the frozen pellet and transferring the cell suspension to a 2-ml flat-bottomed screw-cap tube. Zirconia beads (0.5 mm diameter) were added to each tube, and cells were lysed in a beadbeater (BioSpec) set to maximum power for 6 × 5 min cycles with a 2 min rest on ice between cycles. This lysate was transferred to a 15 ml conical tube using the hot-needle transfer method (Aparicio *et al.* 2004) and the volume of the lysate was increased to 5 ml with lysis buffer. The lysate was then sonicated with a Branson Sonifier 250 tip sonicator set to maximum output for 8 × 30 sec cycles with 2 min rest in an ice/ethanol bath between cycles. The lysate was preclarified by centrifugation for 3 min at 3000 × g, and then transferred to microcentrifuge tubes and clarified by centrifugation for 7 min at 10,000 × g. The supernatant was collected (approximately 4 ml) and used for immunoprecipitation. At this step, a 250 μl sample was removed and labeled as the INPUT sample.

Immunoprecipitations (IPs) were performed using anti-c-myc resin (anti-Myc EZiew affinity gel; Sigma-Aldrich). Each 4 ml sample was split into 4 × 1.7 ml microcentrifuge tubes and 50 μl resin was prepared for each microcentrifuge tube (200 μl total for each IP). Resin was washed three times with lysis buffer before use. Samples were incubated by inverting for 14−16 hr at 4°. Samples were then centrifuged for 30 sec at 400 × g. Each sample was washed six times with 1 ml of the following buffers: one lysis buffer wash, one high-salt buffer wash, two wash buffer washes, and two TE (pH 8.0) washes. Finally, samples were eluted by adding 250 μl elution buffer and incubating at 70° for 15 min. From this sample, 200 μl was removed and an additional 100 μl elution buffer was added to the resin. The samples were incubated at 70° for an additional 15 min and 100 μl was removed from the sample and pooled with the first elution. Eluates from the four microcentrifuge tubes per sample were pooled for a final volume of 1.2 ml ChIP elution. This was labeled as the IP sample and incubated overnight at 70° to reverse cross-links. For the INPUT sample, 250 μl elution buffer was added to the aliquot saved earlier and this sample was also incubated overnight at 70°.

After cross-link reversal, the IP sample was concentrated to approximately 500 μl with a vacuum microcentrifuge. Both the IP and INPUT samples were RNAse treated by adding 1 μl 20 mg/ml RNAse and incubating at 40° for 30 min. DNA was then isolated by phenol: chloroform extraction. This DNA was precipitated with 1 ml 100% isopropanol and stored overnight at 4°. The samples were then centrifuged for 1 hr at max at 4°, washed with 75% ethanol, and then recentrifuged for an additional hour at max 4°. The supernatant was discarded and the pellet was resuspended in H_2_O.

These samples were then prepared as libraries for Illumina sequencing (Lefrancois *et al.* 2009). After an end-repair reaction, an adenosine nucleotide was added to the 3′ end of each strand and sequencing adapters were ligated to the DNA fragments. Fragments were size selected (200−600 bp) and amplified with 15 cycles of PCR. Libraries were sequenced using the Illumina HiSeq-2000 in 42 bp single-read runs (data available in NCBI GEO database: GSE60281).

#### Chip-Seq peak analysis:

The multiplexed sequencing data were then deconvoluted using the indexing barcode and aligned to the yeast genome with Novoalign (Novocraft Technologies). If a sequenced fragment did not uniquely align to the genome it was discarded. Gene promoters were defined as the 600 bp immediately upstream of the translational start site of each gene defined in the *Saccharomyces* Genome Database. The number of fragments that aligned to these annotated promoters was recorded for each INPUT and IP sample. This converted the data from read alignments to a table of read counts per promoter.

Transcription factor regulatory targets were determined from the wild-type ChIP-Seq experiments. Regulatory targets were determined separately for each of the biological triplicates using the MACS peak-finding algorithm (Thurman *et al.* 2012). MACS uses a simple sliding window strategy to compare INPUT and IP samples at each position along a chromosome. The algorithm assumes that the number of reads aligned to any particular window is a Poisson process, so the null hypothesis is that the number of reads that align to the current window in the IP sample can be modeled by a Poisson distribution parameterized using the number of reads that align to that same window in the INPUT sample. Regions with a significantly greater number of reads than expected from the INPUT sample are called ‘peaks’ and denote regions of the genome that are bound by the assayed DNA-binding protein. The peaks identified by MACS were used to annotate target genes of the assayed transcription factor; if the MACS peak overlapped with the promoter of a gene, that gene is assumed to be a target of the assayed transcription factor.

Although peak identification was conducted separately for each replicate, annotation of target genes relied on consistency between replicates. Target genes were sorted by support from the peak-finding results for the individual replicates; genes with support from at least two replicates were used to identify joint targets of the TF combination. Gene promoters that were significantly bound in both wild-type strains for a CRE pair were defined as TF combination target genes. In the differential occupancy analysis described below, the statistical test employed is sensitive to sample size. Therefore, the target gene sets defined for each TF binding DNA in isolation were restricted to be the size of the combination target gene set. As such, only the most significant independent target genes from the peak-finding analysis were used to define the genes included in the single TF target gene sets. This analysis of wild-type ChIP data analysis yields, for each TF pair, three equal size gene sets: TF1+TF2 targets, TF1 only targets, and TF2 only targets.

Once the target gene sets were defined for the TF pair and each TF acting in isolation, we examined the difference in occupancy between the wild-type and cofactor deletion strains for each of the three different target gene sets. For each target promoter, we calculated the number of reads that uniquely aligned to that promoter in the INPUT and IP samples and normalized these sums by the total number of million reads in each sample. This calculation transforms the raw read counts to reads per promoter per million mapped. The enrichment ratio for each gene in each IP sample is expressed as the ratio of the IP reads per million mapped divided by the INPUT reads per million mapped. For each gene, we averaged the enrichment ratio across replicates. The cofactor deletion mutant can be considered a “treatment” applied to the target genes for each of the three different gene sets. We would like to determine whether the treatment has an effect on the enrichment ratio (IP/INPUT) for genes within the three different target gene sets. We used a paired T-test to compare the enrichment ratios between wild-type and deletion strains for each gene set. If occupancy of the assayed transcription factor depends on the presence of the predicted cofactor, then the enrichment ratios should be significantly different between the wild-type and deletion strains for the joint targets of the TF combination. If deletion of the cofactor has a more universal effect on the ability of the assayed TF to bind its target promoters, then the enrichment ratios would also be significantly different between treatments for the gene set in which the assayed factor binds promoters without the predicted cofactor.

### Identification of species-specific coregulated genes

Directly comparing gene expression profiles between different species has proven to be a difficult task (Badis *et al.* 2009). Therefore, we took an alternative approach to identify regulatory differences between species. We selected the subset of the significant CRE combinations that predict coherent gene expression patterns in both *S. cerevisiae* and *S. bayanus* to determine if the CRE combinations regulate different sets of genes between the two *Saccharomyces* species.

For each candidate CRE combination, the method identifies potential regulatory targets by scanning each genome separately for instances of the CRE combination within 25 bp of each other using the previously described PWMs (Spivak and Stormo 2012). This produces two sets of potential target genes for each CRE combination; one set contains the predicted targets in *S. cerevisiae* while the other set contains predicted targets in *S. bayanus*. In general, there is substantial overlap between these two sets; predicted target genes in *S. cerevisiae* often have orthologs in *S. bayanus* that are also predicted to be target genes of the CRE combination using the ortholog mapping from Kellis *et al.* (Kellis *et al.* 2003; Harbison *et al.* 2004) to assign a unique ortholog to each gene. The overlap in predicted targets produces three sets of genes: target genes predicted in *S. cerevisiae* but not *S. bayanus*; target genes predicted for *S. bayanus* but not *S. cerevisiae*; and target genes predicted to be regulated by the CRE combination in both species.

#### Initial target gene expression analysis within each species:

In order to assess regulatory rewiring between the two species, we first test whether the CRE combination can be associated with a coherent gene expression pattern within each species. Therefore, as an initial verification that the CRE combination is functional in each of the species, we calculate the similarity of expression profiles for predicted coregulated genes and for genes regulated by only one of the CREs in a pair to infer a functional consequence from the CRE co-occurrences. If a CRE combination regulates a coherent set of genes in one species but not the other, it is possible that this combination is only functional in one of the species. Alternatively, it is possible that the CRE combination is functional in both species but the appropriate conditions were not assayed in one of the two expression datasets.

A CRE combination may not actively regulate gene expression in all or even most of the conditions assayed by the two datasets considered in this study (Gasch *et al.* 2000; Guan *et al.* 2010). Incorporating irrelevant growth conditions into the initial assessment of a CRE combination will obfuscate corroboration of the CRE combination and complicate downstream analyzes. Therefore, it is important to compare the expression profiles of CRE combination target genes to genes regulated by only one of the CREs in a pair using only the appropriate conditions in which the CREs are most likely to be active. We identify the relevant growth conditions for each TF in a regulatory pair by analyzing a collection of expression profiles published for *S. cerevisiae* (Gasch *et al.* 2000) and *S. bayanus* (Guan *et al.* 2010). For each CRE, the relevant growth conditions are identified by determining whether the CRE target genes are significantly differentially expressed in a condition. A CRE target gene is defined as a gene with a PWM match for that TF above specified cutoff. In each condition, the expression ratios reported for every gene are converted to Z-scores, and we use a Z-test to determine whether the CRE target genes are significantly differentially regulated compared to the expression of all genes in that condition.

Once the relevant conditions were selected, we could evaluate each CRE combination in both *S. cerevisiae* and *S. bayanus*. For each dataset, a PCC was calculated between gene expression profiles for all pairs of predicted target genes in each species, which produced a distribution of PCC values describing the expression profile similarities of the target genes. This distribution of PCC values for predicted targets of the CRE combination was compared with the distribution of PCC values calculated for expression profiles of the targets in which each CRE was predicted to act in isolation. The distribution of PCC values for predicted target genes of both CREs is compared to the two distributions of PCC values for predicted targets of only one CRE using a one-sided Mann–Whitney–Wilcoxin test.

#### Comparison of species-specific gene expression profiles:

The search for transcriptional rewiring is a search to identify species-specific gene regulation. In the first part of the analysis, we identified CRE combinations that coordinately regulate target gene expression in each species separately and then predicted species-specific targets of the CRE combination. Afterward, the algorithm tests the hypothesis that the predicted species-specific target genes are coherently expressed in the appropriate species while the orthologs of these targets without the CRE combination are not coherently expressed in the partner species.

This procedure generates three sets of genes predicted to be regulated by the CRE combination under consideration: *S. cerevisiae*-specific target genes (set A), *S. bayanus*-specific target genes (set C), and species-independent target genes (set B) (see Figure 5 for a graphical description). The species-independent target genes should be regulated by the CRE combination in both *S. cerevisiae* and *S. bayanus*. This set of genes provides a benchmark against which we can evaluate the species-specific target genes for *S. cerevisiae* and *S. bayanus*. Within *S. cerevisiae*, the *S. cerevisiae*-specific target genes should have a similar expression pattern to the shared target genes, while the *S. bayanus*-specific target genes should not have a similar expression pattern because those genes are not predicted to be regulated by the CRE combination in *S. cerevisiae*. The opposite pattern should emerge when analyzing the *S. bayanus*-specific target genes using the *S. bayanus* gene expression dataset.

The null hypothesis is that there is no transcriptional rewiring between *S. cerevisiae* and *S. bayanus*, in which case there should not be any species-specific target genes regulated by this CRE combination. If the null hypothesis is true, then the gene sets designated as *A* and *C* are simply false predictions. Either the “species-specific” target genes are not regulated by the CRE combination or they are actually shared target genes regulated by the CRE combination in both species. If either scenario is true, then within each species, the expression profiles of both *A* and *C* will be equally similar to the expression profile of *B*. If the expression profiles of *A* and *C* are equally similar to the expression profile observed for *B*, then we can combine *A* and *C* and randomly sample from this pool to generate simulated “species-specific” gene sets, A* and C*. The simulated gene sets A* and C* are each the same size as *A* and *C*, respectively; only the composition of the gene sets has been shuffled. We compare the expression profiles of the simulated gene set to *B* by calculating PCC between each gene in the simulated set and every gene in *B*. This generates a distribution of PCC that compares the simulated gene set to *B*. If the null hypothesis is true, then the correlation of A* *vs.* *B* should be similar to the correlation of *A* *vs.* *B* in *S. cerevisiae*, and the correlation of C* *vs.* *B* should be similar to the correlation of *C* *vs.* *B* in *S. bayanus*.

If there is support for regulatory rewiring between *S. bayanus* and *S. cerevisiae*, then the species-specific designations are meaningful. As a consequence, the profile comparison between *A* and *B* in *S. cerevisiae* should have a higher mean than the profile comparison between A* and *B*. Similarly, in *S. bayanus*, set *C* should be more similar to *B* than set C*.

We conduct this simulation 1,000 times to estimate the probability that the predicted species-specific gene sets have the observed expression coherence with the shared target genes by chance. A simulation is counted as successful if the correlation of A* *vs.* *B* is greater than the correlation of *A* *vs.* *B* using the *S. cerevisiae* expression dataset and the correlation of C* *vs.* *B* is greater than the correlation of *C* *vs.* *B* in the *S. bayanus* expression dataset. We estimate the probability that the observed expression profile similarities occurred by chance as the number of successful simulations divided by the total number of simulations.

#### Biological pathway analysis:

A CRE combination that regulates different sets of genes between related species might regulate different biological processes. To investigate this possibility, we assessed the biological pathway enrichment of the three different sets of target genes defined for each CRE combination. An overrepresentation of genes associated with a specific pathway in *A* but not *B* and *C* suggests that the CRE combination regulates that pathway only in *S. cerevisiae*. Similarly, pathway enrichment apparent in *C* but not *A* and *B* indicates *S. bayanus*-specific regulation of that pathway.

We determined pathway enrichment for each gene set using the Gene Ontology database of biological processes (Ashburner *et al.* 2000) and KEGG database of biological pathways (Kanehisa and Goto 2000) to retrieve all the processes associated with each target gene. A hyper-geometric test is used to determine whether the target genes share a common pathway or process. Target genes may have multiple annotations, so a Bonferroni correction is applied for all of the annotations associated with the target gene set.

### Data availability

ChIP-seq data accessible from NCBI GEO database: GSE60281.

## Results and Discussion

### Multiple-species spacing bias predicts combinatorial function of CRE pairs

PWMs curated from the literature (Spivak and Stormo 2012) were used to identify potential binding sites for 196 TFs in the genomes of *S. cerevisiae*, *S. bayanus*, *S. castelli*, *S. kluyveri*, *S. kudriavzevii*, *S. mikatae*, and *S. paradoxus*. For each pair of PWMs, we calculated the distribution of nucleotide spacings between the predicted binding sites and identified pairs for which the observed distribution deviated significantly from random expectation (see *Materials and Methods*). The co-occurrence screen identified 1399 CRE combinations, 7.3% of the 19,110 possible, that exhibit a conserved spacing bias across multiple *Saccharomyces* genomes (*P* < 0.01 after correction for multiple tests). This collection includes many known examples of combinatorial *cis*-regulation, demonstrating that the screen can successfully identify genuinely functional TF interactions (Table S1). Furthermore, although the screen did not require that the CRE combinations occur in every species, in almost all cases they are observed in each of the seven species and usually with similar frequencies (Table S2).

One example of a known case is the highly significant interaction identified between PAC and RRPE elements in ribosomal genes, which are recognized by the TFs Pbf2 and Stb3 (Pilpel *et al.* 2001; Liko *et al.* 2007; Zhu *et al.* 2009). Our analysis also found that the STB3 motif significantly co-occurs with several other motifs involved in the cell cycle (MBP1, SWI4), metabolism (GCN4), and stress response (XBP1). Previous studies have identified a role for STB3 in the transcriptional regulation of both cell cycle (Tavazoie *et al.* 1999) and stress response (Gasch *et al.* 2000) genes, indicating that the co-occurrence screen has likely identified functionally relevant CRE interactions.

### Corroborating evidence

#### Eighty per cent of predictions have corroborating experimental support:

**Figure 1:**
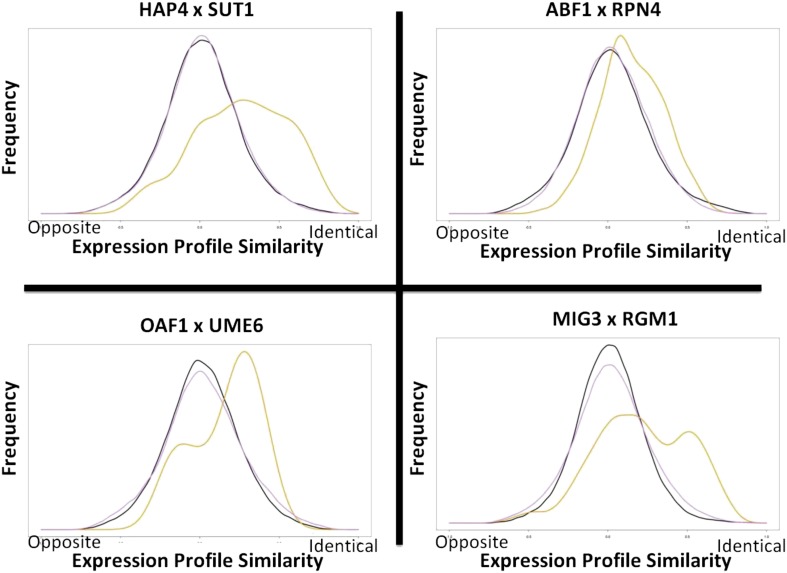
Expression profiles of predicted CRE combination target genes are more correlated than predicted target genes of either CRE acting alone. The yellow line in each graph depicts the distribution of correlation coefficients calculated between gene expression profiles for each pair of target genes predicted to be regulated by the CRE combination indicated. The black and purple lines relate the distribution of correlations for target genes predicted to be regulated by each CRE acting alone. “Opposite” refers to a correlation of −1 and “Identical” refers to a correlation of +1.

There are three main features that distinguish CRE combinations from independent CREs. First, when TFs coordinately regulate a set of genes, both factors bind the promoters of those genes (Harbison *et al.* 2004). Second, interactions between CREs often produce nonadditive changes in gene expression (Shea and Ackers 1985; Pilpel *et al.* 2001). Third, genes that are coordinately regulated by a particular combination of TFs often share a common biological process (Pilpel *et al.* 2001; Banerjee and Zhang 2003). We analyzed existing ChIP-chip (Arbeitman *et al.* 2002; Harbison *et al.* 2004; Venters *et al.* 2011), gene expression (Gasch *et al.* 2000; Hughes *et al.* 2000; Pramila *et al.* 2006; Reimand *et al.* 2010), and biological pathway data (Ashburner *et al.* 2000; Kanehisa and Goto 2000) to identify corroborating experimental evidence supporting the computational predictions from our phylogenetic analysis (see *Materials and Methods*). Of the 1399 pairs of CREs that co-occur in multiple yeast species, 1121 CRE pairs, representing approximately 80% of the computational predictions, have at least one type of experimental evidence supporting the prediction (*P* < 0.01 in at least one corroborative analysis). Approximately 36% of the predictions are supported by at least two different types of experimental evidence and 8% of the predictions are corroborated by all three experimental methods (Table S1). The number of examples for each type of supporting experimental data depends on the specific thresholds used. But given those sets we can ask whether the specific combinations are significantly overrepresented. In fact all of the combinations are significant at *P* < 0.05, and for the combination of Chip-chip colocalization and pathway enrichment, and for the combination of all three types of data, the significance is *P* < 10^−3^. Figure 1 shows four examples with gene expression corroborating evidence. In those examples the genes that have both of the CREs show much more coherent expression than genes that have either of the two CREs alone.

Most known examples of combinatorial *cis*-regulation come from a handful of thoroughly studied biological processes (*e.g.*, cell cycle, starvation, *etc*.) or have been inferred from high-throughput genetic screens (Krogan *et al.* 2006; Fordyce *et al.* 2010). To identify condition-specific CRE pairs, we analyzed target gene expression coherence in three different gene expression datasets: a cell cycle time course experiment (Pramila *et al.* 2006), a series of growth experiments in multiple environmental stress conditions (Gasch *et al.* 2000), and a compendium of gene deletion mutants (Hughes *et al.* 2000). Many of the most significant interactions discovered from the cell cycle time course experiments are well known interactions involving the cell cycle regulators SWI4, SWI6, and MBP1 and are listed in the *Saccharomyces* Genome Database (SGD) (Boyle *et al.* 2012) (Table 1). Our analysis of the environmental and genetic perturbation data, however, mostly identified unknown CRE combinations whose target genes are significantly coexpressed across conditions (Table 2). That most of these interactions have not been documented previously suggests that, unlike the well-studied cell cycle transcriptional network, gene regulation in response to environmental changes remains largely open for new discoveries.

**Table 1:** Most significant CRE combinations from cell cycle time course expression coherence analysis

Pair	Simulation	Cell Cycle	Environment	SGD
MBP1 × SWI6	1.1084E-280	<1e-300	4.49728E-69	Yes
PBF2 × STB3	0	5.4172E-270	<1e-300	No
MBP1 × SWI4	4.40017E-73	3.0837E-266	1.0856E-111	Yes
MBP1 × STB1	4.14077E-89	3.0406E-188	7.03385E-78	Yes
SWI4 × SWI6	5.21369E-93	1.3756E-175	1.1981E-154	Yes
STB2 × STB3	1.1218E-82	2.23188E-95	1.77025E-50	No
PBF1 × STB3	0	6.29925E-89	<1e-300	No
STB1 × SWI6	2.1118E-97	4.00616E-87	5.4427E-121	Yes
REB1 × STB3	8.5209E-128	2.09431E-76	1.83331E-43	No

**Table 2:** Most significant CRE combinations from environmental stress expression coherence analysis

Pair	Simulation	Cell Cycle	Environment	SGD
PBF2 × STB3	0	5.4172E-270	<1e-300	No
MIG3 × RGM1	3.00368E-18	2.24847E-19	<1e-300	No
GIS1 × MIG3	1.5942E-17	2.24847E-19	<1e-300	No
MIG3 × YPL230W	3.87555E-17	2.24847E-19	<1e-300	No
GIS1 × SUT1	4.89402E-16	1.06326E-10	<1e-300	No
RGM1 × SUT1	6.23916E-16	1.06326E-10	<1e-300	No
SUT1 × YPL230W	3.34119E-14	1.06326E-10	<1e-300	No
MSN4 × SUT1	6.48628E-10	1.31691E-36	<1e-300	No
MIG3 × MSN4	1.7635E-09	6.40281E-29	<1e-300	No

#### Physical constraints of CRE combinations:

TFs that bind cooperatively to DNA sometimes exhibit a strong bias in the relative position and orientation of their binding sites (Pramila *et al.* 2002). Previous studies have found that positional constraints on CREs can be important determinants of gene expression patterns (Sudarsanam *et al.* 2002). Therefore, a CRE combination with a conserved pattern of binding site arrangements may indicate that the orientation or order of these binding sites influences gene regulation. After identifying CRE combinations that co-occur more than expected by chance, we further analyzed the results from our co-occurrence screen to detect biases in the physical arrangement of CREs for co-occurring CRE pairs. Specifically, we looked for CRE combinations in which one particular orientation or order of binding sites occurred more than expectation and then compared the expression coherence between regulatory targets with the preferred arrangement of binding sites and targets with a different binding site arrangement.

**Figure 2:**
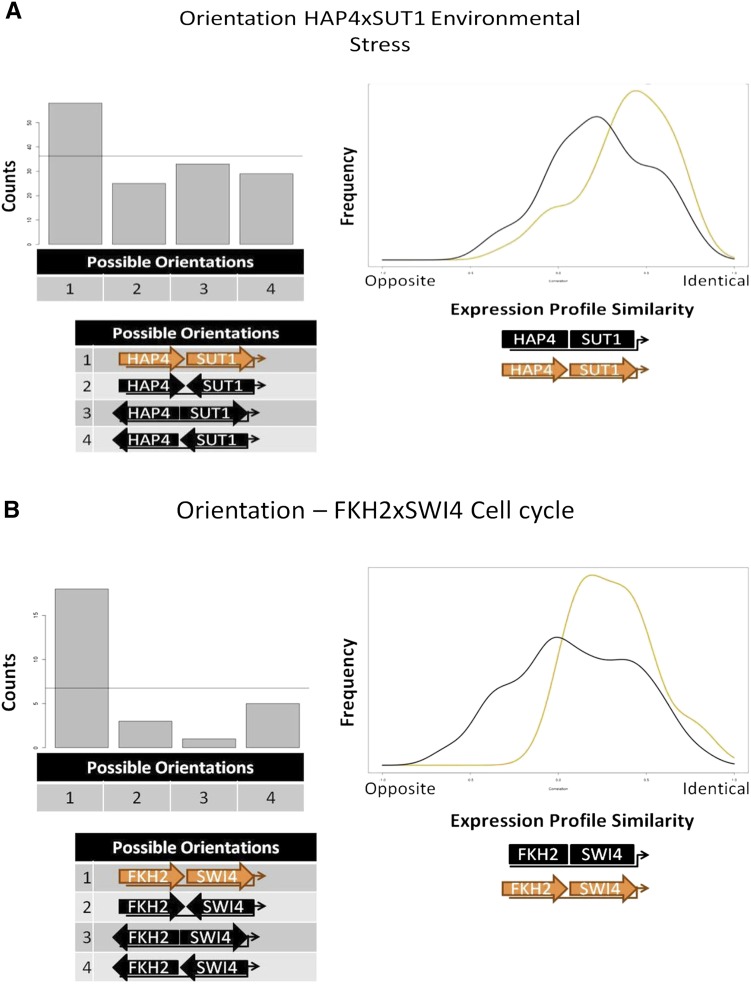
Orientation biases for CRE combinations. The pattern of occurrences in multiple species for HAP4 and SUT1 CREs (A) and for FKH2 and SWI4 CREs (B) indicates an overrepresentation of Orientation 1 (depicted in orange). The horizontal line crossing the bar graph represents the expected number of occurrences for each orientation if orientation is random. The expression profile plot depicts the distribution of correlation coefficients calculated between gene expression profiles for target genes with the overrepresented orientation (orange) and all other orientations (black).

HAP4 and SUT1 is an example of previously undescribed interaction with a preferred orientation (Figure 2A). Interestingly, this CRE combination may only be active in certain environmental conditions. There is no significant overlap between ChIP-chip experiments for HAP4 and SUT1 when cultured in optimal growth conditions (Harbison *et al.* 2004). However, both the expression coherence analysis and the biological pathway analysis support a functional role for the predicted interaction between HAP4 and SUT1. Additionally, the target genes of the HAP4 × SUT1 combination are significantly differentially expressed in oxidative stress conditions and growth on suboptimal carbon sources. After identifying this combination from the co-occurrence screen, we further divided the co-occurrences into each of the four possible orientations and found a significant overrepresentation of one particular orientation among the *Saccharomyces* genomes. We partitioned the target genes of the HAP4 × SUT1 combination into a set with the overrepresented orientation and a set with the three remaining orientations and determined that the set of target genes with the preferred HAP4 × SUT1 orientation were significantly more coherently expressed across environmental conditions than the set of target genes without the preferred binding site arrangement.

Several known examples of combinations of CREs with known positional constraints were identified by our method, including MCM1 × YOX1 (Pramila *et al.* 2002), MCM1 × FKH2 (Pramila *et al.* 2006; Tuch *et al.* 2008b), and PBF2 × STB3 (Sudarsanam *et al.* 2002; Liko *et al.* 2007). We also identified the pair FKH2 × SWI4 which was previously reported to interact to control expression of S phase genes in the cell cycle (Sudarsanam *et al.* 2002) but for which a positional bias had not been reported. Figure 2B shows that one orientation is much more common than the other three and also that the genes with that position bias are expressed much more coherently than the genes with alternative orientations.

### Experimental tests of interactions

#### ChIP-Seq reveals asymmetry in TF combinatorial interactions:

As a preliminary assessment of the experimental strategy we immunoprecipitated Swi6p in a wild-type and swi4Δ strain. Swi4p and Swi6p are the two components of the SBF regulatory complex that control G1 to S phase transition during the cell cycle (Koch *et al.* 1993). Swi6p is not believed to have the ability to bind DNA directly, and its association with DNA is mediated by its various cofactors, which include Swi4p, Mbp1p, and Stb1p (Koch *et al.* 1993; Conlan *et al.* 1999). Therefore, we assayed the SWI4 × SWI6 combination to determine if ChIP-Seq can be used to quantitatively measure occupancy differences between wild-type and cofactor deletion strains. Using the wild-type SWI6::myc18 and SWI4::myc18 strains, we could define the combinatorial targets and independent targets for the TF pair. In the SWI6::myc18/swi4Δ strain, Swi6p should not be able to bind the combinatorial targets of SWI4 × SWI6 because the interaction between Swi4p and Swi6p has been disrupted. However, the individual targets defined for Swi6p, in which a ChIP-Seq peak for Swi6p did not overlap with any ChIP-Seq peaks for Swi4p, should be relatively unaffected by the loss of SWI4. If Swi6p occupancy of these target genes is significantly affected by the deletion of SWI4, then there is a genetic interaction between SWI6 and SWI4 in which SWI4 globally affects the activity of SWI6. In this scenario, a physical dependency cannot be inferred because the results do not divorce physical interactions from genetic interactions.

The results of this initial experiment confirm the utility of ChIP-Seq as a method capable of quantitatively measuring the dependence between two TFs at combinatorial target genes and Swi6p-only target genes. In the wild-type SWI6::myc18 experiments, the IP samples are highly enriched for combinatorial target genes with greater than eightfold enrichment observed for some target genes. However, in the SWI6::myc18/swi4Δ strain, these target genes are no longer enriched in the IP sample, indicating that Swi6p occupancy of these target genes is dependent on SWI4 (Figure S2). A paired-sample Wilcoxin Signed Rank Test comparing the wild-type and deletion experiments reports a significant difference in Swi6p occupancy between the conditions (*P* < 10^−5^). In contrast, the difference between the wild-type and swi4Δ strains was not significant when examining the Swi6p-only targets reported by MACS (*P* = 0.27; Figure S2). It is interesting to note that although most genes do not appear different between the two conditions, Swi6p occupancy of some of these target genes does appear to change between conditions. Of these nine target genes with an occupancy difference between the wild-type and swi4Δ strains, six have a match to the SWI4 binding site in their promoter sequences. One possibility is that these six genes are, in fact, combinatorial genes but were not categorized as such by the MACS peak-finding algorithm. We also examined differences in Swi4p occupancy between wild-type SWI4::myc18 and SWI4::myc18/swi6Δ strains. In general there are only small differences between wild-type and deletion conditions for the combinatorial target genes and the Swi4p-only target genes, as expected (Figure S2).

#### Interaction between NRG1 and SUT1:

The co-occurrence screen identified a significant spacing bias between NRG1 and SUT1 CRE*s* (*P* < 10^−5^). Additionally, predicted target genes of the NRG1 × SUT1 CRE combination were significantly more coherently expressed than expected by chance (*P* < 10^−27^). Based on this data, we investigated the interaction between NRG1 and SUT1 using ChIP-Seq to measure occupancy of Nrg1p in wild-type and sut1Δ strains as well as the reciprocal experiment for Sut1p. As depicted in Balaji *et al.* (2006), Kazemian *et al.* (2013), and Nandi *et al.* (2013), Nrg1p occupancy of NRG1 × SUT1 combinatorial targets depends on the presence of SUT1 (*P* < 10^−6^), while Nrg1p occupancy of Nrg1p-only targets is much less dependent on SUT1 (*P* = 0.011). There is an observable difference in occupancy between the wild-type and sut1Δ strains for approximately five genes in the Nrg1p-only target set; interestingly two of those five genes, snR63 and YDR039C, have a match to the SUT1 binding site but were not identified as bound regions by MACS in the Sut1p ChIP-Seq. Removing these two genes from the Nrg1p-only target set increases the *P*-value for the comparison between wild-type and sut1Δ strains from 0.011 to 0.032.

**Figure 3:**
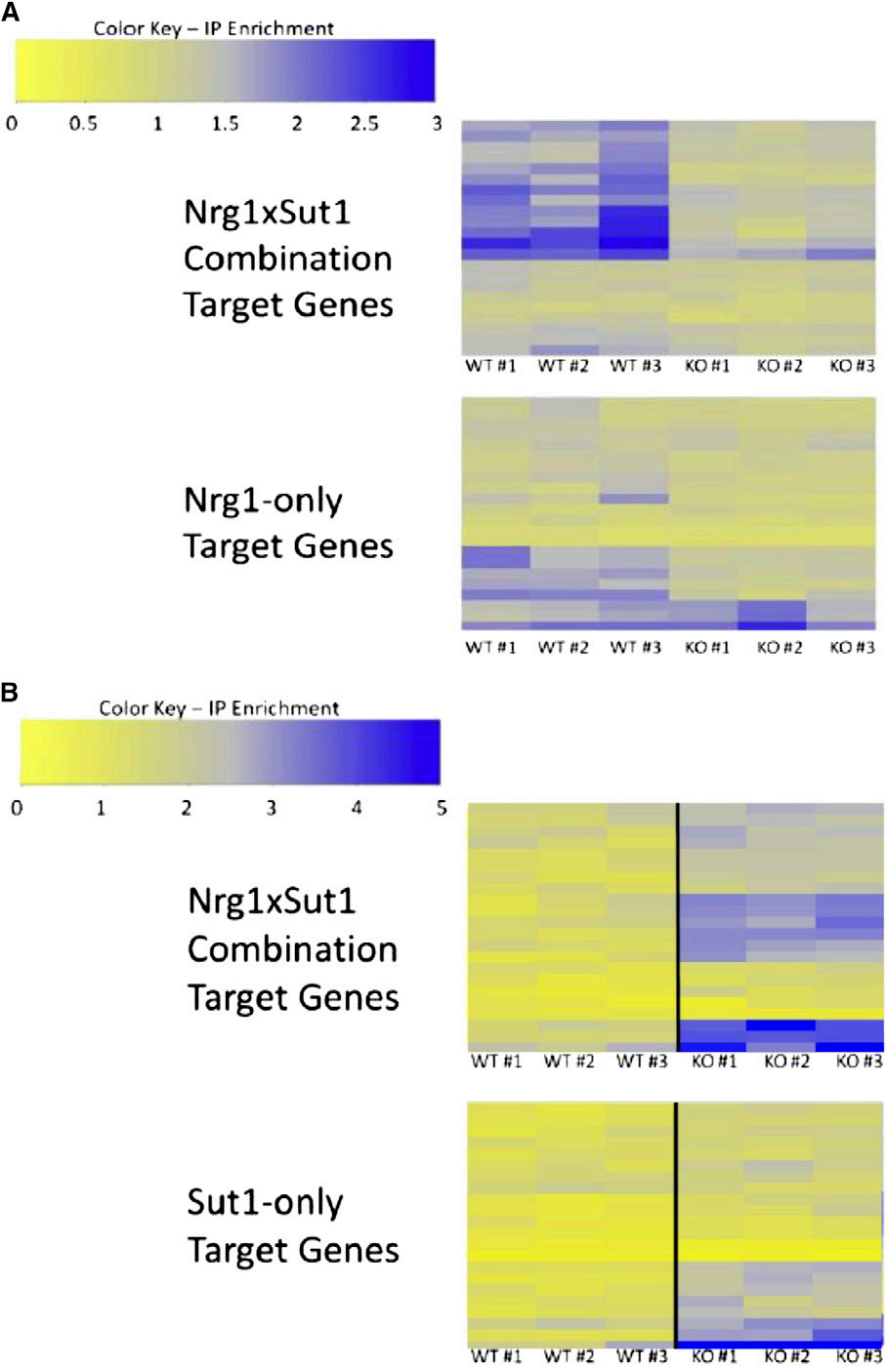
Enrichment ratios from ChIP-seq experiments. Target genes for each genotype (see *Materials and Methods* for details of peak identification). Each of individual experiments from the triplicates is shown (labeled with #). (A) Nrg1 ChIP-seq in *NRG1*::*myc* strain (top) and *NRG1*::*myc/sut1*Δ strain (bottom). (B) Sut1 ChIP-seq in *SUT1*::*myc* strain (top) and *SUT1*::*myc/nrg1*Δ strain (bottom). Gene names are provided in Table S3.

In contrast to the results for the Nrg1, the ChIP-Seq data for Sut1p shows that Sut1p occupancy increases in the nrg1Δ strain (Figure 3B). This trend is significant for both the combinatorial and Sut1p-only target gene sets (*P* < 10^−6^ and < 10^−5^, respectively). In this case, it is impossible to determine if Sut1p physically depends on Nrg1p for promoter occupancy because there is a genetic interaction between SUT1 and NRG1 in which deletion of NRG1 increases the DNA-binding activity of Sut1p. It is unclear how deletion of NRG1 exerts a global effect on Sut1p activity. In both this study and previous studies, Nrg1p does not appear to associate with the promoter of SUT1 under the conditions of our experiment (Harbison *et al.* 2004), and deletion of NRG1 does not significantly affect the expression of SUT1 (Reimand *et al.* 2010). However, the SUT1 promoter is significantly bound by Adr1p in cell cultures shifted to low glucose conditions (Tachibana *et al.* 2005). ADR1 activates expression of genes required for nonoptimal carbon source metabolism in response to glucose starvation (Kim *et al.* 2003). Similarly, NRG1 negatively regulates genes required for nonoptimal carbon source metabolism when glucose is present in the growth media (Mertin *et al.* 1999). One possibility is that direct regulation of SUT1 by ADR1 indirectly links SUT1 and NRG1 through the glucose sensing network.

#### Interaction between GCN4 and RTG3:

Several of the CRE combinations identified in the co-occurrence screen integrate distinct physiological processes of the cell. For one such CRE combination, GCN4 × RTG3, we used the differential ChIP-Seq assay to investigate dependencies between the TFs involved in regulation. GCN4 is a transcriptional activator that induces expression of amino acid biosynthesis genes in response to nutrient starvation (Natarajan *et al.* 2001). RTG3 serves to activate expression of genes involved in the retrograde and TOR (Target Of Rapamycin) pathways (Butow and Avadhani 2004). The retrograde response signals mitochondrial dysfunction to the nucleus and induces changes in carbohydrate and nitrogen metabolism. The TOR pathway couples nutrient sensing to protein synthesis/degradation (Raught *et al.* 2001). Thus, GCN4 and RTG3 regulation should converge in nutrient starvation growth conditions. Indeed, analysis of data from a previous ChIP-chip study (Harbison *et al.* 2004) reveals that the regulatory targets bound by Gcn4p and Rtg3p significantly overlap (hyper-geometric test, *P* < 10^−12^) upon treatment with rapamycin. Rapamycin is an antifungal drug that inactivates TOR signaling in *S. cerevisiae*, which elicits a nutrient starvation response (Loewith and Hall 2011).

**Figure 4:**
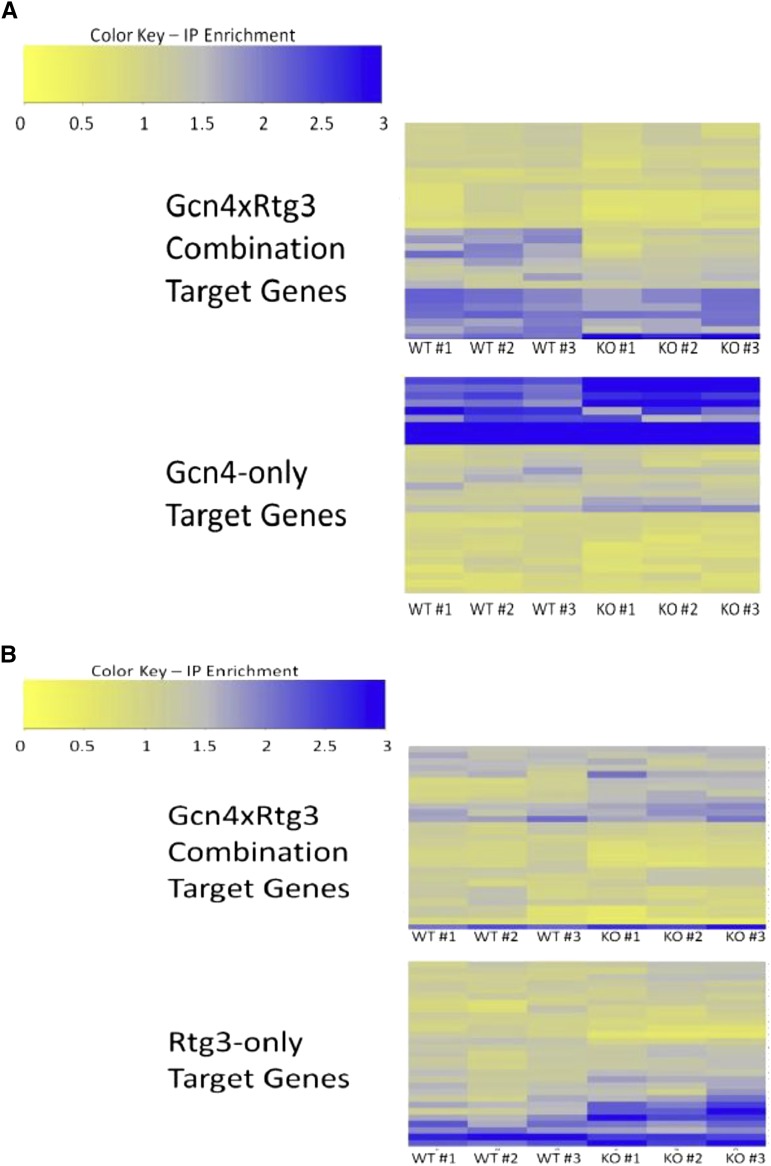
Enrichment ratios from ChIP-seq experiments. (A)Gcn4 ChIP-seq in *GCN4*::*myc* strain (top) and *GCN4*::*myc/rtg3*Δ strain (bottom). (B) Rtg3 ChIP-seq in *RTG3*::*myc* (top) and *RTG3*::*myc/gcn4*Δ (bottom). Gene names are provided in Table S4.

Following the method of Harbison *et al.* (Harbison *et al.* 2004) we treated cell cultures with rapamycin and measured Gcn4p and Rtg3p occupancy in wild-type and cofactor deletion strains. The differential ChIP-Seq experiments show that Gcn4p occupancy of combinatorial target genes is significantly greater in the wild-type yeast strain compared to the GCN4::myc9/rtg3Δ strain (*P* = 0.001) (Figure 4A). The occupancy difference observed for the combinatorial targets is not due to global changes in Gcn4p activity; occupancy of Gcn4p-only targets was not significantly different between the two strains (*P* = 0.6). In contrast, ChIP-Seq analysis of Rtg3p indicates that Rtg3p binding is independent of Gcn4p (*P* = 0.46) (Figure 4B). These data suggest that Gcn4p depends on Rtg3p for occupancy of the GCN4 × RTG3 combinatorial target promoters, but Rtg3p binding is independent of Gcn4p. Similar results have been observed previously for GCN4-mediated gene regulation (Devlin *et al.* 1991). Rap1p binds the HIS4 promoter independently of Gcn4p, but Rap1p binding is required for Gcn4p activation of HIS4 (Devlin *et al.* 1991). In a later study, it was concluded that Rap1p overcomes a repressive chromatin structure at the HIS4 promoter and increases promoter accessibility for Gcn4p (Sierro *et al.* 2008). RTG3 may act in a similar fashion; although Rtg3p can act as a transcriptional activator, components of the SAGA chromatin remodeling complex, Ada2p and Gcn5p, are required for Rtg3p activity (Pray-Grant *et al.* 2002). Rtg3p is also known to physically interact with other chromatin remodeling complexes including SLIK (Pray-Grant *et al.* 2002) and the Tup1-Cyc8 repressor complex (Conlan *et al.* 1999). Additionally, Rtg3p may recruit the RSC nucleosome-remodeling complex (Ng *et al.* 2002). One possible model that accounts for the observed results and is consistent with previous studies involves Rtg3p altering the chromatin state of the CRE combination target genes to permit GCN4 occupancy.

### CRE combinations can identify species-specific gene expression patterns

Studies of interspecies *Saccharomyces* hybrids indicate that expression divergence between species is largely a consequence of differences in *cis*-regulation (Tirosh *et al.* 2009; Bullard *et al.* 2010). The term “rewiring” refers to differences in gene regulatory connections between species that result from variations in *cis*-regulatory content (Tuch *et al.* 2008a; Xie *et al.* 2010; Reece-Hoyes *et al.* 2013). However, despite divergence in promoter sequences between species, orthologous genes often display relatively conserved expression patterns (Weirauch and Hughes 2010). Similarly, gain and loss of CREs between species is only poorly correlated with expression divergence (Tirosh *et al.* 2008). However, most previous studies have only focused on individual CREs.

#### Expression coherence corresponds to co-occurrences of CREs, not individual CREs:

**Figure 5:**
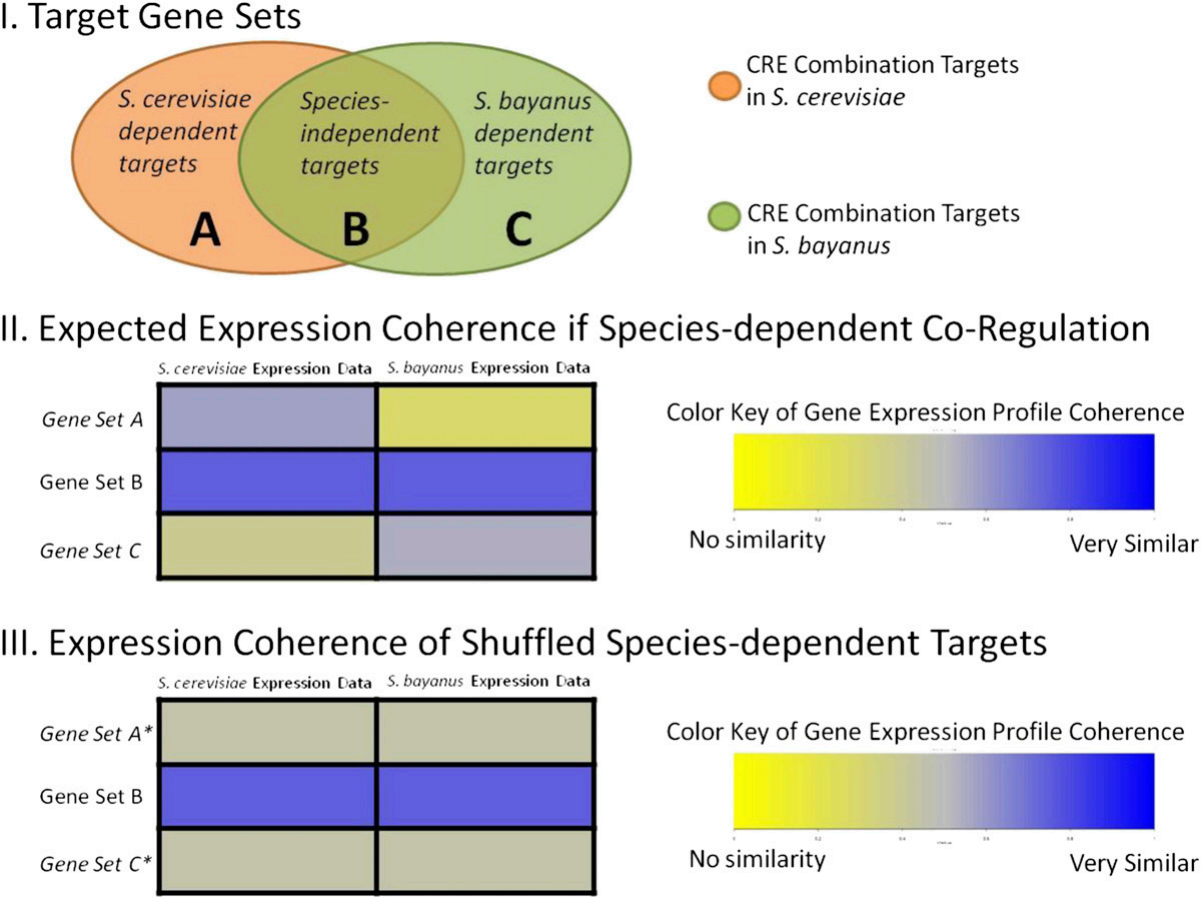
Graphical representation of expression analysis between *S. cerevisiae* and *S. bayanus*. In section I CRE combination target genes are predicted in each species and the overlap defines three target gene sets (A, B, C). Section II shows the average correlation coefficient observed when comparing expression profiles of each gene set with gene set *B* for the two different gene expression datasets. Section III shows a decrease in the average correlation between target gene sets and gene set *B* when genes are randomly assigned to either set *A* or *C* in simulation experiments.

Using the CRE combinations identified in our co-occurrence screen, we searched for examples of rewiring between both *S. cerevisiae* and *S. bayanus* using two criteria. First, there had to be sets of genes containing the predicted CRE combination in both species, and also sets of genes with the predicted CRE combination that were unique to each species; we are specifically looking for gain and loss of genes regulated by the CRE combination in both species. Second, there must be conditions for which gene expression assays demonstrate that the genes with the CRE combination are coherently expressed in both species. Using expression data from several different environmental conditions for both *S. cerevisiae* (Gasch *et al.* 2000) and *S. bayanus* ([Bibr bib29][Bibr bib30]), 275 CRE combinations met both criteria. We then measured the expression coherence in three sets of genes defined by the occurrence of the CRE combination: set A are the genes with the CRE combination only in *S. cerevisiae*; set B are the genes with the CRE combination in both *S. cerevisiae* and *S. bayanus*; set C are the genes with the CRE combination only in *S. bayanus* (Figure 5).

Of the 275 CRE combinations considered, we identified 81 CRE pairs (*P* < 0.05; Table S5) for which the expression profiles of *A* and *B*, but not *C*, were significantly similar in *S. cerevisiae* while *C* and *B*, but not *A*, were significantly similar in *S. bayanus*. This result indicates that the CRE combinations we identified have species-specific regulatory targets in both *S. cerevisiae* and *S. bayanus*. These regulatory targets have similar expression profiles to the species-independent target genes in the appropriate species, while the remaining genes which lack the CRE combination do not display a similar expression profile. In these cases, gain and loss of a combination of CREs between species accurately predicts gain and loss of expression coherence.

**Figure 6:**
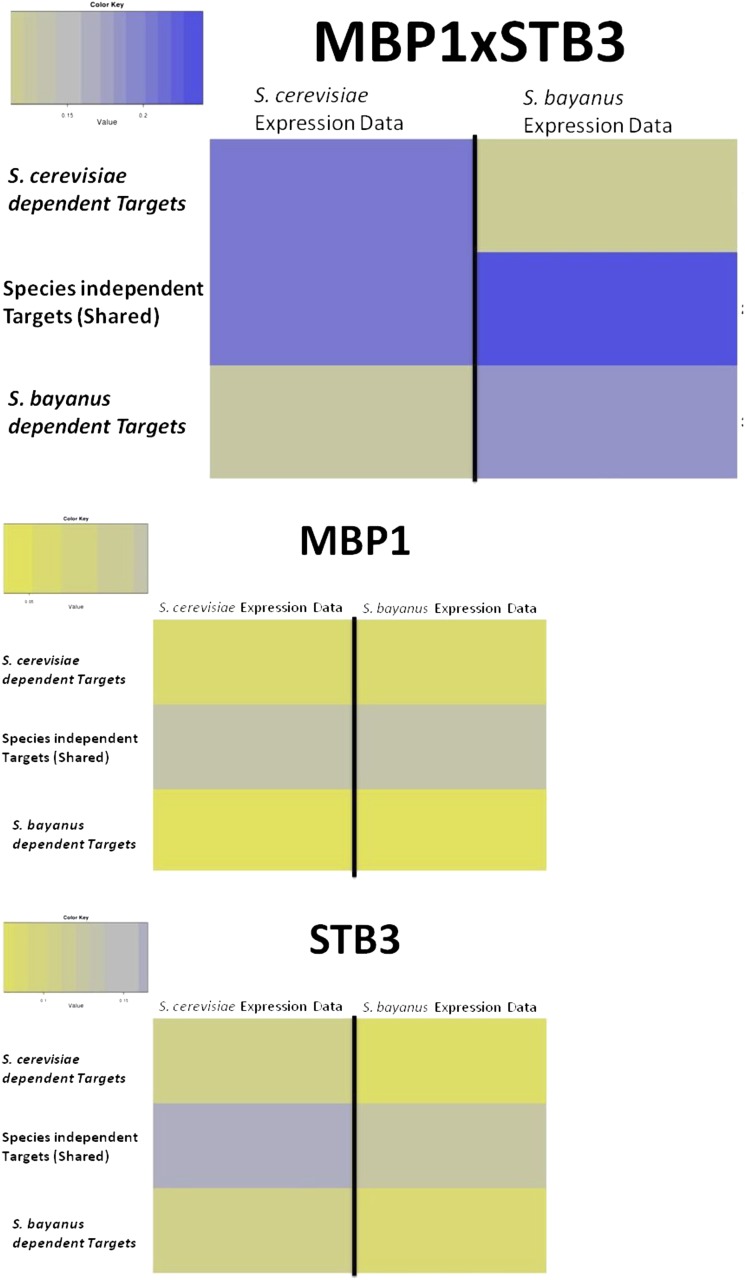
Expression profile similarity between gene sets for each species. Predicted genes regulated by MBP1 and STB3 CREs (top) for both species and all three gene sets (A, B, C), for MBP1-only predicted genes (middle) and for STB3-only predicted genes (bottom).

Figure 6 shows the results for the CRE combination MBP1 × STB3. In *S. cerevisiae* there is much higher expression coherence between the genes in sets A and B than in C, whereas in *S. bayanus* the much higher expression coherence is between genes in sets B and C rather than A.

A comparable analysis using individual CREs to predict species-specific gene regulation in *S. bayanus* and *S. cerevisiae* to determine if gain/loss of individual CREs can predict differences in expression patterns between species shows no such difference in expression coherence (Figure 6). These results mirror the findings of previous attempts to predict genome-wide transcriptional rewiring between species using individual CREs (Zhang *et al.* 2004; Tirosh *et al.* 2008). Figure S3 shows an additional set of 18 pairs that show significant rewiring between *S. cerevisiae* and *S. bayanus*.

#### Species-specific target gene pathway enrichment:

An interesting hypothesis is that the CRE combinations with species-specific targets are responsible for regulating different biological processes within each species. The alternative is that there is no enrichment among the different gene sets for distinct biological processes and the species-specific targets have been acquired at random. We examined the different gene sets defined for each CRE combination to determine if any of the gene sets exhibited enrichment for a particular biological process that was exclusive to that gene set. In fact, for several of the CRE combinations with species-specific regulatory targets, at least one of the gene sets (A, B or C) is significantly enriched for a biological pathway not associated with any of the genes in the other two gene sets (Table S6).

For several of the combinations, more than one of the gene sets has an exclusive biological pathway enrichment. As an example, the CRE combination ARG80 × GCN4 regulates genes associated with arginine biosynthesis (GO term 6526) in both *S. cerevisiae* and *S. bayanus* (*P* < 10^−4^), but in *S. bayanus* the combination is also associated with regulation of lysine metabolism (GO term 9085; *P* < 10^−3^). GCN4 is a master regulator of amino acid biosynthesis (Natarajan *et al.* 2001) and ARG80 is responsible for arginine biosynthesis (Dubois *et al.* 1987), so the association with the GO category for arginine biosynthesis is not surprising. However ARG80 is not known to be associated with lysine biosynthesis, so this *S. bayanus*-specific pathway association could indicate that the regulatory role of ARG80 has expanded in *S. bayanus*.

### Conclusions

The combination of CREs in a promoter is an important determinant of gene expression patterns but we have only a limited understanding of which TFs interact. We have developed a computational approach to determine if a conserved pattern of CRE spacing in multiple, unaligned genomes can predict combinatorial regulation. The ability of this method to recover known CRE combinations indicates that conserved patterns of CRE clustering can be used to infer modular regulatory function, and extensive supporting evidence also indicates the reliability of the method. Experimental tests of two new interacting TF pairs verified the predictions but also showed asymmetry in the binding requirements. Previous methods to infer combinatorial regulation from CRE proximity often only considered a single genome or use multiple-species alignments as a filter to reduce the size of the genome before assessing CRE co-occurrence. By using multiple species but not requiring aligned orthologous promoters we use more extensive data to identify co-occurring TF pairs and can include examples of rewiring of the regulatory network. TF motif degeneracy complicates the detection of functional *cis*-regulatory modules for all methods because many nonfunctional CRE co-occurrences will be observed by chance. This effect could be reduced by using DNA accessibility information, but that is often not available. However, if two CREs cluster together in the genome to coordinately regulate gene expression, these observations occur in addition to the random co-occurrences of any two CREs. With enough observations, a nonrandom pattern of CRE clustering can be more easily distinguished from a random pattern. Differences in the gene sets containing significant TF pairs can indicate evolutionary rewiring events, something that is often difficult to predict reliably using only single TF binding site predictions.

## Supplementary Material

Supporting Information
