# Supplementary material for: Combinatorial Cis-regulation in Saccharomyces Species
Source: G3 (Bethesda). 2016 Jan 12;6(3):653–67. doi: 10.1534/g3.115.024331 (PMC4777128; doi:10.1534/g3.115.024331)
Supplement: Supporting Information [file supp_6_3_653__index.html]

Combinatorial Cis-regulation in Saccharomyces Species — Supporting Information 

# Combinatorial *Cis*-regulation in *Saccharomyces* Species

## Supporting Information for Spivak and Stormo, 2016

**Files in this Data Supplement:**

- Supporting Information - File contains all Supporting Figures and Tables. (.pdf, 3,272 KB)
- Figure S2 - Enrichment ratios are depicted as a heat map for each replicate in *SWI6::myc* and *SWI6::myc/swi4Δ* ChIP experiments. (.pdf, 337 KB)
- Figure S3 - Heat Maps for the most definitive examples of CRE combination rewiring. (.pdf, 405 KB)
- Table S3 - Figure 3 gene names (in order, top to bottom). (.pdf, 235 KB)
- Table S4 - Figure 4 Gene names (in order, top to bottom). (.pdf, 176 KB)
- Table S5 - 81 CRE pairs with significant (P<0.05) rewiring. (.pdf, 236 KB)
- Table S6 - GO pathway enrichment of significantly rewired CRE combinations (p<0.01). (.pdf, 227 KB)
- Figure S1 - Phylogenetic screen identifies co-occurring CREs with spacing bias. (.pdf, 300 KB)
- Table S1 - CRE combinations and supporting experimental evidence. (.zip, 1,109 KB)
- Table S2 - List of TF pairs identified from 7 Genome search, and number of co-occurrences in promoters for each species. (.zip, 396 KB)
