# Supplementary material for: Combinatorial Cis-regulation in Saccharomyces Species
Source: G3 (Bethesda). 2016 Jan 12;6(3):653–67. doi: 10.1534/g3.115.024331 (PMC4777128; doi:10.1534/g3.115.024331)
Supplement: Supporting Information [file supp_g3.115.024331_FigureS2.pdf]

A.

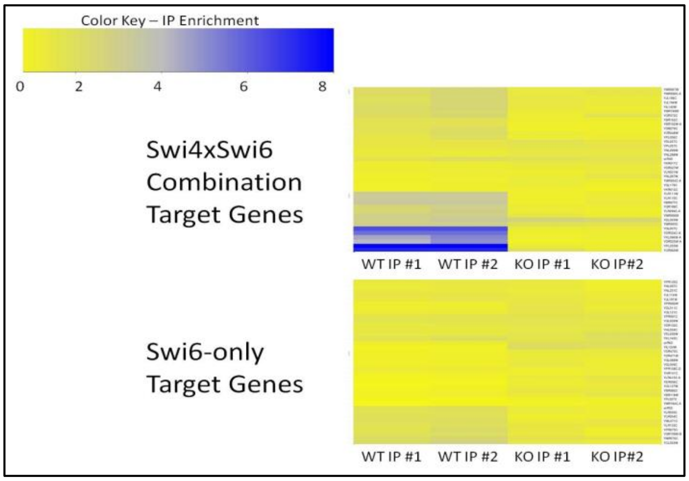

B.

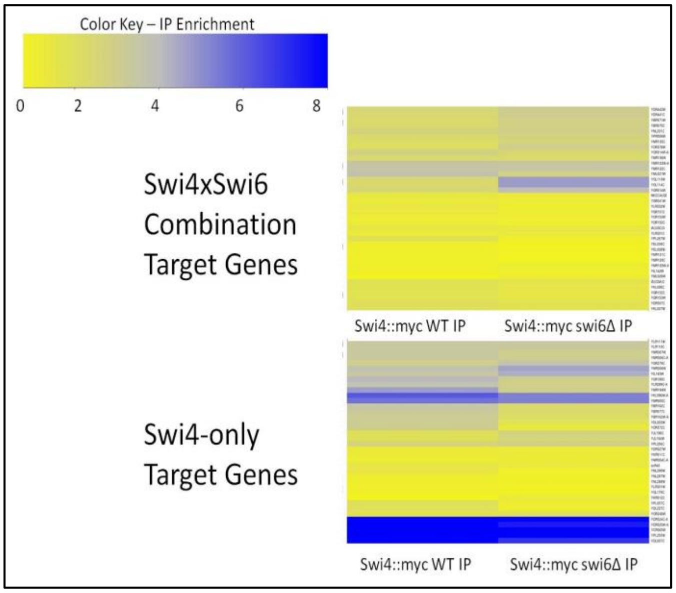

Figure S2 Enrichment ratios are depicted as a heat map for each replicate in *SWI6::myc* and *SWI6::myc/swi4Δ* ChIP experiments. Enrichment ratio is calculated as the normalized number of reads that aligned to promoter in the IP sample in ratio to normalized number of reads that aligned to that same promoter in the INPUT sample. The top heat map shows that Swi6 occupancy is significantly lower in the *swi4*Δ than the wild-type strain for Swi6 targets that are also bound by Swi4 in wild-type cells (p-value <2e-5). The lower heat map shows no significant trend in Swi6 occupancy for targets that are not bound by Swi4 in wild-type cells (p-value = 0.27). Enrichment ratios are depicted as a heat map for each replicate in *SWI4::myc* and *SWI4::myc/swi6Δ* ChIP experiments. The top heat map shows that Swi4 occupancy is generally independent of *SWI6* for Swi4 targets that are also bound by Swi6 in wild-type cells (p-value = 0.016). The lower heat map also shows no significant trend in Swi4 occupancy (p-value = 0.93).
