# Supplementary figures and images for: Combinatorial Cis-regulation in Saccharomyces Species
Source: G3 (Bethesda). 2016 Jan 12;6(3):653–67. doi: 10.1534/g3.115.024331 (PMC4777128; doi:10.1534/g3.115.024331)

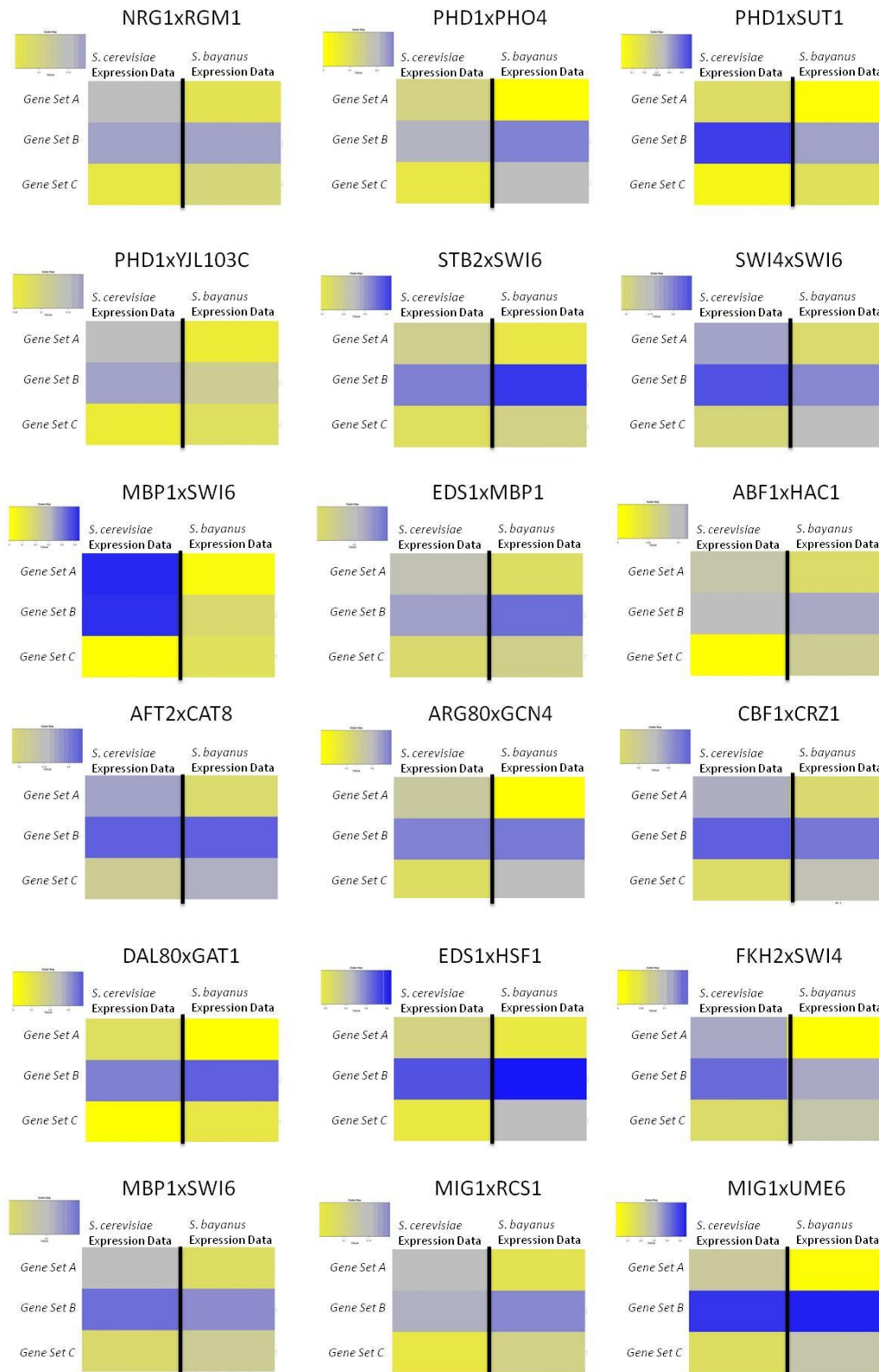

Figure S3. Heat Maps for the most definitive examples of CRE combination rewiring.

Supplement: Supporting Information [file supp_g3.115.024331_FigureS3.pdf]
