# Supplementary material for: Combinatorial Cis-regulation in Saccharomyces Species
Source: G3 (Bethesda). 2016 Jan 12;6(3):653–67. doi: 10.1534/g3.115.024331 (PMC4777128; doi:10.1534/g3.115.024331)
Supplement: Supporting Information [file supp_g3.115.024331_TableS3.pdf]

Table S3.

Figure 3 gene names (in order, top to bottom)

Top Panel

Ngr1 x Sut1 Combination Target Genes (UPPER)
YPR091C
YEL063C
YDR373W
YDR372C
YJR094W-A
YKR027W
YJL104W
YMR158W-B
YBR166C
YKL138C-A
YLR061W
YPR007C
tS(UGA)P
YHR055C
YPR092W
YPR008W
YMR160W
YFL037W
YPR072W
snR51
snR70
YBR167C

Nrg1-only Target Genes (LOWER)
YOR074C
YOR075W
YBR008C
YJR095W
YOR073W-A
RUF20
YFL004W
YPR190C
YBR009C
YEL003W
YBR010W
YBR058C-A
YFL026W
YFL027C
YDR056C
YDR057W
YDR039C
snR63
YBR058C
YPL189C-A
YPL189W

YMR319C

Bottom Panel

Nrg1 x Sut1 Combination Target Genes (UPPER)
YPR007C
YPR091C
tS(UGA)P
snR51
YDR373W
YDR372C
snR70
YFL037W
YKL138C-A
YLR061W
YPR008W
YMR158W-B
YJL104W
YKR027W
YBR167C
YPR092W
YPR072W
YMR160W
YHR055C
YEL063C
YJR094W-A
YBR166C

Sut1-only Target Genes (LOWER)
YOR216C
YJL200C
YJL144W
YHL034C
YPL056C
YMR193W
YPR156C
YNL117W
YGR270C-A
YIR024C
YER060W-A
YCR088W
YHL050W-A
YAL069W
YGR097W
YGR129W
YDR070C
YKR090W
YJL141C
YFR040W
YOR109W
YHR007C-A
