# Supplementary material for: Combinatorial Cis-regulation in Saccharomyces Species
Source: G3 (Bethesda). 2016 Jan 12;6(3):653–67. doi: 10.1534/g3.115.024331 (PMC4777128; doi:10.1534/g3.115.024331)
Supplement: Supporting Information [file supp_g3.115.024331_TableS4.pdf]

Table S4.

FIGURE 4 GENE NAMES (in order, top to bottom)

Top Panel

Gcn4 x Rtg3 Combination Target Genes (UPPER)

YNL142W
YNL143C
YDR051C
YJL136C
YJL135W
YHR060W
YGR161C
YPL021W
YNL187W
YMR159C
YMR160W
YJR090C
YMR158C-A
YPL179W
YPL127C
YNL078W
YMR075C-A
YDL053C
YJR094W-A
YFL039C
LSR1
YIL117C
YHR152W
YHR151C
YDL054C
YBR008C
YPL156C
YJL134W
YMR158W-B

Gcn4-only Target Genes (LOWER)
YDL170W
YDL171C
YPL273W
YLL027W
YGR193C
YMR175W-A
YNL228W
YHR063C
YOL058W
YMR174C
YNL174W

YOR282W
YLR169W
YHR098C
YKR014C
YPL260W
YPR008W
YPL250C
YBL049W
RPR1
YDR363W
YMR105C
YPR184W
YIL135C
tR(UCU)B
YDR135C
YMR172C-A
YJR151W-A
YDR378C

Bottom Panel

Gcn4 x Rtg3 Combination Target Genes (UPPER)
YJR094W-A
YJL134W
LSR1
YPL127C
YMR075C-A
YJR090C
YMR158C-A
YHR060W
YPL021W
YFL039C
YDL053C
YDL054C
YHR152W
YHR151C
YPL179W
YIL117C
YJL136C
YJL135W
YPL156C
YNL187W
YDR051C
YBR008C
YNL142W
YNL143C
YNL078W
YMR159C
YMR160W
YGR141C
YMR158W-B

Rtg3-only Target Genes (LOWER)
YHR056W-A
YDR366C
YLR458W
YML081W
YEL009C-A
YDR302W
YDR070C
YBL058W
YMR253C
YLP298C
YHR050W-A
YIL047C
YPR046W
tS(AGA)M
YGR024C
YPR194C
YER065C
YDR342C
YIR029W
YDR507C
YMR097C
YDR319C
YLR033W
YMR133W
YKR098C
YNL252C
YNL112W
YDR214W
YHR039C
