# Supplementary material for: Combinatorial Cis-regulation in Saccharomyces Species
Source: G3 (Bethesda). 2016 Jan 12;6(3):653–67. doi: 10.1534/g3.115.024331 (PMC4777128; doi:10.1534/g3.115.024331)
Supplement: Supporting Information [file supp_g3.115.024331_TableS5.pdf]

Table S5. 81 CRE pairs with significant (P<0.05) rewiring

Pair	P-value
ABF1xHAC1	0.0114
ABF1xRPN4	0.0264
ABF1xSTB1	0
AFT2xCAT8	0.011
AFT2xMIG1	0.0327
AFT2xZMS1	0.0037
ARG80xGCN4	0.0197
CAT8xUME6	0.037
CBF1xCRZ1	0.0013
CBF1xPHD1	0
CRZ1xSTP1	0.002
CRZ1xSUT1	0.037
CRZ1xTYE7	0.0112
DAL80xGAT1	0.0001
DAL80xGLN3	0.0036
DAL80xGZF3	0.0009
DAL82xPHD1	0.0015
EDS1xHSF1	0.0137
EDS1xMBP1	0.0016
EDS1xPBF2	0.0109
FKH1xSTB1	0.0036
FKH1xSWI4	0.0011
FKH1xSWI6	0.0013
FKH2xSTB1	0.0111
FKH2xSWI4	0
FKH2xSWI6	0.0013
GIS1xMIG3	0.0015
GIS1xNRG1	0.0001
GIS1xRDS2	0.0048
GIS1xSTP1	0.0166
GIS1xSUT1	0.0122
HAC1xSWI6	0.0022
HAP1xZMS1	0.0258
HAP2xRSC3	0.013
MBP1xREB1	0.0199
MBP1xSTB1	0.0315
MBP1xSWI6	0.0018
MET32xPHO4	0.0093
MIG1xPDR1	0.0001
MIG1xRCS1	0.0004
MIG1xRPH1	0.007
MIG1xSTP1	0
MIG1xSTP2	0
MIG1xUME6	0.002
MIG3xMSN4	0.0434
MIG3xPDR1	0
MIG3xRGM1	0.0011

MIG3xSTB5	0.0001
MIG3xSTP1	0.0004
MIG3xSTP2	0.0255
MIG3xUME6	0.0196
MIG3xYER130C	0.0045
MIG3xYPL230W	0.002
MIG3xZMS1	0.0014
MSN2xYDR026C	0.0197
MSN2xYJL103C	0.0161
NRG1xRGM1	0
NRG1xYPL230W	0.0004
NRG1xZMS1	0
PBF1xSTB3	0.0012
PHD1xPHO4	0
PHD1xSUT1	0.0016
PHD1xYER184C	0.0022
PHD1xYJL103C	0.0026
RDS2xRGM1	0.0047
RDS2xYPL230W	0.0044
REB1xSTB3	0.0024
RGM1xSTP1	0.0181
RGM1xSUT1	0.0153
RPH1xZMS1	0.0002
RPN4xYDR026C	0.0303
STB2xSWI6	0.001
STB3xYBL054W	0.0003
STP1xYER184C	0.0045
STP1xYJL103C	0.0107
STP1xYPL230W	0.0161
SUT1xUME6	0.0012
SUT1xYER184C	0.0004
SUT1xYJL103C	0.0001
SUT1xYPL230W	0.0121
SWI4xSWI6	0.0017
