# Supplementary material for: Combinatorial Cis-regulation in Saccharomyces Species
Source: G3 (Bethesda). 2016 Jan 12;6(3):653–67. doi: 10.1534/g3.115.024331 (PMC4777128; doi:10.1534/g3.115.024331)
Supplement: Supporting Information [file supp_g3.115.024331_TableS6.pdf]

Table S6: GO pathway enrichment of significantly rewired CRE combinations (p<0.01)

CRE Pair	*S. cerevisiae* Pathway	Conserved Pathway	*S. bayanus* Pathway
ABF1x HAC1	poly(A)+ mRNA export from nucleus	protein O-linked glycosylation	isoprenoid biosynthetic process
ABF1x RPN4		proteasomal ubiquitin-dependent protein catabolic process	chromatin silencing
ABF1x STB1		spindle pole body separation	mitotic spindle organization in nucleus
AFT2x CAT8	nitrogen catabolite activation of transcription		activation of Rab GTPase activity
AFT2x MIG1	histone demethylation	oxidation-reduction process	positive regulation of translational elongation
AFT2x ZMS1	histone demethylation	positive regulation of translational elongation	leukotriene biosynthetic process
ARG80x GCN4	negative regulation of actin filament polymerization	arginine biosynthetic process	lysine biosynthetic process
CAT8x UME6	phenylalanyl-tRNA aminoacylation	ATP synthesis coupled proton transport	regulation of ergosterol biosynthetic process
CBF1x CRZ1	cysteine biosynthetic process via cystathionine	glutathione biosynthetic process	ammonium transport
CBF1x PHD1	regulation of nitrogen utilization	ER-associated protein catabolic process	positive regulation of translational elongation
CRZ1x STP1	thiamine transport		uridine transport
CRZ1x SUT1	nitrogen catabolite activation of transcription	tricarboxylic acid cycle	cellular response to hypoxia
CRZ1x TYE7	cysteine biosynthetic process via cystathionine		telomere maintenance via telomerase
DAL80xGAT1	gamma-aminobutyric acid transport	nitrogen catabolite repression of transcription	leucine biosynthetic process
DAL80xGLN3	gamma-aminobutyric acid transport	nitrogen catabolite repression of transcription	leucine biosynthetic process
DAL80xGZF3	gamma-aminobutyric acid transport	nitrogen catabolite repression of transcription	adenine catabolic process
DAL82xPHD1	rRNA pseudouridine synthesis		lagging strand elongation
EDS1x MBP1	positive regulation of histone acetylation	tRNA-type intron splice site recognition and cleavage	glycolysis
EDS1x PBF2	box H/ACA snoRNP assembly	ribosomal large subunit biogenesis	rRNA processing
FKH1x STB1	premeiotic DNA replication		cellular response to heat
FKH1x SWI4	premeiotic DNA replication	mitotic chromosome condensation	telomere capping
FKH1x SWI6	premeiotic DNA replication	mitotic chromosome condensation	telomere capping

FKH2x STB1	premeiotic DNA replication	mitotic chromosome condensation	telomere capping
FKH2x SWI4	premeiotic DNA replication	mitotic chromosome condensation	telomere capping
FKH2x SWI6	premeiotic DNA replication	mitotic chromosome condensation	telomere capping
GIS1x MIG3	protein monoubiquitination	glycogen biosynthetic process	energy reserve metabolic process
GIS1x RDS2	regulation of translational fidelity		negative regulation of sphingolipid biosynthetic process
GIS1x STP1	uridine transport	TOR signaling cascade	regulation of fungal-type cell wall 1,3-alpha-glucan biosynthetic process
GIS1x SUT1	uridine transport	glycogen biosynthetic process	thiamine transport
HAC1x SWI6	proline biosynthetic process	dolichol-linked oligosaccharide biosynthetic process	protein import into peroxisome matrix, receptor recycling
HAP2x RSC3	mitochondrial magnesium ion transport	heme transport	uridine transport
MBP1x REB1	tryptophan biosynthetic process	chitin biosynthetic process	nucleotide-sugar transport
MBP1x STB1	regulation of establishment or maintenance of cell polarity	negative regulation of ubiquitin-protein ligase activity involved in mitotic cell cycle	DNA repair
MBP1x SWI6	post-Golgi vesicle-mediated transport	negative regulation of ubiquitin-protein ligase activity involved in mitotic cell cycle	GDP-mannose biosynthetic process
MET32xPHO4	threonine biosynthetic process	glutathione biosynthetic process	drug transmembrane transport
MIG1x PDR1	disaccharide catabolic process	sporulation resulting in formation of a cellular spore	glycolysis
MIG1x RCS1	histone demethylation	nucleotide-excision repair, DNA incision, 3'-to lesion	
MIG1x STP1	cellular respiration	acetyl-CoA catabolic process	
MIG1x STP2	cellular respiration		inositol biosynthetic process
MIG1x UME6	vacuole inheritance	acetyl-CoA catabolic process	hydrogen peroxide catabolic process
MIG3x MSN4	UDP-glucose metabolic process	glycogen biosynthetic process	energy reserve metabolic process
MIG3x RGM1	protein monoubiquitination	glycogen biosynthetic process	energy reserve metabolic process
MIG3x STB5	glycosphingolipid biosynthetic process		activation of Rab GTPase activity
MIG3x STP1	cellular respiration	acetyl-CoA catabolic process	
MIG3x STP2	cellular respiration		inositol biosynthetic process
MIG3x YPL230W	protein monoubiquitination	glycogen biosynthetic process	energy reserve metabolic process

MIG3x ZMS1	protein monoubiquitination	glycogen biosynthetic process	
MSN2x YDR026C	oxidation-reduction process	early endosome to Golgi transport	cell shape checkpoint
MSN2x YJL103C	proteasome regulatory particle assembly	tricarboxylic acid cycle	phosphatidylinositol biosynthetic process
PHD1x PHO4	phosphate transport	tricarboxylic acid cycle	positive regulation of translational elongation
PHD1x SUT1	diadenosine polyphosphate catabolic process	mitochondrial electron transport, ubiquinol to cytochrome c	mitochondrial fusion
PHD1x YER184C	fatty acid oxidation		glycogen biosynthetic process
PHD1x YJL103C	fatty acid oxidation		ammonium transport
RDS2x YPL230 W	regulation of translational fidelity		negative regulation of sphingolipid biosynthetic process
REB1x STB3	translational termination	regulation of translational initiation	rRNA processing
RGM1x STP1	uridine transport	TOR signaling cascade	regulation of fungal-type cell wall 1,3-alpha-glucan biosynthetic process
RGM1x SUT1	uridine transport	glycogen biosynthetic process	thiamine transport
RPH1x ZMS1	pentose-phosphate shunt, oxidative branch		proteasome regulatory particle assembly
RPN4x YDR026C	ATP synthesis coupled proton transport		negative regulation of transcription from RNA polymerase II promoter in response to iron
STB2x SWI6	septin checkpoint	DNA replication	dolichol-linked oligosaccharide biosynthetic process
STP1x YER184C	hydrogen peroxide catabolic process	cellular respiration	ATP-dependent chromatin remodeling
STP1x YJL103C	hydrogen peroxide catabolic process	cellular respiration	leucine biosynthetic process
STP1x YPL230 W	uridine transport	TOR signaling cascade	regulation of fungal-type cell wall 1,3-alpha-glucan biosynthetic process
SUT1x UME6	vacuole inheritance	ATP synthesis coupled proton transport	phosphate transport
SUT1xYE R184C	positive regulation of cell cycle	tricarboxylic acid cycle	plasmid maintenance
SUT1xYJ L103C	positive regulation of cell cycle	tricarboxylic acid cycle	
SUT1x YPL230 W	uridine transport	glycogen biosynthetic process	thiamine transport
SWI4x SWI6	regulation of establishment or maintenance of cell polarity	negative regulation of ubiquitin-protein ligase activity involved in mitotic cell cycle	
