# Supplementary material for: Combinatorial Cis-regulation in Saccharomyces Species
Source: G3 (Bethesda). 2016 Jan 12;6(3):653–67. doi: 10.1534/g3.115.024331 (PMC4777128; doi:10.1534/g3.115.024331)
Supplement: Supporting Information [file supp_g3.115.024331_FigureS1.pdf]

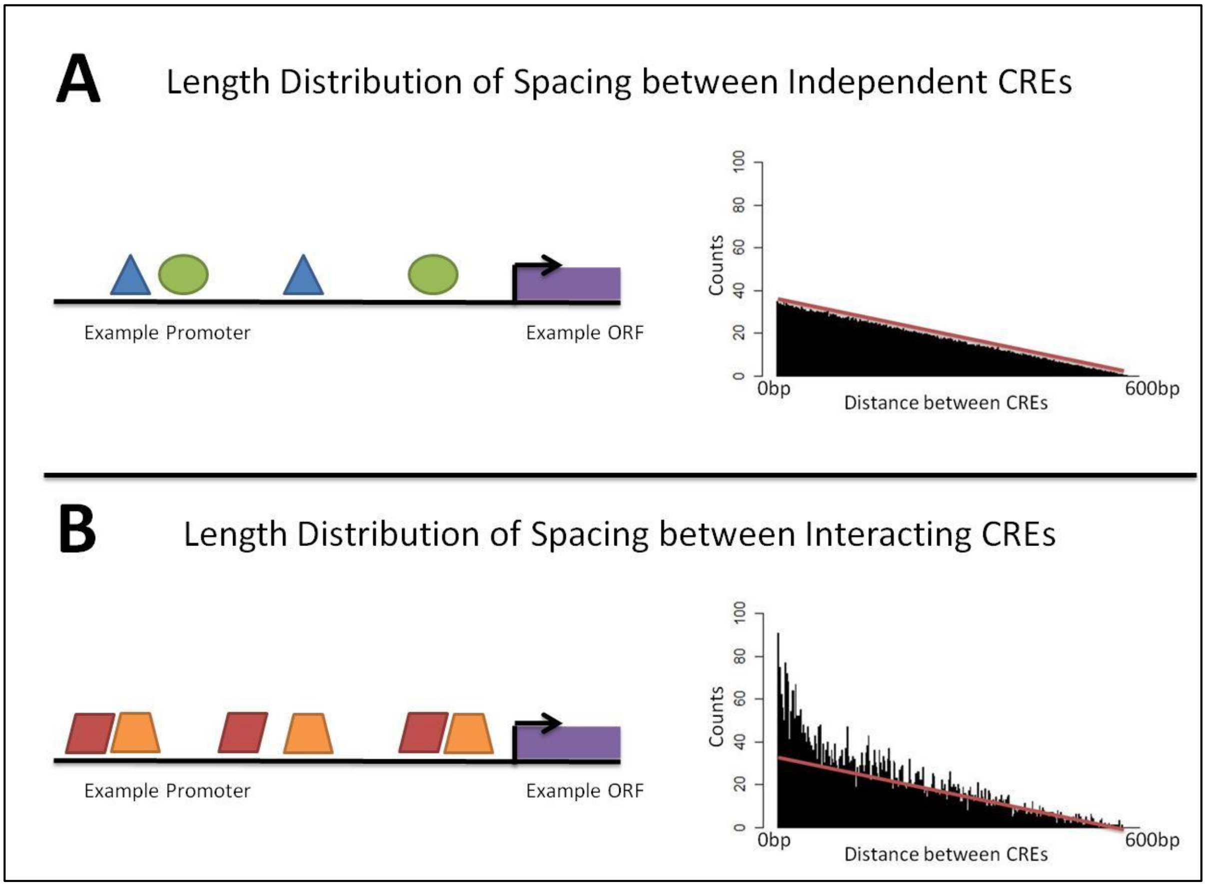


**Figure S1. Phylogenetic screen identifies co-occurring CREs with spacing bias. A)** If CREs are independent then there is uniform probability of any particular distance separating the two CREs. However, there are a greater number of chances to observe shorter distances than longer distances. Therefore, as depicted in the histogram, the expected number of observations decreases linearly as the distance between CREs increases. **B)** If CREs cluster near each other, then this non-random distribution is observed in addition to the random expectation. This results in a deviation from the random expectation at short distances.
