# Supplementary material for: Combinatorial Cis-regulation in Saccharomyces Species
Source: G3 (Bethesda). 2016 Jan 12;6(3):653–67. doi: 10.1534/g3.115.024331 (PMC4777128; doi:10.1534/g3.115.024331)
Supplement: Supporting Information [file supp_g3.115.024331_TableS1.zip › TableS1.pdf]

Table S1. CRE combinations and supporting experimental evidence. Literature reference provided for datasets that indicate significant interaction between CREs (p-value <0.01). 'Ref' column provides reference for known interactions mentioned in the main text. References listed here are provided at the end of the Table. (Data also available in TableS1.xls)

Pair	ChIP	Path	Expression	Orient	Ref
ABF1xCBF1	(27) (26)	(35)	(31) (32)		
ABF1xUME6	(27)	(34)	(32)		
AFT2xRAP1	(27)	(34) (35)	(29)		
AFT2xRCS1	(27)	(34) (35)	(31) (32)	(29)	
AFT2xSFP1	(27)	(34) (35)	(29) (30)	(30)	
ARG80xBAS1	(27) (26)	(34) (35)	(29) (30) (31) (32)		
ARG80xGCN4	(27) (26)	(34) (35)	(29) (30) (31)		(49)
ARG80xSUT1	(27)	(34) (35)	(29) (31) (32)		
ARG81xBAS1	(27) (26)	(34)	(30) (31)		
ARG81xGCN4	(27) (26)	(34)	(29)	(29)	
BAS1xGCN4	(27) (26) (28)	(34) (35)	(29) (32)		(50)
BAS1xRTG3	(27) (26)	(34) (35)	(29) (31) (32)		
CAD1xYAP7	(27)	(34)	(30)		
CBF1xGCN4	(27)	(34) (35)	(29) (30) (31) (32)		
CBF1xINO2	(27) (26)	(34)	(31)	(30)	
CBF1xINO4	(27) (26)	(34) (35)	(31)		
CBF1xMET32	(27)	(34) (35)	(29) (30) (31)		(58)
CBF1xMET4	(27) (26)	(34) (35)	(29) (30) (31)		
CIN5xMIG3	(27)	(35)	(30) (31)		
CUP9xRAP1	(27) (26)	(35)	(32)		
DAL82xUME6	(27)	(34)	(29)		
DIG1xSTE12	(27) (26) (28)	(34)	(29) (31)		(44)
EDS1xHSF1	(27)	(34)	(31)	(29)	
FHL1xRAP1	(27) (26) (28)	(34) (35)	(29) (30)		(42)
FHL1xRPH1	(27) (26) (28)	(35)	(29)		
FHL1xSFP1	(27) (26) (28)	(34) (35)	(29) (30) (31)	(30) (29)	(43)
FKH1xFKH2	(27) (26) (28)	(34)	(32)		
FKH1xMBP1	(27) (26)	(34)	(29) (30) (31) (32)		(48)
FKH1xMCM1	(27) (26)	(35)	(30)		(56)
FKH1xSWI6	(27) (26)	(34)	(29) (30) (31)	(29)	(47)
FKH2xMBP1	(27) (26)	(34) (35)	(29) (30) (31)		
FKH2xMCM1	(27) (26)	(35)	(30)		(56)

FKH2xSTB1	(27) (26)	(34)	(29) (30) (31)	(30) (29)	
FKH2xSWI6	(27) (26)	(34)	(29) (30) (31)	(29)	
FZF1xSPT23	(27)	(34)	(29) (32)	(30) (29)	
GAL4xSTP1	(28)	(34) (35)	(32)		
GCN4xLEU3	(27) (26) (28)	(34) (35)	(30) (31) (32)		
GCN4xRTG3	(27) (26)	(34) (35)	(29) (32)	(30) (29)	
GCR1xGCR2	(27) (26)	(34)	(32)		(60)
GCR1xRAP1	(27) (26) (28)	(34) (35)	(30) (31) (32)		(60)
GCR2xTHI2	(27) (26)	(34) (35)	(31) (32)		
GLN3xGZF3	(27)	(35)	(32)		
HAP1xPDR1	(27)	(35)	(29) (31)		
HAP1xSUT1	(27)	(34)	(29)		
HAP3xHAP4	(27) (26)	(34) (35)	(30) (31)		
HAP3xUGA3	(26)	(35)	(32)		
HAP4xHAP5	(27) (26)	(34) (35)	(30) (31)		
HMS1xTYE7	(27)	(34)	(31) (32)		
HSF1xXBP1	(27)	(34)	(29) (30) (31)		
INO2xINO4	(27) (26) (28)	(34) (35)	(32)		(57)
LEU3xSKN7	(27) (28)	(35)	(32)		
LEU3xUME6	(28)	(34) (35)	(31) (32)		
MBP1xNDD1	(27) (26)	(35)	(29) (31)		
MBP1xSTB1	(27) (26)	(34) (35)	(29) (30) (31) (32)	(30)	(40)
MBP1xSWI4	(27) (26)	(34) (35)	(29) (30) (31) (32)	(30)	(39)
MBP1xSWI6	(27) (26)	(34) (35)	(29) (30) (31) (32)		(39)
MCM1xSTE12	(27) (26)	(34)	(29) (30) (31)		
MCM1xSWI4	(27) (26)	(34)	(29) (30) (31) (32)		
MCM1xSWI6	(27) (26)	(34) (35)	(29) (30) (31) (32)		
MET32xMET4	(27)	(35)	(29) (30) (31)		
MET32xTYE7	(27)	(34) (35)	(29) (30) (31)	(30)	
MIG1xMIG3	(27)	(34) (35)	(29) (30) (31)	(30)	
MIG1xPHD1	(27) (26)	(34) (35)	(30) (31)		
MIG1xRGT1	(27) (26)	(35)	(31)	(30)	(51)
MIG1xSKN7	(27) (26)	(35)	(30) (31)		
MIG1xSOK2	(27) (26)	(35)	(31) (32)		
MIG1xSUT1	(27)	(34) (35)	(29) (30) (31) (32)	(30)	
MIG2xMOT3	(27)	(35)	(31)		
MIG2xOAF1	(27)	(35)	(30) (31)		
MIG3xMSN4	(27)	(34) (35)	(29) (30) (31)	(30) (29)	
MIG3xNRG1	(27)	(34) (35)	(30) (31)	(30)	
MIG3xPHD1	(27)	(35)	(29) (31)		
MIG3xRGT1	(27)	(35)	(29) (30) (31)		

MIG3xSTP1	(27)	(34) (35)	(29) (30) (31)	(29)	
MIG3xSUT1	(27)	(34) (35)	(29) (30) (31)	(30)	
MIG3xYPR196W	(27)	(35)	(29) (30) (31)		
MOT3xSKN7	(27)	(34)	(29) (32)		
MSN2xPHD1	(26)	(34)	(29) (30) (31)		
MSN2xSKN7	(27) (28)	(35)	(30) (31)		
MSN2xZMS1	(28)	(35)	(29) (30) (31) (32)	(30) (29)	
MSN4xSKN7	(27)	(35)	(30) (31) (32)		
MSN4xXBP1	(27)	(35)	(29) (30) (31) (32)		
NDD1xSTB1	(27) (26)	(34)	(29)		
NDD1xSWI6	(27) (26)	(34)	(29) (31)		
NHP6AxPHO2	(28)	(34) (35)	(29)		
NRG1xPDR1	(27) (26)	(35)	(30)		
NRG1xRIM101	(27) (26)	(35)	(30) (31) (32)	(29)	
OAF1xPDR3	(27)	(35)	(30) (31) (32)		(52)
PDR1xZMS1	(27) (26)	(34)	(29) (30) (31)		
PDR3xSTP1	(27)	(34)	(29) (30) (31)		
PHD1xSUT1	(27)	(34) (35)	(31) (32)	(30)	
RAP1xSFP1	(27) (26) (28)	(34) (35)	(29) (30) (31) (32)		
RAP1xSTP1	(28)	(35)	(32)		
RCS1xSFP1	(27) (26)	(34) (35)	(29) (32)		
RDS1xSTP1	(27)	(35)	(30) (31)		
RGM1xRPH1	(27) (26)	(35)	(29) (30) (31) (32)	(30)	
RGT1xSUT1	(27)	(35)	(30) (31) (32)	(29)	
RPH1xZMS1	(28)	(34) (35)	(29) (30) (31) (32)	(30)	
RTG3xSTB5	(27)	(34)	(29) (32)	(30) (29)	
SKN7xUGA3	(28)	(35)	(29) (31)		
SKN7xZMS1	(28)	(35)	(31)		
SNF1xYBL054W	(27)	(35)	(32)		
STB1xSWI4	(27) (26)	(34)	(29) (30) (32)	(29)	(41)
STB1xSWI6	(27) (26)	(34) (35)	(29) (30) (31)	(30) (29)	(41)
STP1xSTP2	(27) (26)	(35)	(30) (31) (32)		
STP1xTHI2	(27) (26)	(34) (35)	(29) (32)		
STP1xZMS1	(28)	(35)	(29) (30) (31) (32)		
SWI4xSWI6	(27) (26)	(34) (35)	(29) (30) (31) (32)	(30) (29)	(39)
UME6xZMS1	(28)	(34)	(31)		
ABF1xACA1		(34)	(32)		
ABF1xBAS1		(34)	(29) (30) (31) (32)		
ABF1xCST6		(35)	(32)		
ABF1xGCN4		(34) (35)	(29) (30) (31)		
ABF1xHAP5		(34) (35)	(30) (32)		

ABF1xHSF1	(27) (26)		(30) (32)		
ABF1xINO2	(27) (26)	(34)			
ABF1xINO4	(27) (26)	(34)			
ABF1xPHD1		(34)	(29) (32)		
ABF1xRPN4		(34) (35)	(29) (30) (31) (32)		
ABF1xSTB3		(34)	(29) (30) (31)	(30) (29)	
ABF1xSWI4		(34)	(30) (31) (32)		
ABF1xTBF1		(34) (35)	(29)		
ABF1xYPR196W		(34)	(29)		
ACA1xGCN4		(34)	(29) (32)	(30) (29)	
ACA1xPHD1		(34)	(32)		
ACA1xRPN4		(34) (35)	(30) (31) (32)		
ACA1xRTG3		(34)	(30) (32)		
ACA1xSKN7		(35)	(32)	(30) (29)	
ACA1xSTP1		(34)	(30) (31) (32)		
ACE2xSKN7	(27) (26)		(32)		
ACE2xSWI5	(27) (26)		(29) (32)		(53)
AFT2xCBF1		(35)	(31) (32)		
AFT2xFHL1		(34) (35)	(29)	(30) (29)	
AFT2xMIG1	(27)		(29) (31) (32)		
AFT2xRPH1	(27)		(32)		
AFT2xRPN4	(27)		(32)		
AFT2xTYE7		(35)	(31)		
AFT2xYJL103C		(35)	(32)		
ARG80xMIG1		(34)	(31)		
ARG80xMIG3	(27)		(29) (30) (31)		
ARG81xMIG3	(27)		(31)		
ARG81xPHO4		(34)	(31)		
ARG81xRTG3	(27) (26)		(29) (30)		
ARG81xSKN7		(34)	(31)		
ARG81xSTP1	(27) (26)		(29) (30) (31)		
ARO80xCST6	(27)		(32)		
ARO80xPDR1	(27) (26)		(29)		
ARO80xPDR3	(27)		(32)		
ARO80xSTP1	(27) (26)		(30)		
ASH1xMBP1	(27) (26)		(32)		
ASH1xNDD1	(27) (26)		(29) (30)		
ASH1xSTP1	(28)		(32)		
ASH1xSWI6	(27) (26)		(32)		
BAS1xSTP3		(34) (35)	(29) (31)		
CAT8xGAL4		(35)	(32)		

CAT8xGIS1		(35)	(30) (31)		
CAT8xMIG1		(35)	(30) (31)		
CAT8xMIG3		(34)	(29) (30) (31)		
CAT8xMSN2		(35)	(29) (30) (31)		
CAT8xMSN4		(35)	(29) (30) (31)		
CAT8xPDR1		(35)	(30) (31)		
CAT8xPHD1		(35)	(30) (31) (32)	(29)	
CAT8xRDS1		(35)	(31)		
CAT8xRGM1		(35)	(30) (31)		
CAT8xRPH1		(34) (35)	(30) (31)		
CAT8xRSC3		(35)	(30) (31)	(30)	
CAT8xRTG3		(35)	(31) (32)		
CAT8xSTP1		(35)	(29) (30) (31)		
CAT8xSUT1		(35)	(30) (31)		
CAT8xSWI5		(35)	(29) (32)	(30)	
CAT8xUME6		(35)	(30) (31) (32)		
CAT8xYER184C		(35)	(30) (31)		
CAT8xYPL230W		(35)	(30) (31)		
CBF1xDAL81	(27)		(32)		
CBF1xGAT1		(34) (35)	(31) (32)		
CBF1xHAC1		(35)	(31)		
CBF1xHAP5	(27)	(35)			
CBF1xMATALPHA2		(34) (35)	(31)		
CBF1xMET28		(34)	(32)		(58)
CBF1xOAF1		(35)	(32)		
CBF1xPDR8		(35)	(32)		
CBF1xPHD1		(34)	(29) (30) (31)		
CBF1xPHO4		(34) (35)	(31) (32)		
CBF1xRAP1		(35)	(32)		
CBF1xRTG3		(34) (35)	(29) (31) (32)		
CBF1xSUT1		(34) (35)	(31) (32)		
CBF1xTYE7	(27)	(34) (35)			
CBF1xYER184C		(34) (35)	(32)		
CHA4xRGM1		(34) (35)	(32)		
CIN5xNRG1	(27) (26)		(31)		
CIN5xPDR3		(35)	(30) (31)		
CIN5xRGT1	(27) (26)		(31) (32)		
CIN5xXBP1	(27)		(30)		
CRZ1xYJL103C		(35)	(29) (30) (31)		
CST6xSUT1		(35)	(29) (30) (31)		

CST6xSWI6		(34)	(32)		
CST6xYER184C		(35)	(31) (32)	(29)	
CST6xYJL103C		(35)	(31) (32)		
CUP9xSFP1		(35)	(29) (30) (32)		
CUP9xSTP1	(27) (26)		(31) (32)		
DAL80xGLN3		(35)	(30)		
DAL80xGZF3	(27)		(30) (32)		
DAL80xHAP1		(35)	(29)		
DAL82xRDS1		(34)	(29) (30)		
DAL82xSTP1	(27)		(29) (30) (31)	(30) (29)	
EDS1xPBF1		(34)	(29) (30)	(30) (29)	
EDS1xPBF2		(34)	(29) (30) (31)		
EDS1xSTB3		(34)	(30)	(30)	
FHL1xMET31	(27) (26)	(35)			
FHL1xMIG1		(35)	(29)		
FHL1xMIG3		(35)	(29)		
FHL1xPHO4		(35)	(29)		
FHL1xRCS1		(35)	(29)	(29)	
FHL1xRSC3		(35)	(29)		
FKH1xSTB1		(34)	(29) (30) (31)	(29)	
FKH1xSWI4	(27) (26) (28)		(29) (30) (31) (32)	(29)	(47)
FKH2xHCM1		(34)	(32)		
FKH2xSWI4	(27) (26) (28)		(29) (30) (31) (32)	(29)	
GAL4xGAL80	(27)	(34) (35)			
GAL4xPDR1	(27) (26)	(35)			
GAL80xMIG3	(27)	(34) (35)			
GAL80xSTP1	(27)		(32)		
GAL80xSUT1		(34)	(32)		
GAT1xGLN3	(27)	(35)		(29)	
GAT1xGZF3	(27)		(32)		
GAT1xSIP4	(27)		(32)		
GAT4xGCR1		(34)	(31)		
GAT4xLYS14		(34)	(29)		
GCN4xSTB3		(34) (35)	(29) (30)		
GCN4xTYE7		(34) (35)	(29) (30) (31) (32)		
GCR1xSTP2	(27) (26)	(34)			
GIS1xMIG1		(34)	(29) (30) (31)	(30) (29)	
GIS1xMIG3		(34) (35)	(29) (30) (31)	(29)	
GIS1xNHP10		(34)	(30) (31)		
GIS1xRPH1		(35)	(29) (30) (31)	(30)	
GIS1xSUT1		(34) (35)	(29) (30) (31)	(29)	

GIS1xYER184C		(34) (35)	(30) (31)	(30)	
GIS1xYJL103C		(35)	(30) (31)	(30)	
GSM1xMIG3		(34)	(30) (31)		
GSM1xSUT1		(34)	(30) (31)		
HAC1xPHO4		(35)	(31)		
HAC1xYLL054C		(35)	(31)		
HAP1xMIG1		(34)	(29) (31)	(30)	
HAP1xPHO4		(34) (35)	(31)		
HAP1xRDS1		(35)	(31)		
HAP1xSKN7	(27)	(35)			
HAP1xSOK2		(35)	(31)		
HAP1xTOS8	(27)	(35)			
HAP1xYER184C		(34) (35)	(29)		
HAP1xYJL103C		(34)	(30)		
HAP2xSUT1		(34) (35)	(30) (31)		
HAP3xMBP1		(34) (35)	(30) (31)		
HAP3xMIG3	(27)	(34) (35)			
HAP3xPDR8		(34) (35)	(32)		
HAP3xRDS1	(27)	(35)			
HAP3xSKN7		(35)	(32)		
HAP3xSUT1		(34) (35)	(29) (31)		
HAP3xZMS1		(34) (35)	(29) (32)		
HAP4xRDS1	(27)		(30) (31)	(30)	
HAP4xSKN7		(34) (35)	(32)		
HAP4xSUT1		(34) (35)	(30) (31) (32)	(30) (29)	
HAP5xNHP10		(34) (35)	(30)		
HAP5xRDS1		(34)	(31)	(29)	
HAP5xSKN7		(34) (35)	(30) (31) (32)		
HAP5xSUT1		(34) (35)	(30) (31)		
HMRA2xMCM1		(34) (35)	(29)		
HMS1xMIG3	(27)		(29) (31)		
HMS1xNHP10		(34)	(31)		
HMS1xSOK2	(26)		(32)		
HMS1xSUT1	(27)		(30) (31)	(30)	
IME1xPDR3		(35)	(31)		
IME1xRDS1	(27)		(30)		
IME1xSUT1		(35)	(30) (31) (32)	(30)	
IME1xUME6		(34)	(30)		(46)
IME1xYPR196W		(35)	(30) (31)		
INO2xMATALPHA2		(35)	(30)		

INO2xPDR3		(34)	(30)		
INO2xRAP1	(27) (26) (28)	(35)			
INO2xTYE7		(34) (35)	(31)		
INO2xYAP7		(34)	(29) (30)		
INO4xTYE7		(34) (35)	(29) (31)		
INO4xYAP7		(34)	(29) (30) (31)		
LEU3xSTP2	(27)		(32)		
LEU3xSUT1	(27)		(29) (31) (32)		
LEU3xSWI4	(28)		(29) (31) (32)		
LYS14xMIG1		(35)	(29) (30) (31)		
LYS14xPDR3		(35)	(30) (31)		
LYS14xSTB5		(34)	(29) (31)		
LYS14xSTE12		(34)	(31)	(29)	
LYS14xYJL103C		(34)	(29)		
MATALPHA2xMCM1		(34)	(31)		(54)
MATALPHA2xNHP6B		(34)	(29)		
MATALPHA2xSTE12		(34)	(29)	(29)	
MBP1xMCM1	(27) (26)		(29) (31)	(30)	
MBP1xMET28		(34)	(29)		
MBP1xPHD1	(27) (26)		(31)		
MBP1xREB1		(34)	(30)		
MBP1xRTG3	(27)		(29)		
MBP1xSTB2		(34) (35)	(29) (30) (31) (32)	(30) (29)	
MBP1xUME6	(27)	(34)			
MBP1xYDR026C		(34)	(29) (30) (31) (32)		
MCM1xNDD1	(27) (26)		(29) (30) (31)		(56)
MCM1xSTP1	(27) (26)		(32)		
MCM1xYOX1	(27)		(29) (30) (31) (32)		(55)
MET28xSTB1		(34)	(29) (30) (31) (32)	(29)	
MET28xSUM1		(34)	(30) (32)		
MET28xSWI4		(34)	(29) (31) (32)		
MET28xSWI6		(34)	(29) (30)		
MET31xMIG3	(27)		(30) (31)		
MET31xPDR1	(27) (26)		(29)		
MET31xRPN4	(27)	(34) (35)		(30)	
MET32xPHO4		(34) (35)	(29) (30)		
MET4xRDS1		(35)	(30)		
MET4xTYE7		(34) (35)	(29) (30) (31)		
MIG1xMSN2		(34) (35)	(29) (30) (31) (32)	(29)	

MIG1xMSN4		(34) (35)	(29) (30) (31) (32)	(29)	
MIG1xNRG1	(27) (26)		(29) (30) (31) (32)		
MIG1xPDR1		(34)	(29) (30) (31)		
MIG1xPHO4		(34) (35)	(29) (31) (32)		
MIG1xRCS1	(26)		(29) (31) (32)		
MIG1xRDS1		(35)	(30) (31) (32)		
MIG1xRGM1		(34)	(29) (30) (31) (32)	(30) (29)	
MIG1xSFP1		(34) (35)	(32)		
MIG1xSTP1		(35)	(29) (30) (31) (32)		
MIG1xSTP2		(35)	(29) (30) (31)		
MIG1xUGA3	(27) (26)		(31) (32)		
MIG1xYER184C		(34)	(29) (30) (31) (32)		
MIG1xYJL103C		(34) (35)	(30) (31)		
MIG1xYPL230W		(34)	(29) (30) (31)	(30) (29)	
MIG1xZMS1		(34)	(29) (30) (31) (32)		
MIG2xSOK2	(27)		(32)		
MIG3xMSN2		(34) (35)	(29) (30) (31)	(30) (29)	
MIG3xPDR1		(34) (35)	(29) (30) (31)		
MIG3xPDR8		(34) (35)	(29) (30) (31)		
MIG3xRDS1		(34)	(30) (31)		
MIG3xRGM1		(34) (35)	(29) (30) (31)	(29)	
MIG3xSOK2	(27)	(35)			
MIG3xSTB5	(27)		(29) (30) (31)		
MIG3xSTP2	(27)		(29) (30) (31)		
MIG3xYER184C		(34) (35)	(30) (31)		
MIG3xYJL103C		(34) (35)	(30) (31)		
MIG3xYPL230W		(34) (35)	(29) (30) (31)	(29)	
MIG3xZMS1		(34)	(29) (30) (31)	(30) (29)	
MSN2xNDD1	(27) (26)		(31)		
MSN2xPHO4		(35)	(31)	(30)	
MSN2xRPH1	(27) (28)		(29) (30) (31) (32)		
MSN2xSUT1		(34) (35)	(29) (30) (31) (32)	(30) (29)	
MSN2xYJL103C		(35)	(29) (30) (31) (32)		
MSN4xNDD1	(27) (26)		(31)		
MSN4xNRG1	(27)		(29) (30) (31)		
MSN4xPHD1	(27) (26)		(29) (30) (31)		
MSN4xRGM1	(27) (26)	(35)		(30) (29)	
MSN4xRPH1	(27)		(29) (30) (31) (32)	(30)	
MSN4xSUT1		(34) (35)	(29) (30) (31)	(30)	
MSN4xUGA3	(27)		(29) (30) (31) (32)	(30) (29)	
MSN4xYER184C		(34) (35)	(29) (30) (31)		

MSN4xYJL103C		(34) (35)	(29) (30) (31)		
MSN4xZMS1		(35)	(29) (30) (31)	(30) (29)	
NDD1xRDR1		(34)	(30) (31)		
NDD1xSUT1	(27)		(31)		
NDD1xSWI4	(27) (26)		(29) (31)		
NDD1xXBP1	(27)		(29)		
NHP10xNRG1		(34)	(29) (30) (31)		
NHP10xPDR1		(34)	(29) (30) (31)		
NHP10xPHD1		(34)	(30) (31)		
NHP10xRGM1		(34)	(30) (31)		
NHP10xSUT1		(34)	(29) (31)	(30)	
NHP10xYER184C		(34) (35)	(29) (30) (31)	(29)	
NHP10xYJL103C		(34)	(29) (30) (31)		
NHP10xYPL230W		(34)	(30) (31)		
NHP6AxSTB5		(34)	(29) (31)	(30) (29)	
NHP6BxSTB5		(34)	(31)	(29)	
NRG1xPHD1	(27) (26)		(29) (30) (31)		
NRG1xSKN7	(27) (26)		(32)		
NRG1xSUT1	(27)		(30) (31) (32)		
NRG1xYDR026C		(35)	(29) (30)		
NRG1xYJL103C		(35)	(30)		
OAF1xPDR1	(27)		(31)		
OAF1xRDS1		(35)	(31)		
OAF1xUME6		(34) (35)	(30) (31)		
OPI1xSUT1	(27)		(32)		
OPI1xSUT2		(34)	(32)		
PBF1xSTB3		(34)	(29) (30) (31)	(30) (29)	
PBF2xSTB3		(34) (35)	(29) (30) (31)	(30) (29)	
PDR1xPDR3		(34)	(30) (31)		
PDR1xRGM1	(27) (26)		(29) (30) (31)		
PDR1xSTB1	(27) (26)		(29)		
PDR1xSTP1	(27) (26)		(30) (31)		
PDR1xSUT1		(34)	(29) (30) (31)		
PDR1xYER184C		(34) (35)	(30) (31)		
PDR1xYJL103C		(35)	(30) (32)		
PDR3xPDR8		(34) (35)	(31)		
PDR3xRDR1		(34)	(32)		
PDR3xRDS1	(27)	(34)			
PDR3xRGT1		(35)	(31)	(30)	
PDR3xSUT1		(35)	(30) (31)		
PDR3xUME6		(34)	(30) (32)		

PDR8xPHO4		(35)	(31)		
PDR8xRDS1		(34)	(30)		
PDR8xSTP1		(35)	(29) (30) (31) (32)		
PDR8xTYE7		(34)	(30) (31)	(30)	
PDR8xUME6		(34)	(32)		
PDR8xYER184C		(35)	(29)		
PDR8xYPR196W		(34)	(31)	(30)	
PHD1xRGM1	(26)		(29) (30) (31)		
PHD1xSKN7	(27) (26)		(31) (32)		
PHD1xSOK2	(27) (26)		(31) (32)		
PHD1xTYE7		(34)	(30) (32)		
PHD1xZMS1		(35)	(29) (30) (31)		
PHO4xRAP1		(34) (35)	(32)		
PHO4xSFP1		(34) (35)	(32)	(30)	
PHO4xSTP1		(35)	(31) (32)		
PHO4xSUT1		(34) (35)	(29) (31) (32)		
PHO4xTYE7		(34) (35)	(29) (31) (32)		
RAP1xRCS1		(34) (35)	(29) (32)		
RAP1xRPH1	(27) (26) (28)	(35)			
RAP1xRSC3		(35)	(31)		
RAP1xYJL103C		(34) (35)	(29)		
RCS1xRPH1	(27)		(31) (32)	(29)	
RCS1xSOK2		(34)	(30) (31) (32)	(30)	
RCS1xSTP2	(26)		(29) (31) (32)		
RDR1xUGA3		(35)	(29) (31) (32)		
RDS1xREB1	(27)		(30)		
RDS1xRGT1		(35)	(30) (31)		
RDS1xRPN4		(35)	(29) (32)		
RDS1xSTB2	(27)		(31) (32)		
RDS1xSWI4		(34)	(29) (32)	(29)	
RDS1xYER184C		(35)	(32)		
RDS2xSKN7		(35)	(30) (31) (32)		
REB1xSKN7	(28)	(35)			
REB1xSTB3		(34)	(29) (30) (31)	(30) (29)	
REB1xSTP1	(28)		(32)		
REB1xSWI4	(28)		(31) (32)		
REB1xSWI6		(34)	(31) (32)	(29)	
REB1xUME6	(28)		(29) (31) (32)		
RGM1xSUT1		(34) (35)	(29) (30) (31) (32)	(29)	
RGM1xYER184C		(34) (35)	(30) (31) (32)	(30)	
RGM1xYJL103C		(35)	(30) (31) (32)	(30)	

RGM1xZMS1	(27) (26)		(30) (31)	(30)	
RGT1xYJL103C		(35)	(31)	(29)	
RGT1xYPR196W		(35)	(31)		
RPH1xSUT1		(35)	(29) (30) (31) (32)		
RPH1xYER184C		(35)	(30) (32)		
RPH1xYJL103C		(35)	(30) (32)		
RPH1xYPL230W		(35)	(29) (30) (31) (32)	(30)	
RPN4xSUT1		(34) (35)	(30) (31) (32)		
RPN4xYDR026C		(34) (35)	(29) (30) (31) (32)		
RPN4xZMS1		(34) (35)	(30) (32)		
RSC3xSFP1		(35)	(29)		
RTG3xSKN7	(27)		(32)		
RTG3xSUM1	(27) (26)		(30) (32)	(29)	
RTG3xSUT1		(34) (35)	(29)		
RTG3xSWI6		(34)	(29) (32)		
RTG3xTYE7	(27)		(31) (32)		
SIG1xXBP1		(34)	(29)		
SIP4xSTP1	(27) (26)		(32)		
SKN7xSOK2	(27) (26)		(32)	(30)	
SKN7xSTB1	(26)		(32)		
SKN7xSTP1	(28)		(29) (32)		
SKN7xSUT1	(27)		(29) (32)	(30)	
SKN7xSWI4	(27) (26) (28)		(31) (32)	(29)	
SKN7xSWI5	(27) (26) (28)		(29) (32)		
SKN7xSWI6	(27) (26)		(32)		
SKN7xUME6	(27) (28)		(30) (32)		
SKN7xYJL103C		(34) (35)	(29)		
SNT2xSTB5		(34)	(30) (31)		
SOK2xSUT1	(27)		(32)		
STB1xSTB2		(34)	(31) (32)		
STB1xXBP1		(35)	(29) (30) (32)		
STB2xSTB3		(34)	(29) (30) (31)	(30) (29)	
STB2xSWI4		(34)	(30) (31) (32)	(30)	
STB2xSWI6		(34) (35)	(29) (30) (31) (32)	(30) (29)	
STB2xYER184C	(27)		(32)		
STB2xYRR1	(27)		(32)		
STB3xSWI6		(34)	(30)		
STB3xYBL054W		(34)	(29) (30) (31)	(30) (29)	
STB4xUGA3		(35)	(30) (31)		
STB4xYER184C	(27)		(29) (32)		
STP1xSUM1	(27) (26)		(32)	(30)	

STP1xSUT1		(35)	(29) (30) (31) (32)	(30) (29)	
STP1xSWI4	(28)		(32)		
STP1xUGA3	(27) (28)		(29) (30) (31) (32)		
STP1xUME6	(28)		(30) (31) (32)		
STP1xYER184C		(35)	(29) (30) (31)		
STP1xYJL103C		(35)	(29) (30) (31)		
STP2xZMS1		(34)	(29) (30) (31) (32)		
SUM1xSWI6		(35)	(32)	(29)	
SUT1xSWI4	(27)		(29) (32)	(29)	
SUT1xSWI6	(27)		(29) (32)		
SUT1xYER130C		(35)	(29) (30) (31)	(30) (29)	
SUT1xYER184C		(34) (35)	(30) (31) (32)		
SUT1xYJL103C		(34) (35)	(30) (31)		
SUT1xYPL230W		(34) (35)	(29) (30) (31)	(29)	
SUT1xYPR196W		(35)	(30) (31) (32)		
SUT1xZMS1		(34) (35)	(29) (30) (31) (32)	(30)	
SWI4xUGA3	(28)		(29) (31) (32)		
SWI4xXBP1	(28)		(29) (30) (31)		
SWI6xYDR026C		(34)	(31)		
UGA3xYER184C		(35)	(31)		
UGA3xYJL103C		(35)	(31)		
UME6xYER184C		(34)	(30) (31)		
UME6xYPR196W		(34) (35)	(30) (31)	(30)	
USV1xZMS1		(35)	(29) (30) (31)		
YAP3xYAP7		(34)	(32)		
YER184CxYPL230W		(34) (35)	(30) (31)	(30)	
YJL103CxYPL230W		(35)	(30) (31)	(30)	
YJL103CxYPR196W		(35)	(30) (31)		
YJL103CxZMS1		(34) (35)	(30) (31)		
YPR196WxYRM1		(34)	(30)	(29)	
ABF1xDIG1		(34)			
ABF1xHAC1			(31)		
ABF1xHMS1		(34)			
ABF1xIME1		(35)			
ABF1xMATALPHA2		(34)			
ABF1xMBP1			(30) (31)		
ABF1xMIG1			(32)		
ABF1xPDR1		(34)			

ABF1xPDR3		(34)			
ABF1xRDS1		(34)			
ABF1xREB1			(32)		
ABF1xRGM1			(32)	(30)	
ABF1xRTG3			(32)		
ABF1xSKN7			(32)		
ABF1xSTB1			(30) (31)		
ABF1xSTP1			(32)		
ABF1xSUM1			(29) (30) (31) (32)		
ABF1xSUT1			(31)		
ABF1xSWI6			(30) (32)		
ABF1xTYE7	(27)				
ABF1xYDR026C			(29)		
ABF1xYER184C			(32)		
ABF1xZAP1	(26)				
ABF2xXBP1			(29)		
ACA1xAFT2			(29)		
ACA1xCAT8			(30) (31) (32)		
ACA1xGAL80		(35)			
ACA1xGIS1			(30)		
ACA1xMET31			(29) (30)		
ACA1xMIG1			(29)		
ACA1xMIG3			(29) (30)		
ACA1xMSN2			(30) (31)		
ACA1xMSN4			(29) (30) (31)		
ACA1xNHP10			(30) (31)		
ACA1xRDS1			(29) (30) (31)		
ACA1xRGM1			(30)		
ACA1xRPH1		(35)			
ACA1xSTP3			(31)	(29)	
ACA1xSUT1			(29) (30) (31) (32)		
ACA1xSWI6			(30) (32)		
ACA1xUME6			(32)		
ACA1xYPL230W			(30)		
ACA1xZMS1			(29)		
ACE2xCBF1			(32)		
ACE2xMET28			(29)		
ACE2xRSC3			(29)		
ACE2xRSC30			(29)		
ACE2xTYE7			(29) (32)		
AFT1xAFT2		(34)			

AFT1xRCS1		(34)		(29)	
AFT2xCAT8			(31)		
AFT2xINO2			(31) (32)		
AFT2xMIG3			(29) (31)		
AFT2xPHD1			(29) (31)		
AFT2xPHO4			(29) (31) (32)	(30)	
AFT2xRSC3			(29)		
AFT2xSTB2			(29) (32)		
AFT2xSTP1			(31) (32)		
AFT2xSUT1			(29) (31) (32)		
AFT2xZMS1			(31) (32)		
ARG80xSTB3		(34)			
ARG80xSUT2			(32)		
ARG80xZMS1			(29) (31)		
ARG81xCAT8			(31)		
ARG81xMIG1			(31)	(29)	
ARG81xRDS1			(30)		
ARG81xRPH1	(27) (26)				
ARG81xSTP2	(27) (26)				
ARG81xSTP3			(31)		
ARG81xSUT1			(29) (31)		
ARG81xYJL103C			(32)		
ARO80xIME1			(29)		
ARO80xPHD1			(32)		
ARO80xUME6			(29)		
ARO80xYRM1		(35)			
ASG1xGIS1		(34) (35)			
ASG1xPHD1		(35)			
ASG1xRGM1		(34) (35)			
ASG1xSTB5		(35)			
ASG1xSTP1		(34) (35)			
ASG1xSTP3		(35)			
ASG1xYPL230W		(35)			
ASH1xCBF1			(32)		
ASH1xMCM1			(32)		
ASH1xOPI1			(32)		
ASH1xTYE7			(32)		
ASH1xYPR196W	(27)				
CAD1xCBF1	(27)				
CAT8xDAL82			(30)		
CAT8xHAC1			(31)		

CAT8xNHP10			(30) (31)		
CAT8xNRG1			(30) (31)		
CAT8xPDR3			(30) (31) (32)		
CAT8xRCS1			(31)		
CAT8xRDS2			(30) (32)		
CAT8xSKN7			(30) (31) (32)		
CAT8xSTB4			(31)		
CAT8xSTB5			(30) (31) (32)		
CAT8xSTP2			(30) (31)		
CAT8xUGA3			(30) (31)		
CAT8xYDR026C			(31)	(29)	
CAT8xYJL103C		(35)			
CAT8xYLL054C			(31)		
CAT8xYPR196W			(30) (31)		
CAT8xYRM1			(30)		
CAT8xZMS1			(30) (31)		
CBF1xCRZ1			(30) (31) (32)		
CBF1xFHL1			(29)		
CBF1xHAP1		(34) (35)			
CBF1xHMS1			(29) (31)	(29)	
CBF1xMBP1			(29) (32)		
CBF1xMET31	(27) (26)				(58)
CBF1xPDR3			(32)		
CBF1xRDS2			(31)		
CBF1xREB1			(32)		
CBF1xRPH1			(29) (30) (31) (32)		
CBF1xSFP1			(29) (32)		
CBF1xSTB2			(32)		
CBF1xSTB4			(31)		
CBF1xSTP1			(29) (31) (32)		
CBF1xYDR026C			(32)		
CBF1xYDR520C			(32)		
CBF1xYJL103C		(34) (35)			
CBF1xYOX1			(32)		
CBF1xYRM1			(30) (31)		
CEP3xMIG3		(35)			
CEP3xRDS1		(34)			
CEP3xSTB4			(29)		
CEP3xYNR063W		(34) (35)			
CHA4xGIS1		(34) (35)			
CHA4xYPL230W		(34) (35)			

CIN5xMET4			(29)		
CIN5xNHP6A	(28)				
CIN5xPDR8			(30) (31)		
CIN5xSTB2			(30)		
CIN5xYPR196W			(31)	(30)	
CRZ1xGIS1			(29) (30) (31)		
CRZ1xINO2			(30) (31) (32)		
CRZ1xMSN2			(29) (30) (31) (32)	(30)	
CRZ1xPDR3			(30) (32)		
CRZ1xRDS1			(30)		
CRZ1xRGM1			(29) (30) (31) (32)		
CRZ1xSTP1			(30) (31) (32)		
CRZ1xSUT1			(29) (30) (31) (32)		
CRZ1xTYE7			(31)		
CRZ1xYPL230W			(29) (30) (31)		
CST6xYLL054C		(35)			
CUP9xRSC3		(35)			
CUP9xRSC30		(35)			
DAL80xGAT1			(30) (32)		
DAL80xXBP1			(31) (32)	(30) (29)	
DAL82xHAP3	(26)				
DAL82xIME1			(29)		
DAL82xLYS14			(31)		
DAL82xMBP1			(29)		
DAL82xMCM1			(29) (31)		
DAL82xPDR1			(29) (30) (31)		
DAL82xPHD1			(31)	(29)	
DAL82xREB1			(29)		
DAL82xRSC3			(30)		
DAL82xSTB1			(29) (32)		
DAL82xSUT1			(30) (31)		
DAL82xSWI4			(29)		
DAL82xTYE7			(32)		
DAL82xYER184C			(31)		
ECM23xGAT4		(34)			
ECM23xGCR1		(34)			
ECM23xMET4		(35)			
EDS1xGAL80	(27)				
EDS1xMBP1		(34)		(29)	
EDS1xRGT1			(29)	(29)	
FHL1xINO4	(28)				

FHL1xTYE7			(29)		
FHL1xYJL103C		(34) (35)			
FKH1xHCM1		(34)		(30)	
FKH1xNDD1	(27) (26)				(56)
FKH2xNDD1	(27) (26)			(29)	(56)
GAL4xMSN4	(27) (26)				
GAL4xRGM1	(27) (26)				
GAL80xGIS1		(34)			
GAL80xIME1		(35)			
GAL80xSKN7		(34)			
GAL80xYER184C		(34)			
GAL80xYJL103C		(34)			
GAL80xYPL230W		(34)			
GAT3xMCM1			(32)		
GAT3xRLM1			(29)		
GAT4xMOT3		(34)			
GCN4xRSC3			(31)		
GCN4xTBF1			(29)		
GCR1xLYS14		(34)			
GCR1xSTB4		(34)			
GCR2xSNF1			(32)		
GCR2xSTB4			(29)		
GCR2xYBL054W		(34)		(30) (29)	
GIS1xMIG2			(30) (31)		
GIS1xMSN4		(35)		(30) (29)	
GIS1xNRG1			(29) (30) (31)		
GIS1xOAF1			(30) (31)	(30)	
GIS1xOPI1			(29)		
GIS1xPDR1			(29) (30) (31)		
GIS1xPHD1			(29) (30) (31)		
GIS1xRDS1			(30) (31)		
GIS1xRDS2			(29) (30) (31)	(30)	
GIS1xRSC3			(29) (30) (31)	(30)	
GIS1xSKN7			(30) (31)		
GIS1xSOK2			(31)		
GIS1xSTP1			(29) (30) (31)	(30)	
GIS1xTBF1			(30)	(30)	
GIS1xUGA3			(29) (30) (31)		
GIS1xUME6			(30) (31)		
GIS1xYDR026C			(30) (31)	(30)	
GIS1xZMS1			(30) (31)	(30)	

GSM1xMIG1			(30) (31)		
GSM1xRDS1		(35)			
GSM1xREB1			(29)		
GTS1xMET32			(32)	(29)	
HAC1xMIG1			(29) (31)		
HAC1xPHD1			(31)		
HAC1xSWI6			(29)		
HAC1xTYE7	(27)				
HAP1xMIG3		(34) (35)			
HAP1xREB1			(29)		
HAP1xRSC3			(31)	(30)	
HAP1xSFP1			(29)		
HAP1xZMS1			(29) (31)		
HAP2xRDS1			(30)		
HAP2xYLL054C		(35)			
HAP3xRSC3			(29) (30) (31)		
HAP3xSWI6		(35)			
HAP3xYAP3			(32)		
HAP3xYBL054W		(35)			
HAP4xYLL054C			(30)		
HAP5xPHD1			(32)		
HAP5xRSC3			(29) (30) (31)		
HAP5xSTP2	(27) (26)				
HAP5xYAP3			(32)		
HAP5xYDR026C		(34) (35)			
HCM1xMCM1		(35)		(30)	
HMRA2xNHP6B			(30) (31)		
HMS1xMIG1			(29) (30) (31)	(30)	
HMS1xRSC3			(30)		
HMS1xYOX1	(27)				
HMS1xZMS1			(30) (31)		
HSF1xYRR1	(27)				
IME1xMCM1			(29) (30) (32)		
IME1xNDD1			(29) (31)		
IME1xPDR1			(30)		
IME1xRPN4			(30) (31) (32)		
IME1xRSC3			(29) (30) (31)		
IME1xRSC30			(29) (30) (31)		
IME1xSTP1			(30) (31)		
IME1xUGA3			(29) (30) (31)		
INO2xOAF1			(32)		

INO2xRCS1			(31) (32)		
INO2xRDS1		(34)			
INO2xREB1	(28)				
INO2xSTB2			(32)		
INO2xSTB4			(32)		
INO2xYDR026C			(32)		
INO4xPHO4		(35)			
INO4xRAP1	(27) (26) (28)				
INO4xREB1	(28)				
INO4xXBP1	(28)				
INO4xYLL054C		(34)			
LEU3xMIG3	(27)				
LEU3xSTB1			(29)		
LEU3xSUT2		(34) (35)			
LEU3xYJL103C		(35)			
LYS14xNHP10			(30) (31)	(29)	
LYS14xNRG1		(35)			
LYS14xPDR8		(35)			
LYS14xSTB4		(34)			
LYS14xSUT1			(30) (31)		
LYS14xSUT2		(35)			
LYS14xYLL054C			(31)		
LYS14xYRM1			(31)		
MATA1xRLM1	(26)				
MBP1xNDT80			(30)		
MBP1xRDS1			(32)		
MBP1xSTB3			(30)		
MBP1xSTP1			(31)		
MBP1xSTP2			(31)		
MBP1xSUT1			(29) (31) (32)		
MBP1xTYE7			(29)		
MCM1xMSN2			(29) (30) (31)		
MCM1xPHD1			(31) (32)		
MCM1xRDS2			(30) (32)		
MCM1xSTB1			(29) (32)		
MCM1xSTP2			(32)		
MCM1xUGA3			(29) (31) (32)		
MCM1xUME6			(30) (32)		
MCM1xXBP1			(29)		
MCM1xYPR196W			(32)		
MET28xPHO4			(31) (32)		

MET28xSTB5		(34)		(30) (29)	
MET28xSUT1			(29) (30) (32)		
MET28xYNR063 W		(35)			
MET31xMIG1			(29) (30) (31)		
MET31xMSN4			(29) (30) (31)		
MET31xPHO4			(31) (32)		
MET31xRSC3			(30)		
MET31xSKN7			(31) (32)		
MET31xSTP3			(31)		
MET31xTYE7			(32)	(30)	
MET4xSKN7			(31)		
MET4xSTB2			(31)		
MIG1xOAF1			(30) (31) (32)		
MIG1xOPI1			(32)		
MIG1xPDR3			(31)		
MIG1xPDR8			(30) (31) (32)		
MIG1xRPH1			(29) (30) (31) (32)		
MIG1xSIP4		(35)			
MIG1xSTB2			(32)		
MIG1xSTB5			(30) (31)		
MIG1xSUT2			(30) (31) (32)		
MIG1xUME6			(30) (31) (32)		
MIG1xYDR026C			(32)		
MIG1xYRM1			(30) (31)	(29)	
MIG2xRGM1			(30) (31)		
MIG2xSTP2			(32)		
MIG2xSUT1			(30) (31) (32)		
MIG2xYPL230W			(30) (31)		
MIG3xOAF1			(30) (31)	(29)	
MIG3xOPI1	(27)				
MIG3xRPH1			(29) (30) (31)		
MIG3xRPN4		(35)			
MIG3xRSC3			(31)		
MIG3xSKN7			(29)		
MIG3xSTB2			(29)		
MIG3xSTP3			(30) (31)		
MIG3xSUT2			(30) (31)		
MIG3xUGA3			(29) (30) (31)		
MIG3xUME6			(30) (31)		
MIG3xYDR520C			(30)		

MIG3xYER130C			(30) (31)		
MIG3xYRM1			(30) (31)		
MOT3xUSV1		(35)			
MSN2xMSN4	(27) (26)			(30) (29)	
MSN2xREB1	(28)				
MSN2xRSC3			(29) (30) (31)	(30) (29)	
MSN2xSOK2			(31) (32)	(30)	
MSN2xYDR026C			(29) (30) (31)		
MSN2xYER184C			(30) (31)		
MSN4xRDS1			(30) (31)		
MSN4xRSC3			(29) (30) (31)	(30) (29)	
MSN4xSTP1			(29) (30) (31)	(30)	
MSN4xYDR026C			(29) (30) (31) (32)		
MSN4xYLL054C			(30)		
MSN4xYPL230W		(35)		(30) (29)	
NDD1xRDS2			(31)		
NDD1xYLL054C		(35)			
NHP10xOAF1			(30) (31)		
NHP10xRDS1			(30)		
NHP10xRDS2		(34)			
NHP10xSKN7			(29)		
NHP10xSTB5			(29) (30) (31)		
NHP10xSTP1			(30) (31)		
NHP10xSTP2			(29) (30) (31)		
NHP10xYRM1		(34)			
NHP10xZMS1			(30)		
NHP6AxNHP6B		(34)			
NHP6AxSPT15		(34) (35)			
NHP6BxPHO2		(34) (35)			
NHP6BxRLM1			(29) (30) (31)		
NRG1xOPI1			(32)		
NRG1xRDR1			(32)		
NRG1xREB1			(32)		
NRG1xRGM1			(29) (30) (31)		
NRG1xRSC3			(30) (31)		
NRG1xSTB2			(29) (32)		
NRG1xTBF1			(30)		
NRG1xYBL054W			(32)		
NRG1xYER184C			(29) (30) (31)		
NRG1xYPL230W			(29) (30) (31)		
NRG1xZMS1			(29) (30) (31) (32)		

OAF1xOPI1			(32)		
OAF1xPDR8			(30) (31) (32)	(30) (29)	
OAF1xRGT1			(31)		
OAF1xSTB5			(30)		
OAF1xSTP1			(29) (30) (31) (32)		
OAF1xSUT1			(29) (30) (31) (32)	(30) (29)	
OAF1xYPL230W			(30) (31)	(30)	
OAF1xYPR196W			(31) (32)		
OAF1xZMS1			(29) (30) (31) (32)	(30)	
OPI1xREB1			(29)		
OPI1xRGM1			(29)		
OPI1xRSC3		(35)			
OPI1xRSC30		(35)			
OPI1xSFP1			(32)		
OPI1xSTB2			(32)		
OPI1xYBL054W			(32)		
OPI1xYPL230W			(29)		
OPI1xYPR196W			(32)		
OPI1xZMS1			(31) (32)		
PBF1xPBF2		(34)			
PDR1xRDR1			(30) (31)		
PDR1xRDS1			(30)		
PDR1xREB1			(31)		
PDR1xRGT1			(31)		
PDR1xRSC3			(29) (30) (31)	(30) (29)	
PDR1xRSC30			(29) (30) (31)		
PDR1xSWI4			(29)		
PDR1xUGA3			(29) (31)		
PDR1xUME6		(34)			
PDR1xYDR026C			(32)		
PDR1xYPL230W			(29) (30) (31)		
PDR1xYRM1			(30) (31)		
PDR3xPHO4			(32)		
PDR3xRSC3			(31)		
PDR3xRTG3			(32)		
PDR3xSKO1			(32)		
PDR3xSUT2			(30) (31)		
PDR3xYER184C			(31) (32)		
PDR3xYJL103C			(31)		
PDR3xYRM1		(34)			
PDR8xRDR1			(29)		

PDR8xREB1			(29)		
PDR8xRGT1			(30) (31) (32)		
PDR8xSTB5			(30) (31) (32)		
PDR8xSUT1			(30) (31)	(30)	
PDR8xSUT2			(32)		
PDR8xYJL103C		(35)			
PDR8xYRM1			(30) (31)		
PHD1xPHO4			(30) (31) (32)		
PHD1xRDS1			(29) (30) (31)		
PHD1xREB1			(31) (32)		
PHD1xRPH1			(31)		
PHD1xRSC3			(29)		
PHD1xSTB2			(31)		
PHD1xSTP1			(31) (32)		
PHD1xSTP2			(29) (30) (31)		
PHD1xUGA3			(31)		
PHD1xXBP1			(31)		
PHD1xYER184C			(31)		
PHD1xYJL103C			(31)	(30)	
PHD1xYPL230W			(29) (30) (31)		
PHO2xSPT15		(34) (35)			
PHO4xRSC3			(30)		
PHO4xSKN7			(31) (32)		
PHO4xSTB4			(31)	(30)	
PHO4xSTP2			(30) (31) (32)		
PHO4xYDR026C		(34)			
RAP1xSTP2		(35)			
RAP1xTYE7			(29) (32)		
RCS1xRSC3			(29)		
RCS1xSUT1			(29) (30) (31) (32)		
RCS1xZMS1			(30) (31) (32)	(30)	
RDR1xSTB1			(32)		
RDR1xSTP1			(32)		
RDR1xSUT1			(30) (31) (32)		
RDS1xRDS2			(30) (31)	(29)	
RDS1xRGM1			(30) (31) (32)		
RDS1xRPH1			(30)		
RDS1xRSC3		(35)			
RDS1xRSC30		(35)			
RDS1xSKN7			(29)		
RDS1xSTB1			(29)		

RDS1xSTB5			(29) (30) (31)		
RDS1xSTP2			(32)		
RDS1xSUT1			(29) (30) (31) (32)		
RDS1xUGA3			(31)		
RDS1xUME6		(34)			
RDS1xYDR026C			(30) (31)		
RDS1xYDR520C			(32)		
RDS1xYJL103C		(35)			
RDS1xYPL230W			(30) (31)		
RDS1xYRM1			(29) (30)		
RDS1xZMS1			(30) (31)		
RDS2xRGM1			(29) (30) (31)	(30)	
RDS2xSTB4			(30)		
RDS2xSTB5			(32)		
RDS2xSTP1			(31) (32)	(29)	
RDS2xSUT1			(31)		
RDS2xTYE7			(31) (32)		
RDS2xYJL103C			(30)		
RDS2xYPL230W			(29) (30) (31)	(30)	
RDS2xYPR196W			(30) (31)		
RDS2xZMS1			(29) (30) (31)		
REB1xRGM1			(32)		
REB1xSTB1			(31)	(30)	
REB1xSTB2		(34)		(30)	
REB1xSTB5			(32)		
REB1xSTP2			(31)		
REB1xSUM1			(32)	(29)	
REB1xTBF1			(29)		
REB1xYER184C			(32)		
REB1xYPR196W			(29)		
REB1xYRM1			(29)		
RGM1xRSC3			(29) (30) (31)	(30)	
RGM1xSKN7			(30) (31)		
RGM1xSOK2			(31) (32)		
RGM1xSTP1			(29) (30) (31) (32)	(30)	
RGM1xTBF1			(30)	(30)	
RGM1xUGA3			(29) (30) (31)		
RGM1xUME6			(30) (31) (32)		
RGM1xYDR026C			(30) (31) (32)	(30)	
RGM1xYER130C			(32)		
RGT1xRSC3			(30) (31)		

RGT1xSKN7			(32)		
RGT1xSTB3		(34)			
RGT1xSTP1			(32)		
RGT1xUGA3			(31)		
RGT1xUME6			(32)		
RGT1xYRM1			(31)	(30)	
RGT1xZMS1			(31)		
RPH1xRSC3			(31)		
RPH1xYER130C			(30) (31) (32)		
RPN4xUME6			(30) (31) (32)		
RSC30xSUT1		(35)			
RSC30xUME6			(29) (31)	(30) (29)	
RSC30xYRM1			(29) (30) (31)		
RSC30xZMS1			(31)		
RSC3xRTG3			(29)		
RSC3xSKN7			(31)		
RSC3xSTB5			(31)		
RSC3xSTP1			(29)	(29)	
RSC3xSTP2			(29)		
RSC3xSUT1			(30) (31)		
RSC3xSWI5			(29)		
RSC3xSWI6			(29) (31)		
RSC3xXBP1			(31)		
RSC3xYLL054C			(31)		
RSC3xYPL230W			(29) (30) (31)	(30)	
RSC3xYPR196W			(31)	(30) (29)	
RSC3xYRM1			(29) (30) (31)		
RSC3xZMS1			(29) (30) (31)		
RTG3xSTB1			(29) (30) (32)	(30) (29)	
RTG3xSWI4			(29) (30) (32)	(29)	
SFP1xTYE7			(32)		
SIP4xSKN7		(35)			
SKN7xSTB4			(29)		
SKN7xSTB5			(32)		
SKN7xSUT2			(30) (32)		
SKN7xYDR520C			(31)		
SKN7xYER184C			(29) (31)		
SKN7xYPL230W			(30) (31)		
SKO1xSUM1			(30) (32)		
SNT2xSTP3		(34)			
SOK2xYPL230W			(31) (32)		

SOK2xZMS1			(32)		
SPT15xUPC2			(29) (30) (31)		
SPT23xSTB3			(29)		
SRD1xYER130C			(30) (31)		
STB1xSTB3			(30) (31)	(29)	
STB1xSTP1			(32)		
STB1xSUT1			(29) (32)		
STB1xUGA3			(29) (32)		
STB1xYDR026C			(32)		
STB1xYER184C			(29)		
STB1xYRM1			(29)		
STB2xSTP1			(32)		
STB2xSTP2			(31) (32)		
STB2xSUM1	(27)				
STB2xSUT1			(32)		
STB2xTYE7	(27)				
STB2xUME6			(29)		
STB2xYDR026C			(32)		
STB2xZMS1			(32)		
STB3xSWI4			(30) (31)	(29)	
STB3xXBP1			(30) (32)		
STB3xYDR026C			(29)		
STB3xYRR1		(35)			
STB4xSTB5	(27)				
STB4xSUT1			(32)		
STB4xSUT2			(32)		
STB4xYJL103C			(29) (32)		
STB5xSTP2			(30) (31) (32)		
STB5xSUT1			(31)		
STB5xSUT2			(30)		
STB5xYDR520C			(30) (31) (32)		
STP1xSWI6			(32)		
STP1xTYE7			(31) (32)		
STP1xYLL054C			(29)		
STP1xYPL230W			(29) (30) (31)	(30)	
STP2xSUM1			(32)		
STP2xSUT1			(29) (30) (31) (32)		
STP2xYER184C			(32)		
STP2xYJL103C			(30)		
STP3xSUT1			(29) (31)		
SUT1xSUT2			(30) (31) (32)	(29)	

SUT1xTYE7			(29) (31) (32)		
SUT1xUGA3			(30) (31)		
SUT1xUME6			(30) (31) (32)	(30)	
SUT1xYDR520C			(32)		
SUT1xYRM1			(30) (31)		
SUT2xXBP1			(32)		
SUT2xYER184C			(30) (32)		
SUT2xZMS1			(30) (31) (32)		
SWI4xYDR026C			(32)		
SWI6xUGA3			(29) (31)		
TBF1xTYE7			(29)		
TBF1xYDR026C			(29)		
TBF1xYPL230W			(30)	(30)	
TEC1xUPC2			(31)	(30) (29)	
TEC1xYER130C			(30) (32)		
TYE7xUME6		(35)		(29)	
TYE7xYER184C		(35)			
TYE7xYJL103C		(35)			
UGA3xYPL230W			(29) (30) (31)		
UME6xYPL230W			(30) (31)		
XBP1xYBL054W			(31) (32)		
YDR026CxYER130C			(29)		
YDR026CxYER184C			(29) (32)		
YDR026CxYJL103C			(29) (32)		
YDR026CxYPL230W			(30) (31) (32)	(30)	
YER130CxZMS1			(30) (31)	(30) (29)	
YER184CxYJL103C			(32)		
YER184CxZMS1			(31)		
YPL230WxZMS1			(30) (31) (32)	(30)	
YRM1xZMS1			(30)		
ABF1xAFT2					
ABF1xCRZ1					
ABF1xGIS1					
ABF1xHAP3					
ABF1xMET28					
ABF1xMIG3					
ABF1xPHO4					

ABF1xRSC3					
ABF1xRSC30					
ABF1xSKO1					
ABF1xSTB2					
ABF1xSTP2					
ABF1xYJL103C					
ABF1xYLL054C					
ABF1xZMS1					
ACA1xOPI1					
ACA1xRSC3					
ACA1xYER184C					
ACA1xYJL103C					
ACA1xYLL054C					
ACE2xMET31					
AFT2xMET31					
ARG81xOPI1					
ARG81xRSC3					
ARG81xRSC30					
ARG81xSTB4					
ARG81xUME6					
ARO80xMIG1					
ARO80xRDR1					
ARO80xRDS1					
ARO80xRSC3					
ARO80xSTB4					
ARO80xSUT1					
ARO80xYER184C					
ARR1xNHP6B					
ASH1xDAL82					
ASH1xNHP10					
ASH1xRSC3					
AZF1xECM23					
AZF1xEDS1					
CAD1xUSV1					
CAD1xYKL222C					
CAT8xLYS14					
CAT8xOPI1					
CAT8xREB1					
CAT8xSTB1					
CAT8xSTB2					
CAT8xYBL054W					

CAT8xYDR520C					
CBF1xDAL82					
CBF1xRSC3					
CBF1xTBF1					
CBF1xYLL054C					
CEP3xSTB3					
CIN5xHMRA2					
CIN5xREB1					
CIN5xYDR026C					
CRZ1xPDR1					
CRZ1xSWI5					
CST6xNDD1					
CUP9xECM23					
CUP9xGAT3					
DAL82xEDS1					
DAL82xOPI1					
DAL82xSTB2					
DAL82xSTB3					
ECM23xMET32					
EDS1xRDR1					
EDS1xSTP1					
EDS1xYRM1					
FHL1xOPI1					
FHL1xSTP2					
GAL4xGIS1					
GAL4xRSC3					
GAL4xSUT1					
GAL4xYJL103C					
GAL4xYPL230W					
GAL80xPDR1					
GAL80xRSC3					
GAT3xRSC30					
GAT4xMET32					
GCR2xRDR1					
GCR2xRFX1					
GIS1xMSN2					
GIS1xREB1					
GIS1xYER130C					
GIS1xYLL054C					
GSM1xYJL103C					
GTS1xMET4					

HAC1xMBP1					
HAC1xSUT1					
HAP1xNHP10					
HAP1xPDR3					
HAP1xUGA3					
HAP2xRSC3					
HAP4xRSC3					
HAP5xUGA3					
HAP5xYBL054W					
HCM1xNDD1					
HMRA2xMATALPHA2					
HMRA2xNHP6A					
HMRA2xSPT15					
IME1xSKN7					
IME1xSTB2					
IME1xYLL054C					
INO2xPHD1					
INO2xPHO4					
INO2xYLL054C					
INO4xSTB2					
INO4xYDR026C					
LEU3xRSC3					
LEU3xTYE7					
LYS14xMCM1					
LYS14xSKN7					
MATALPHA2xNHP6A					
MATALPHA2xSPT15					
MATALPHA2xTYE7					
MBP1xRDS2					
MBP1xRSC3					
MBP1xSUM1					
MCM1xNHP10					
MCM1xPDR1					
MCM1xRSC3					
MCM1xSIP4					
MCM1xSUT1					
MCM1xYLL054C					
MET28xRSC3					

MET28xTYE7					
MET31xRDS1					
MET4xRSC3					
MIG1xNHP10					
MIG1xRDS2					
MIG1xREB1					
MIG1xRSC3					
MIG1xRSC30					
MIG1xYBL054W					
MIG1xYLL054C					
MIG2xSUT2					
MIG2xYAP7					
MIG2xYJL103C					
MIG3xRDS2					
MIG3xREB1					
MIG3xYDR026C					
MIG3xYLL054C					
MSN2xRGM1					
MSN2xYPL230W					
MSN4xREB1					
NDD1xRSC3					
NDD1xRSC30					
NDD1xSTP1					
NDD1xSTP2					
NDT80xSKN7					
NHP10xPDR8					
NHP10xRSC3					
NHP10xRSC30					
NHP10xYBL054W					
NHP10xYPR196W					
NHP6AxRLM1					
NRG1xRDS1					
OAF1xTYE7					
OAF1xYJL103C					
OPI1xRDS2					
OPI1xSTP3					
OPI1xYDR026C					
OPI1xYER184C					
OPI1xYJL103C					
PBF2xRTG1					
PBF2xXBP1					

PBF2xYBL054W					
PDR1xRDS2					
PDR1xRTG3					
PDR1xSKN7					
PDR1xSTB2					
PDR1xSTB4					
PDR1xYLL054C					
PDR3xSKN7					
PDR3xSTB5					
PDR3xTYE7					
PDR8xRSC3					
PDR8xRSC30					
PDR8xSTB1					
PHD1xRSC30					
PHD1xYDR026C					
PHO4xRDS1					
PHO4xREB1					
PHO4xSTB2					
PHO4xTBF1					
PHO4xYLL054C					
PHO4xYRM1					
RAP1xYLL054C					
RDR1xRGT1					
RDR1xRSC3					
RDR1xRSC30					
RDR1xSKN7					
RDR1xYRM1					
RDS1xSWI6					
RDS1xXBP1					
RDS1xYLL054C					
RDS2xSNT2					
RDS2xSTB3					
RDS2xYDR026C					
REB1xRSC3					
REB1xRSC30					
REB1xSNT2					
REB1xSUT1					
REB1xTYE7					
REB1xYDR026C					
REB1xYJL103C					
REB1xYPL230W					

REB1xYRR1					
REB1xZMS1					
RFX1xRSC3					
RGM1xYLL054C					
RGT1xSTB1					
RGT1xSUT2					
RLM1xSPT15					
RPH1xSTP3					
RSC30xRSC3					(59)
RSC30xSKN7					
RSC30xSTB2					
RSC30xSTP1					
RSC30xSTP2					
RSC30xUGA3					
RSC30xYER051W					
RSC30xYER184C					
RSC30xYJL103C					
RSC3xSTB1					
RSC3xSTB2					
RSC3xSUT2					
RSC3xSWI4					
RSC3xTHI2					
RSC3xUGA3					
RSC3xUME6					
RSC3xYBL054W					
RSC3xYDR026C					
RSC3xYER051W					
RSC3xYER184C					
RSC3xYJL103C					
SKN7xYBL054W					
SKN7xYDR026C					
SKN7xYLL054C					
SKN7xYRM1					
SNT2xSTB2					
SNT2xYER184C					
STB1xYLL054C					
STB2xTBF1					
STB2xYJL103C					
STB5xYER184C					
STB5xYJL103C					
STP1xSTP3					

STP1xYDR026C					
STP2xYLL054C					
SUT1xYDR026C					
SUT1xYLL054C					
SUT2xYJL103C					
SWI4xYLL054C					
SWI5xYLL054C					
TBF1xYRR1					
TYE7xUGA3					
TYE7xYDR026C					
TYE7xYDR520C					
TYE7xYLL054C					
TYE7xYRM1					
TYE7xYRR1					
UGA3xYLL054C					
UME6xYDR026C					
UME6xYLL054C					
YBL054WxYLL054C					
YDR026CxYLL054C					
YDR026CxZMS1					
YDR520CxZMS1					
YER130CxYPL230W					
YER184CxYLL054C					
YER184CxYRM1					
YJL103CxYLL054C					
YJL103CxYRM1					
YKL222CxYRM1					
YLL054CxYPL230W					
YLL054CxZMS1					

References

1. Gertz, J., Siggia, E.D. and Cohen, B.A. (2009) Analysis of combinatorial cis-regulation in synthetic and genomic promoters. *Nature*, **457**, 215-218.

2. Pilpel, Y., Sudarsanam, P. and Church, G.M. (2001) Identifying regulatory networks by combinatorial analysis of promoter elements. *Nat Genet*, **29**, 153-159.

3. Parker, D.S., White, M.A., Ramos, A.I., Cohen, B.A. and Barolo, S. (2011) The cis-regulatory logic of Hedgehog gradient responses: key roles for gli binding affinity, competition, and cooperativity. *Science signaling*, **4**, ra38.

4. Yuh, C.H., Bolouri, H. and Davidson, E.H. (1998) Genomic cis-regulatory logic: experimental and computational analysis of a sea urchin gene. *Science*, **279**, 1896-1902.

5. Gertz, J. and Cohen, B.A. (2009) Environment-specific combinatorial cis-regulation in synthetic promoters. *Molecular systems biology*, **5**, 244.

6. Pettersson, M. and Schaffner, W. (1990) Synergistic activation of transcription by multiple binding sites for NF-kappa B even in absence of co-operative factor binding to DNA. *Journal of molecular biology*, **214**, 373-380.

7. Aguilar, D. and Oliva, B. (2008) Topological comparison of methods for predicting transcriptional cooperativity in yeast. *BMC genomics*, **9**, 137.

8. Drazinic, C.M., Smerage, J.B., Lopez, M.C. and Baker, H.V. (1996) Activation mechanism of the multifunctional transcription factor repressor-activator protein 1 (Rap1p). *Mol Cell Biol*, **16**, 3187-3196.

9. Magbanua, J.P., Ogawa, N., Harashima, S. and Oshima, Y. (1997) The transcriptional activators of the PHO regulon, Pho4p and Pho2p, interact directly with each other and with components of the basal transcription machinery in Saccharomyces cerevisiae. *Journal of biochemistry*, **121**, 1182-1189.

10. Yu, X., Lin, J., Masuda, T., Esumi, N., Zack, D.J. and Qian, J. (2006) Genome-wide prediction and characterization of interactions between transcription factors in Saccharomyces cerevisiae. *Nucleic Acids Res*, **34**, 917-927.

11. Tirosh, I., Berman, J. and Barkai, N. (2007) The pattern and evolution of yeast promoter bendability. *Trends in genetics : TIG*, **23**, 318-321.

12. Shea, M.A. and Ackers, G.K. (1985) The OR control system of bacteriophage lambda. A physical-chemical model for gene regulation. *Journal of molecular biology*, **181**, 211-230.

13. Banerjee, N. and Zhang, M.Q. (2003) Identifying cooperativity among transcription factors controlling the cell cycle in yeast. *Nucleic Acids Res*, **31**, 7024-7031.

14. Beer, M.A. and Tavazoie, S. (2004) Predicting gene expression from sequence. *Cell*, **117**, 185-198.

15. GuhaThakurta, D. and Stormo, G.D. (2001) Identifying target sites for cooperatively binding factors. *Bioinformatics*, **17**, 608-621.

16. Wang, Y., Zhang, X.S. and Xia, Y. (2009) Predicting eukaryotic transcriptional cooperativity by Bayesian network integration of genome-wide data. *Nucleic Acids Res*, **37**, 5943-5958.

17. Kellis, M., Patterson, N., Endrizzi, M., Birren, B. and Lander, E.S. (2003) Sequencing and comparison of yeast species to identify genes and regulatory elements. *Nature*, **423**, 241-254.

18. Doniger, S.W. and Fay, J.C. (2007) Frequent gain and loss of functional transcription factor binding sites. *PLoS computational biology*, **3**, e99.

19. Moses, A.M., Pollard, D.A., Nix, D.A., Iyer, V.N., Li, X.Y., Biggin, M.D. and Eisen, M.B. (2006) Large-scale turnover of functional transcription factor binding sites in Drosophila. *PLoS computational biology*, **2**, e130.

20. Odom, D.T., Dowell, R.D., Jacobsen, E.S., Gordon, W., Danford, T.W., MacIsaac, K.D., Rolfe, P.A., Conboy, C.M., Gifford, D.K. and Fraenkel, E. (2007) Tissue-specific transcriptional regulation has diverged significantly between human and mouse. *Nat Genet*, **39**, 730-732.

21. Tuch, B.B., Galgoczy, D.J., Hernday, A.D., Li, H. and Johnson, A.D. (2008) The evolution of combinatorial gene regulation in fungi. *PLoS Biol*, **6**, e38.

22. Kim, J., He, X. and Sinha, S. (2009) Evolution of regulatory sequences in 12 Drosophila species. *PLoS genetics*, **5**, e1000330.

23. He, Q., Bardet, A.F., Patton, B., Purvis, J., Johnston, J., Paulson, A., Gogol, M., Stark, A. and Zeitlinger, J. (2011) High conservation of transcription factor binding and evidence for combinatorial regulation across six Drosophila species. *Nat Genet*, **43**, 414-420.

24. Spivak, A.T. and Stormo, G.D. (2012) ScerTF: a comprehensive database of benchmarked position weight matrices for Saccharomyces species. *Nucleic Acids Res*, **40**, D162-168.

25. Sarafova, S. and Siu, G. (2000) Precise arrangement of factor-binding sites is required for murine CD4 promoter function. *Nucleic Acids Res*, **28**, 2664-2671.

26. Harbison, C.T., Gordon, D.B., Lee, T.I., Rinaldi, N.J., Macisaac, K.D., Danford, T.W., Hannett, N.M., Tagne, J.B., Reynolds, D.B., Yoo, J. *et al.* (2004) Transcriptional regulatory code of a eukaryotic genome. *Nature*, **431**, 99-104.

27. Lee, T.I., Rinaldi, N.J., Robert, F., Odom, D.T., Bar-Joseph, Z., Gerber, G.K., Hannett, N.M., Harbison, C.T., Thompson, C.M., Simon, I. *et al.* (2002) Transcriptional regulatory networks in Saccharomyces cerevisiae. *Science*, **298**, 799-804.

28. Venters, B.J., Wachi, S., Mavrich, T.N., Andersen, B.E., Jena, P., Sinnamon, A.J., Jain, P., Rolleri, N.S., Jiang, C., Hemeryck-Walsh, C. *et al.* (2011) A comprehensive genomic binding map of gene and chromatin regulatory proteins in Saccharomyces. *Mol Cell*, **41**, 480-492.

29. Pramila, T., Wu, W., Miles, S., Noble, W.S. and Breeden, L.L. (2006) The Forkhead transcription factor Hcm1 regulates chromosome segregation genes and fills the S-phase gap in the transcriptional circuitry of the cell cycle. *Genes Dev*, **20**, 2266-2278.

30. Gasch, A.P., Spellman, P.T., Kao, C.M., Carmel-Harel, O., Eisen, M.B., Storz, G., Botstein, D. and Brown, P.O. (2000) Genomic expression programs in the response of yeast cells to environmental changes. *Mol Biol Cell*, **11**, 4241-4257.

31. Hughes, T.R., Marton, M.J., Jones, A.R., Roberts, C.J., Stoughton, R., Armour, C.D., Bennett, H.A., Coffey, E., Dai, H., He, Y.D. *et al.* (2000) Functional discovery via a compendium of expression profiles. *Cell*, **102**, 109-126.

32. Reimand, J., Vaquerizas, J.M., Todd, A.E., Vilo, J. and Luscombe, N.M. (2010) Comprehensive reanalysis of transcription factor knockout expression data in Saccharomyces cerevisiae reveals many new targets. *Nucleic Acids Res*, **38**, 4768-4777.

33. Hu, Z., Killion, P.J. and Iyer, V.R. (2007) Genetic reconstruction of a functional transcriptional regulatory network. *Nat Genet*, **39**, 683-687.

34. Ashburner, M., Ball, C.A., Blake, J.A., Botstein, D., Butler, H., Cherry, J.M., Davis, A.P., Dolinski, K., Dwight, S.S., Eppig, J.T. *et al.* (2000) Gene ontology: tool for the unification of biology. The Gene Ontology Consortium. *Nat Genet*, **25**, 25-29.

35. Kanehisa, M. and Goto, S. (2000) KEGG: kyoto encyclopedia of genes and genomes. *Nucleic Acids Res*, **28**, 27-30.

36. Liko, D., Slattery, M.G. and Heideman, W. (2007) Stb3 binds to ribosomal RNA processing element motifs that control transcriptional responses to growth in Saccharomyces cerevisiae. *The Journal of biological chemistry*, **282**, 26623-26628.

37. Zhu, C., Byers, K.J., McCord, R.P., Shi, Z., Berger, M.F., Newburger, D.E., Saulrieta, K., Smith, Z., Shah, M.V., Radhakrishnan, M. *et al.* (2009) High-resolution DNA-binding specificity analysis of yeast transcription factors. *Genome Res*, **19**, 556-566.

38. Tavazoie, S., Hughes, J.D., Campbell, M.J., Cho, R.J. and Church, G.M. (1999) Systematic determination of genetic network architecture. *Nat Genet*, **22**, 281-285.

39. Koch, C., Moll, T., Neuberg, M., Ahorn, H. and Nasmyth, K. (1993) A role for the transcription factors Mbp1 and Swi4 in progression from G1 to S phase. *Science*, **261**, 1551-1557.

40. de Bruin, R.A., Kalashnikova, T.I. and Wittenberg, C. (2008) Stb1 collaborates with other regulators to modulate the G1-specific transcriptional circuit. *Mol Cell Biol*, **28**, 6919-6928.

41. Ho, Y., Costanzo, M., Moore, L., Kobayashi, R. and Andrews, B.J. (1999) Regulation of transcription at the Saccharomyces cerevisiae start transition by Stb1, a Swi6-binding protein. *Mol Cell Biol*, **19**, 5267-5278.

42. Wade, J.T., Hall, D.B. and Struhl, K. (2004) The transcription factor Ifh1 is a key regulator of yeast ribosomal protein genes. *Nature*, **432**, 1054-1058.

43. Jorgensen, P., Rupes, I., Sharom, J.R., Schneper, L., Broach, J.R. and Tyers, M. (2004) A dynamic transcriptional network communicates growth potential to ribosome synthesis and critical cell size. *Genes Dev*, **18**, 2491-2505.

44. Olson, K.A., Nelson, C., Tai, G., Hung, W., Yong, C., Astell, C. and Sadowski, I. (2000) Two regulators of Ste12p inhibit pheromone-responsive transcription by separate mechanisms. *Mol Cell Biol*, **20**, 4199-4209.

45. Chou, S., Lane, S. and Liu, H. (2006) Regulation of mating and filamentation genes by two distinct Ste12 complexes in Saccharomyces cerevisiae. *Mol Cell Biol*, **26**, 4794-4805.

46. Rubin-Bejerano, I., Mandel, S., Robzyk, K. and Kassir, Y. (1996) Induction of meiosis in Saccharomyces cerevisiae depends on conversion of the transcriptional represssor Ume6 to a positive regulator by its regulated association with the transcriptional activator Ime1. *Mol Cell Biol*, **16**, 2518-2526.

47. Coic, E., Sun, K., Wu, C. and Haber, J.E. (2006) Cell cycle-dependent regulation of Saccharomyces cerevisiae donor preference during mating-type switching by SBF (Swi4/Swi6) and Fkh1. *Mol Cell Biol*, **26**, 5470-5480.

48. Ho, Y., Gruhler, A., Heilbut, A., Bader, G.D., Moore, L., Adams, S.L., Millar, A., Taylor, P., Bennett, K., Boutilier, K. *et al.* (2002) Systematic identification of protein complexes in Saccharomyces cerevisiae by mass spectrometry. *Nature*, **415**, 180-183.

49. Yoon, S., Govind, C.K., Qiu, H., Kim, S.J., Dong, J. and Hinnebusch, A.G. (2004) Recruitment of the ArgR/Mcm1p repressor is stimulated by the activator Gcn4p: a self-checking activation mechanism. *Proc Natl Acad Sci U S A*, **101**, 11713-11718.

50. Joo, Y.J., Kim, J.A., Baek, J.H., Seong, K.M., Han, K.D., Song, J.M., Choi, J.Y. and Kim, J. (2009) Cooperative regulation of ADE3 transcription by Gcn4p and Bas1p in Saccharomyces cerevisiae. *Eukaryotic cell*, **8**, 1268-1277.

51. Ozcan, S. and Johnston, M. (1996) Two different repressors collaborate to restrict expression of the yeast glucose transporter genes HXT2 and HXT4 to low levels of glucose. *Mol Cell Biol*, **16**, 5536-5545.

52. Shahi, P., Gulshan, K., Naar, A.M. and Moye-Rowley, W.S. (2010) Differential roles of transcriptional mediator subunits in regulation of multidrug resistance gene expression in Saccharomyces cerevisiae. *Mol Biol Cell*, **21**, 2469-2482.

53. Zhu, G., Spellman, P.T., Volpe, T., Brown, P.O., Botstein, D., Davis, T.N. and Futcher, B. (2000) Two yeast forkhead genes regulate the cell cycle and pseudohyphal growth. *Nature*, **406**, 90-94.

54. Mead, J., Zhong, H., Acton, T.B. and Vershon, A.K. (1996) The yeast alpha2 and Mcm1 proteins interact through a region similar to a motif found in homeodomain proteins of higher eukaryotes. *Mol Cell Biol*, **16**, 2135-2143.

55. Pramila, T., Miles, S., GuhaThakurta, D., Jemiolo, D. and Breeden, L.L. (2002) Conserved homeodomain proteins interact with MADS box protein Mcm1 to restrict ECB-dependent transcription to the M/G1 phase of the cell cycle. *Genes Dev*, **16**, 3034-3045.

56. Kumar, R., Reynolds, D.M., Shevchenko, A., Goldstone, S.D. and Dalton, S. (2000) Forkhead transcription factors, Fkh1p and Fkh2p, collaborate with Mcm1p to control transcription required for M-phase. *Current biology : CB*, **10**, 896-906.

57. Graves, J.A. and Henry, S.A. (2000) Regulation of the yeast INO1 gene. The products of the INO2, INO4 and OPI1 regulatory genes are not required for repression in response to inositol. *Genetics*, **154**, 1485-1495.

58. Lee, T.A., Jorgensen, P., Bognar, A.L., Peyraud, C., Thomas, D. and Tyers, M. (2010) Dissection of combinatorial control by the Met4 transcriptional complex. *Mol Biol Cell*, **21**, 456-469.

59. Angus-Hill, M.L., Schlichter, A., Roberts, D., Erdjument-Bromage, H., Tempst, P. and Cairns, B.R. (2001) A Rsc3/Rsc30 zinc cluster dimer reveals novel roles for the chromatin remodeler RSC in gene expression and cell cycle control. *Mol Cell*, **7**, 741-751.

60. Menon, B.B., Sarma, N.J., Pasula, S., Deminoff, S.J., Willis, K.A., Barbara, K.E., Andrews, B. and Santangelo, G.M. (2005) Reverse recruitment: the Nup84 nuclear pore subcomplex mediates Rap1/Gcr1/Gcr2 transcriptional activation. *Proc Natl Acad Sci U S A*, **102**, 5749-5754.

61. Breitkreutz, B.J., Stark, C., Reguly, T., Boucher, L., Breitkreutz, A., Livstone, M., Oughtred, R., Lackner, D.H., Bahler, J., Wood, V. *et al.* (2008) The BioGRID Interaction Database: 2008 update. *Nucleic Acids Res*, **36**, D637-640.

62. Robinson, K.A., Koepke, J.I., Kharodawala, M. and Lopes, J.M. (2000) A network of yeast basic helix-loop-helix interactions. *Nucleic Acids Res*, **28**, 4460-4466.

63. Badis, G., Chan, E.T., van Bakel, H., Pena-Castillo, L., Tillo, D., Tsui, K., Carlson, C.D., Gossett, A.J., Hasinoff, M.J., Warren, C.L. *et al.* (2008) A library of yeast transcription factor motifs reveals a widespread function for Rsc3 in targeting nucleosome exclusion at promoters. *Mol Cell*, **32**, 878-887.

64. Cairns, B.R., Lorch, Y., Li, Y., Zhang, M., Lacomis, L., Erdjument-Bromage, H., Tempst, P., Du, J., Laurent, B. and Kornberg, R.D. (1996) RSC, an essential, abundant chromatin-remodeling complex. *Cell*, **87**, 1249-1260.

65. Krogan, N.J., Cagney, G., Yu, H., Zhong, G., Guo, X., Ignatchenko, A., Li, J., Pu, S., Datta, N., Tikuisis, A.P. *et al.* (2006) Global landscape of protein complexes in the yeast Saccharomyces cerevisiae. *Nature*, **440**, 637-643.

66. Zheng, J., Benschop, J.J., Shales, M., Kemmeren, P., Greenblatt, J., Cagney, G., Holstege, F., Li, H. and Krogan, N.J. (2010) Epistatic relationships reveal the functional organization of yeast transcription factors. *Molecular systems biology*, **6**, 420.

67. Cherry, J.M., Hong, E.L., Amundsen, C., Balakrishnan, R., Binkley, G., Chan, E.T., Christie, K.R., Costanzo, M.C., Dwight, S.S., Engel, S.R. *et al.* (2012) Saccharomyces Genome Database: the genomics resource of budding yeast. *Nucleic Acids Res*, **40**, D700-705.

68. Su, N.Y., Ouni, I., Papagiannis, C.V. and Kaiser, P. (2008) A dominant suppressor mutation of the met30 cell cycle defect suggests regulation of the Saccharomyces cerevisiae Met4-Cbf1 transcription complex by Met32. *The Journal of biological chemistry*, **283**, 11615-11624.

69. Sudarsanam, P., Pilpel, Y. and Church, G.M. (2002) Genome-wide co-occurrence of promoter elements reveals a cis-regulatory cassette of rRNA transcription motifs in Saccharomyces cerevisiae. *Genome Res*, **12**, 1723-1731.

70. Hahn, S. and Young, E.T. (2011) Transcriptional regulation in Saccharomyces cerevisiae: transcription factor regulation and function, mechanisms of initiation, and roles of activators and coactivators. *Genetics*, **189**, 705-736.

71. Hollenhorst, P.C., Pietz, G. and Fox, C.A. (2001) Mechanisms controlling differential promoter-occupancy by the yeast forkhead proteins Fkh1p and Fkh2p: implications for regulating the cell cycle and differentiation. *Genes Dev*, **15**, 2445-2456.
