# Supplementary material for: Combinatorial Cis-regulation in Saccharomyces Species
Source: G3 (Bethesda). 2016 Jan 12;6(3):653–67. doi: 10.1534/g3.115.024331 (PMC4777128; doi:10.1534/g3.115.024331)
Supplement: Supporting Information [file supp_g3.115.024331_TableS2.zip › TableS2.pdf]

Table S2. List of TF pairs identified from 7 Genome search, and number of co-occurrences in promoters for each species. (Data also available in TableS2.xls)

TFPair	Cer.	Klu.	Mik.	Par.	Cas.	Bay.	Kud.
ABF1xACA1	197	399	274	153	225	303	286
ABF1xAFT2	224	486	235	239	194	351	211
ABF1xBAS1	166	508	301	172	169	318	238
ABF1xCBF1	264	429	230	274	202	452	287
ABF1xCRZ1	206	389	255	141	167	239	244
ABF1xCST6	176	171	133	139	106	185	189
ABF1xDIG1	74	50	38	47	24	49	30
ABF1xGCN4	183	393	235	142	166	204	216
ABF1xGIS1	522	559	344	583	444	717	513
ABF1xHAC1	167	425	206	167	181	275	291
ABF1xHAP3	9	2	3	10	6	9	8
ABF1xHAP5	9	5	5	11	4	18	16
ABF1xHMS1	130	132	94	141	114	259	130
ABF1xHSF1	59	51	31	44	20	45	33
ABF1xIME1	92	33	46	94	32	115	71
ABF1xINO2	155	187	124	182	116	236	147
ABF1xINO4	120	130	73	146	71	140	121
ABF1xMATALPHA2	905	1080	550	932	659	862	707
ABF1xMBP1	137	330	199	146	143	260	253
ABF1xMET28	155	328	189	132	128	206	195
ABF1xMIG1	228	383	261	242	176	436	241
ABF1xMIG3	168	206	166	163	182	270	252
ABF1xPDR1	201	367	226	148	173	215	240
ABF1xPDR3	55	22	28	46	52	67	27
ABF1xPHD1	170	395	237	134	188	290	237
ABF1xPHO4	198	272	133	216	122	341	169
ABF1xRDS1	122	92	84	121	83	224	138
ABF1xREB1	127	201	143	135	136	177	180
ABF1xRGM1	522	559	344	583	444	717	513
ABF1xRPN4	153	174	99	162	76	173	126
ABF1xRSC30	23	10	3	17	7	68	19

ABF1xRSC3	177	311	273	153	216	354	302
ABF1xRTG3	155	373	222	154	159	246	199
ABF1xSKN7	157	388	251	171	179	317	276
ABF1xSKO1	165	453	232	189	141	327	248
ABF1xSTB1	140	339	229	139	185	226	248
ABF1xSTB2	117	295	191	133	150	181	220
ABF1xSTB3	219	164	142	190	159	247	187
ABF1xSTP1	181	326	187	159	186	315	246
ABF1xSTP2	54	60	36	68	37	137	95
ABF1xSUM1	70	68	41	81	75	82	69
ABF1xSUT1	187	336	243	182	193	314	290
ABF1xSWI4	215	342	232	135	168	261	232
ABF1xSWI6	138	320	212	150	145	208	217
ABF1xTBF1	192	370	209	152	175	233	185
ABF1xTYE7	243	405	203	224	188	389	279
ABF1xUME6	140	36	49	148	85	173	133
ABF1xYDR026C	144	180	101	160	99	191	120
ABF1xYER184C	238	394	294	164	224	354	296
ABF1xYJL103C	428	546	257	436	277	549	329
ABF1xYLL054C	16	28	2	26	21	83	27
ABF1xYPR196W	142	308	220	124	153	202	216
ABF1xZAP1	6	6	1	8	1	2	2
ABF1xZMS1	177	445	253	212	245	309	318
ABF2xXBP1	315	587	575	344	298	278	517
ACA1xAFT2	269	583	480	284	345	392	327
ACA1xCAT8	194	539	442	174	329	290	343
ACA1xGAL80	18	11	17	8	16	38	4
ACA1xGCN4	223	477	335	151	283	241	279
ACA1xGIS1	601	726	683	663	744	826	709
ACA1xMET31	187	463	334	175	316	313	325
ACA1xMIG1	265	579	421	280	302	483	358
ACA1xMIG3	193	332	279	158	297	321	370
ACA1xMSN2	214	532	544	244	398	300	396
ACA1xMSN4	333	475	461	357	398	462	396
ACA1xNHP10	62	87	62	47	72	105	97

ACA1xOPI1	52	111	52	58	95	49	58
ACA1xPHD1	208	574	450	162	352	363	347
ACA1xRDS1	126	165	172	167	121	274	196
ACA1xRGM1	601	726	683	663	744	826	709
ACA1xRPH1	183	226	151	183	185	338	207
ACA1xRPN4	103	187	131	115	118	153	123
ACA1xRSC3	178	679	481	138	323	352	385
ACA1xRTG3	166	484	419	187	280	280	305
ACA1xSKN7	182	581	443	194	404	319	298
ACA1xSTP1	206	458	372	158	295	320	327
ACA1xSTP3	288	422	452	309	409	323	289
ACA1xSUT1	211	641	458	156	361	334	335
ACA1xSWI6	162	448	373	171	268	229	280
ACA1xUME6	85	46	86	78	86	133	121
ACA1xYER184C	246	556	478	166	466	434	439
ACA1xYJL103C	423	642	441	504	565	703	547
ACA1xYLL054C	28	29	20	30	36	82	44
ACA1xYPL230W	601	726	683	663	744	826	709
ACA1xZMS1	227	633	483	233	397	381	467
ACE2xCBF1	159	419	259	190	294	266	298
ACE2xMET28	151	340	334	212	328	298	324
ACE2xMET31	172	342	345	204	337	309	394
ACE2xRSC30	39	21	25	19	26	47	31
ACE2xRSC3	232	485	522	157	384	289	388
ACE2xSKN7	174	443	537	226	400	322	432
ACE2xSWI5	370	581	572	664	555	608	512
ACE2xTYE7	165	399	221	168	262	260	262
AFT1xAFT2	88	43	44	85	48	87	45
AFT1xRCS1	61	40	40	58	36	75	48
AFT2xCAT8	312	584	472	290	381	379	366
AFT2xCBF1	304	574	235	350	259	532	291
AFT2xFHL1	353	426	279	246	333	361	289
AFT2xINO2	251	290	164	300	168	303	205
AFT2xMET31	280	505	305	254	326	363	302
AFT2xMIG1	396	626	435	466	392	645	349

AFT2xMIG3	327	349	293	307	360	417	359
AFT2xPHD1	326	590	415	257	394	466	366
AFT2xPHO4	283	385	149	289	170	404	201
AFT2xRAP1	243	161	107	126	145	192	93
AFT2xRCS1	383	694	478	371	352	535	342
AFT2xRPH1	319	299	162	301	209	407	208
AFT2xRPN4	161	199	111	188	94	204	111
AFT2xRSC3	329	642	452	333	357	507	366
AFT2xSFP1	296	516	367	242	332	320	318
AFT2xSTB2	253	475	362	263	323	317	311
AFT2xSTP1	292	555	321	319	335	395	333
AFT2xSUT1	351	614	500	332	394	491	350
AFT2xTYE7	278	520	193	302	236	426	232
AFT2xYJL103C	668	673	447	945	552	856	486
AFT2xZMS1	388	830	500	412	489	537	424
ARG80xBAS1	234	477	431	226	228	357	320
ARG80xGCN4	265	363	349	174	214	263	305
ARG80xMIG1	261	389	365	216	254	387	317
ARG80xMIG3	211	209	232	124	254	235	298
ARG80xSTB3	160	183	200	151	210	239	222
ARG80xSUT1	208	345	382	138	235	262	332
ARG80xSUT2	56	80	51	63	90	59	56
ARG80xZMS1	241	419	382	197	297	307	401
ARG81xBAS1	151	159	151	179	137	237	175
ARG81xCAT8	155	165	113	138	156	237	161
ARG81xGCN4	151	150	120	137	148	151	165
ARG81xMIG1	178	185	123	233	136	305	137
ARG81xMIG3	125	107	76	163	136	174	156
ARG81xOPI1	40	32	22	58	28	36	32
ARG81xPHO4	122	101	53	125	94	164	92
ARG81xRDS1	90	43	40	102	90	185	68
ARG81xRPH1	98	88	40	176	84	203	101
ARG81xRSC30	36	8	11	20	20	52	15
ARG81xRSC3	159	157	139	145	166	246	213
ARG81xRTG3	119	138	125	158	140	173	191

ARG81xSKN7	142	169	129	173	187	241	153	
ARG81xSTB4	105	48	46	103	63	111	68	
ARG81xSTP1	152	149	102	158	135	253	193	
ARG81xSTP2	39	20	24	63	29	69	49	
ARG81xSTP3	209	148	115	242	158	239	139	
ARG81xSUT1	153	147	123	145	153	260	151	
ARG81xUME6	69	12	24	81	37	95	47	
ARG81xYJL103C	321	215	118	457	231	400	215	
ARO80xCST6	10	12	7	19	9	13	21	
ARO80xIME1	13	4	0	10	5	18	5	
ARO80xMIG1	22	22	16	29	19	35	31	
ARO80xPDR1	21	28	9	36	9	36	15	
ARO80xPDR3	15	3	0	14	7	4	4	
ARO80xPHD1	19	16	9	16	8	42	16	
ARO80xRDR1	21	14	5	11	5	19	17	
ARO80xRDS1	29	10	3	34	4	37	11	
ARO80xRSC3	23	13	14	21	12	36	12	
ARO80xSTB4	8	7	7	24	5	20	10	
ARO80xSTP1	30	25	13	22	12	39	22	
ARO80xSUT1	28	20	10	24	19	36	30	
ARO80xUME6	13	0	5	20	9	10	9	
ARO80xYER184C		24	26	8	15	9	43	31
ARO80xYRM1	11	27	6	21	5	25	11	
ARR1xNHP6B	30	45	15	25	35	35	40	
ASG1xGIS1	1	7	0	5	11	12	3	
ASG1xPHD1	2	7	2	0	6	2	3	
ASG1xRGM1	1	7	0	5	11	12	3	
ASG1xSTB5	4	4	4	4	2	2	3	
ASG1xSTP1	2	4	2	0	8	8	3	
ASG1xSTP3	4	5	2	3	2	5	2	
ASG1xYPL230W	1	7	0	5	11	12	3	
ASH1xCBF1	70	47	39	106	59	128	76	
ASH1xDAL82	75	102	73	90	82	113	90	
ASH1xMBP1	93	83	106	98	119	132	101	
ASH1xMCM1	60	15	20	58	72	50	33	

ASH1xNDD1	48	17	18	58	72	54	29	
ASH1xNHP10	25	14	20	34	33	49	25	
ASH1xOPI1	19	10	18	24	26	31	18	
ASH1xRSC3	97	121	77	81	77	133	100	
ASH1xSTP1	67	68	46	77	84	109	70	
ASH1xSWI6	83	84	99	94	94	113	105	
ASH1xTYE7	75	41	38	94	53	81	63	
ASH1xYPR196W		60	65	55	89	94	86	87
AZF1xECM23	99	44	46	135	20	74	66	
AZF1xEDS1	44	37	17	59	10	40	23	
BAS1xGCN4	309	544	471	288	245	400	311	
BAS1xRTG3	206	497	482	255	254	419	308	
BAS1xSTP3	296	490	492	380	295	423	345	
CAD1xCBF1	140	65	63	102	63	115	102	
CAD1xUSV1	8	0	4	4	1	4	5	
CAD1xYAP7	138	26	38	154	78	68	72	
CAD1xYKL222C	3	0	2	11	2	5	2	
CAT8xDAL82	182	421	427	175	265	277	327	
CAT8xGAL4	6	1	9	6	13	13	4	
CAT8xGIS1	667	653	609	634	658	830	760	
CAT8xHAC1	161	469	425	174	308	357	440	
CAT8xLYS14	203	366	322	140	244	263	312	
CAT8xMIG1	327	540	497	286	340	464	392	
CAT8xMIG3	245	313	314	200	299	323	420	
CAT8xMSN2	250	524	480	251	360	287	438	
CAT8xMSN4	386	438	442	355	360	469	438	
CAT8xNHP10	52	83	70	52	64	97	114	
CAT8xNRG1	254	316	357	151	310	286	375	
CAT8xOPI1	56	95	67	51	52	65	56	
CAT8xPDR1	246	487	451	208	327	358	312	
CAT8xPDR3	54	36	52	56	45	71	63	
CAT8xPHD1	213	525	419	169	368	362	385	
CAT8xRCS1	250	511	437	181	324	326	331	
CAT8xRDS1	136	135	133	142	207	321	185	
CAT8xRDS2	257	541	414	168	324	252	368	

CAT8xREB1	149	189	261	127	272	220	248
CAT8xRGM1	667	653	609	634	658	830	760
CAT8xRPH1	206	232	172	161	158	344	256
CAT8xRSC3	267	573	575	180	334	465	445
CAT8xRTG3	144	389	398	166	250	229	310
CAT8xSKN7	246	497	523	192	405	429	389
CAT8xSTB1	143	438	387	167	248	247	333
CAT8xSTB2	142	349	343	136	272	223	314
CAT8xSTB4	152	198	165	141	186	173	158
CAT8xSTB5	180	413	406	147	241	266	285
CAT8xSTP1	239	523	387	216	271	468	360
CAT8xSTP2	61	96	88	66	47	155	111
CAT8xSUT1	256	576	559	204	380	419	381
CAT8xSWI5	353	581	470	455	431	593	469
CAT8xUGA3	93	101	73	93	93	175	86
CAT8xUME6	135	55	92	142	88	158	118
CAT8xYBL054W	235	484	437	160	352	359	397
CAT8xYDR026C	155	177	182	139	198	226	166
CAT8xYDR520C	28	29	20	14	16	33	32
CAT8xYER184C	373	483	533	167	434	443	462
CAT8xYJL103C	589	593	456	523	505	661	546
CAT8xYLL054C	41	63	22	38	62	158	44
CAT8xYPL230W	667	653	609	634	658	830	760
CAT8xYPR196W	199	385	395	157	245	226	276
CAT8xYRM1	188	384	353	169	275	349	354
CAT8xZMS1	277	640	495	251	397	430	494
CBF1xCRZ1	243	529	340	142	334	335	284
CBF1xDAL81	28	2	7	20	5	17	8
CBF1xDAL82	185	407	302	148	335	335	291
CBF1xFHL1	156	353	145	154	245	294	247
CBF1xGAT1	376	541	308	423	315	387	342
CBF1xGCN4	238	406	266	180	237	300	281
CBF1xHAC1	186	602	307	162	254	409	306
CBF1xHAP1	82	91	41	110	46	112	64
CBF1xHAP5	6	21	0	4	6	8	10

CBF1xHMS1	150	172	104	180	107	307	169	
CBF1xINO2	226	373	154	278	232	349	209	
CBF1xINO4	162	221	106	198	142	211	138	
CBF1xMATALPHA2		848	1337	676	1038	851	1098	931
CBF1xMBP1	178	507	228	208	247	382	265	
CBF1xMET28	212	416	255	139	227	314	230	
CBF1xMET31	188	421	211	166	245	336	268	
CBF1xMET32	278	327	172	250	306	332	228	
CBF1xMET4	54	46	32	71	55	71	20	
CBF1xOAF1	86	198	54	118	135	134	112	
CBF1xPDR3	112	35	40	104	53	113	53	
CBF1xPDR8	159	368	185	149	248	332	252	
CBF1xPHD1	228	499	293	146	339	380	338	
CBF1xPHO4	311	539	205	338	223	518	263	
CBF1xRAP1	104	102	46	80	95	134	77	
CBF1xRDS2	184	554	234	178	232	272	306	
CBF1xREB1	242	230	257	270	275	354	269	
CBF1xRPH1	222	189	91	232	141	309	236	
CBF1xRSC3	156	503	365	192	386	511	394	
CBF1xRTG3	180	397	308	202	271	400	264	
CBF1xSFP1	188	473	198	210	226	302	249	
CBF1xSTB2	246	369	306	266	245	312	308	
CBF1xSTB4	134	229	101	176	132	185	138	
CBF1xSTP1	234	506	209	180	318	386	304	
CBF1xSUT1	242	443	303	214	386	458	338	
CBF1xTBF1	185	329	234	154	224	313	224	
CBF1xTYE7	439	801	368	414	290	699	303	
CBF1xYDR026C	172	193	135	194	203	322	194	
CBF1xYDR520C	24	40	8	28	10	20	24	
CBF1xYER184C	253	435	293	183	368	499	460	
CBF1xYJL103C	443	640	247	543	422	769	535	
CBF1xYLL054C	20	31	6	56	68	122	27	
CBF1xYOX1	54	34	45	49	41	37	40	
CBF1xYRM1	194	469	233	250	257	409	291	
CEP3xMIG3	179	287	277	170	288	223	375	

CEP3xRDS1	101	129	123	118	104	203	163	
CEP3xSTB3	143	173	201	132	182	225	195	
CEP3xSTB4	134	174	180	153	155	140	158	
CEP3xYNR063W		1	4	7	4	4	5	9
CHA4xGIS1	0	4	0	0	10	10	2	
CHA4xRGM1	0	4	0	0	10	10	2	
CHA4xYPL230W	0	4	0	0	10	10	2	
CIN5xHMRA2	229	174	226	198	377	301	234	
CIN5xMET4	23	11	9	26	33	13	18	
CIN5xMIG3	202	79	130	158	300	155	184	
CIN5xNHP6A	152	125	203	155	242	142	148	
CIN5xNRG1	207	107	137	150	260	169	180	
CIN5xPDR3	65	5	19	34	71	36	17	
CIN5xPDR8	215	111	147	152	271	179	172	
CIN5xREB1	133	82	97	131	310	112	109	
CIN5xRGT1	230	138	196	166	249	153	194	
CIN5xSTB2	146	121	149	144	284	121	149	
CIN5xXBP1	207	162	213	242	463	197	187	
CIN5xYDR026C	86	65	59	84	187	80	54	
CIN5xYPR196W	199	135	194	130	233	152	170	
CRZ1xGIS1	703	695	702	666	688	903	795	
CRZ1xINO2	192	273	223	159	202	219	206	
CRZ1xMSN2	266	559	551	241	364	307	464	
CRZ1xPDR1	223	439	495	165	286	276	369	
CRZ1xPDR3	77	23	55	54	66	63	68	
CRZ1xRDS1	152	126	122	143	132	263	221	
CRZ1xRGM1	703	695	702	666	688	903	795	
CRZ1xSTP1	251	422	412	198	258	375	334	
CRZ1xSUT1	275	462	558	191	306	375	397	
CRZ1xSWI5	440	537	555	475	441	603	537	
CRZ1xTYE7	241	471	269	140	265	261	252	
CRZ1xYJL103C	499	611	501	547	415	643	576	
CRZ1xYPL230W	703	695	702	666	688	903	795	
CST6xNDD1	41	33	38	56	121	81	67	
CST6xSUT1	178	156	218	140	203	249	271	

CST6xSWI6	211	173	210	138	172	226	276
CST6xYER184C	216	154	215	200	249	328	319
CST6xYJL103C	376	230	204	531	335	529	413
CST6xYLL054C	14	13	9	21	26	55	24
CUP9xECM23	191	83	78	163	162	193	84
CUP9xGAT3	29	33	34	35	31	64	31
CUP9xRAP1	24	15	12	7	17	16	4
CUP9xRSC30	10	0	6	12	14	20	3
CUP9xRSC3	56	47	45	44	87	59	46
CUP9xSFP1	36	43	40	26	30	34	25
CUP9xSTP1	40	43	23	32	35	52	32
DAL80xGAT1	511	499	638	686	701	632	457
DAL80xGLN3	311	430	541	413	457	555	443
DAL80xGZF3	514	287	466	691	702	635	459
DAL80xHAP1	72	89	64	84	83	81	61
DAL80xXBP1	245	426	386	312	435	387	314
DAL82xEDS1	415	504	401	456	294	433	471
DAL82xHAP3	7	3	13	5	15	15	16
DAL82xIME1	60	43	55	59	48	95	63
DAL82xLYS14	218	377	389	148	234	286	282
DAL82xMBP1	186	518	397	192	256	253	349
DAL82xMCM1	119	76	82	99	122	103	74
DAL82xOPI1	55	77	46	51	60	69	51
DAL82xPDR1	187	409	378	165	293	247	289
DAL82xPHD1	202	505	442	161	255	292	347
DAL82xRDS1	111	122	93	130	147	259	178
DAL82xREB1	153	192	232	142	209	212	248
DAL82xRSC3	160	543	370	163	333	278	373
DAL82xSTB1	195	445	355	133	249	221	355
DAL82xSTB2	147	338	333	138	224	187	298
DAL82xSTB3	172	141	175	132	209	179	203
DAL82xSTP1	202	472	357	185	239	282	333
DAL82xSUT1	195	499	420	174	311	322	332
DAL82xSWI4	235	443	361	128	230	258	310
DAL82xTYE7	161	359	248	133	253	259	238

DAL82xUME6	89	40	94	101	62	122	88	
DAL82xYER184C		271	436	452	157	328	397	424
DIG1xSTE12	59	67	82	72	50	77	51	
ECM23xGAT4	1065	1112	1124	1069	1576	1206	1178	
ECM23xGCR1	587	327	269	669	513	561	402	
ECM23xMET32	1007	784	564	1044	1237	1036	903	
ECM23xMET4	182	76	53	170	159	155	88	
EDS1xGAL80	26	17	5	25	12	46	19	
EDS1xHSF1	110	41	51	111	56	82	84	
EDS1xMBP1	437	468	397	479	297	480	505	
EDS1xPBF1	194	96	79	197	73	167	108	
EDS1xPBF2	432	463	263	451	260	500	373	
EDS1xRDR1	489	246	219	596	229	460	446	
EDS1xRGT1	604	586	453	611	370	552	568	
EDS1xSTB3	484	206	217	497	273	452	372	
EDS1xSTP1	492	462	321	517	258	475	497	
EDS1xYRM1	486	521	380	544	337	696	612	
FHL1xINO4	106	108	89	129	129	117	125	
FHL1xMET31	184	285	218	137	255	234	282	
FHL1xMIG1	241	373	280	247	304	433	299	
FHL1xMIG3	192	202	186	134	288	253	315	
FHL1xOPI1	59	79	35	63	62	49	53	
FHL1xPHO4	197	223	99	138	132	209	186	
FHL1xRAP1	239	140	84	119	160	124	104	
FHL1xRCS1	208	379	279	156	274	300	279	
FHL1xRPH1	197	152	109	172	165	280	209	
FHL1xRSC3	258	553	241	169	312	298	358	
FHL1xSFP1	297	374	277	200	288	269	275	
FHL1xSTP2	60	66	41	74	38	84	57	
FHL1xTYE7	165	326	113	128	206	239	225	
FHL1xYJL103C	473	456	302	524	438	620	432	
FKH1xFKH2	327	487	433	200	449	372	322	
FKH1xHCM1	754	558	645	763	899	733	632	
FKH1xMBP1	201	413	379	217	328	286	348	
FKH1xMCM1	100	49	60	81	147	113	91	

FKH1xNDD1	78	53	72	62	147	109	69	
FKH1xSTB1	197	398	379	192	340	245	343	
FKH1xSWI4	293	403	390	181	319	310	324	
FKH1xSWI6	204	369	372	189	290	274	302	
FKH2xHCM1	1055	676	727	650	1115	867	628	
FKH2xMBP1	289	463	427	155	402	332	319	
FKH2xMCM1	140	69	73	72	176	127	85	
FKH2xNDD1	126	57	83	63	178	126	67	
FKH2xSTB1	296	467	444	157	428	295	315	
FKH2xSWI4	418	469	452	174	398	367	297	
FKH2xSWI6	295	428	456	168	376	332	280	
FZF1xSPT23	470	377	366	360	348	298	427	
GAL4xGAL80	10	0	8	11	4	15	0	
GAL4xGIS1	16	10	12	17	17	48	3	
GAL4xMSN4	15	7	13	13	9	30	1	
GAL4xPDR1	7	3	6	2	12	15	4	
GAL4xRGM1	16	10	12	17	17	48	3	
GAL4xRSC3	4	1	14	6	8	17	5	
GAL4xSTP1	9	2	18	6	10	15	5	
GAL4xSUT1	4	2	15	1	11	16	6	
GAL4xYJL103C	13	5	0	7	15	32	4	
GAL4xYPL230W	16	10	12	17	17	48	3	
GAL80xGIS1	33	29	10	34	34	119	26	
GAL80xIME1	7	2	6	7	6	7	0	
GAL80xMIG3	46	13	10	17	30	58	15	
GAL80xPDR1	17	14	11	12	25	49	9	
GAL80xRSC3	5	19	22	13	15	57	8	
GAL80xSKN7	50	8	18	9	31	49	12	
GAL80xSTP1	16	19	28	20	18	51	18	
GAL80xSUT1	38	19	26	13	24	64	18	
GAL80xYER184C		34	12	2	4	24	41	14
GAL80xYJL103C	44	17	1	29	31	81	11	
GAL80xYPL230W		33	29	10	34	34	119	26
GAT1xGLN3	562	574	659	656	496	582	461	
GAT1xGZF3	920	378	570	1064	763	657	471	

GAT1xSIP4	22	3	22	29	9	10	11
GAT3xMCM1	62	60	75	91	89	144	80
GAT3xRLM1	198	124	196	174	194	357	263
GAT3xRSC30	44	24	10	19	15	69	23
GAT4xGCR1	139	110	89	89	113	128	120
GAT4xLYS14	276	343	347	138	296	256	327
GAT4xMET32	164	279	200	146	247	197	237
GAT4xMOT3	191	634	727	158	619	224	830
GCN4xLEU3	37	37	41	37	55	44	33
GCN4xRSC3	176	367	425	186	236	242	323
GCN4xRTG3	216	367	395	200	245	285	322
GCN4xSTB3	210	187	203	157	255	249	202
GCN4xTBF1	217	411	329	181	242	262	244
GCN4xTYE7	248	403	224	180	194	248	208
GCR1xGCR2	273	228	130	196	271	155	227
GCR1xLYS14	217	93	81	82	93	137	98
GCR1xRAP1	72	22	18	48	69	72	32
GCR1xSTB4	107	36	39	100	56	76	53
GCR1xSTP2	35	10	18	47	13	53	27
GCR2xRDR1	273	362	316	344	514	290	567
GCR2xRFX1	2	0	11	5	1	4	7
GCR2xSNF1	3	2	1	0	14	1	2
GCR2xSTB4	263	289	244	276	407	205	329
GCR2xTHI2	8	2	5	14	6	8	22
GCR2xYBL054W	480	876	632	230	941	373	762
GIS1xMIG1	942	820	743	1148	820	1390	836
GIS1xMIG2	49	10	15	69	23	54	44
GIS1xMIG3	831	484	486	859	766	1030	909
GIS1xMSN2	937	917	929	1219	906	1146	993
GIS1xMSN4	1367	745	788	1854	906	1773	993
GIS1xNHP10	238	153	86	248	153	311	225
GIS1xNRG1	722	469	509	667	723	790	717
GIS1xOAF1	337	200	164	439	308	379	256
GIS1xOPI1	124	115	74	193	144	149	127
GIS1xPDR1	677	703	605	647	691	903	658

GIS1xPHD1	646	745	623	636	695	1003	740
GIS1xRDS1	451	184	209	580	326	753	337
GIS1xRDS2	831	742	557	699	728	720	752
GIS1xREB1	528	355	384	651	634	719	597
GIS1xRPH1	626	389	292	770	390	969	536
GIS1xRSC3	717	879	764	740	764	946	747
GIS1xSKN7	648	756	675	763	817	1081	762
GIS1xSOK2	1188	729	752	671	670	1479	958
GIS1xSTP1	660	632	530	758	569	951	694
GIS1xSUT1	830	824	790	840	818	1182	820
GIS1xTBF1	538	541	557	610	629	645	623
GIS1xUGA3	266	124	118	374	187	458	175
GIS1xUME6	280	61	115	325	178	409	274
GIS1xYDR026C	469	290	216	561	440	617	376
GIS1xYER130C	214	74	74	242	129	264	154
GIS1xYER184C	926	688	729	792	857	1233	907
GIS1xYJL103C	1519	857	680	2136	1090	1963	1070
GIS1xYLL054C	80	55	39	113	71	232	72
GIS1xZMS1	916	1050	903	1124	930	1425	1158
GLN3xGZF3	567	328	491	657	496	582	461
GSM1xMIG1	64	62	50	65	56	77	39
GSM1xMIG3	57	32	35	41	51	67	42
GSM1xRDS1	23	19	7	35	22	51	20
GSM1xREB1	35	22	22	60	45	34	32
GSM1xSUT1	49	44	55	36	48	72	45
GSM1xYJL103C	125	64	45	161	94	133	86
GTS1xMET32	424	342	219	426	425	394	281
GTS1xMET4	67	26	27	63	57	47	40
HAC1xMBP1	195	466	445	212	302	358	390
HAC1xMIG1	244	532	382	239	314	504	380
HAC1xPHD1	191	502	481	184	277	369	382
HAC1xPHO4	170	366	177	163	137	346	240
HAC1xSUT1	202	494	423	159	273	396	402
HAC1xSWI6	193	424	418	199	270	323	337
HAC1xTYE7	193	535	249	152	206	314	262

HAC1xYLL054C	16	32	15	17	36	99	43
HAP1xMIG1	97	73	72	135	50	168	75
HAP1xMIG3	61	45	49	66	53	117	85
HAP1xNHP10	23	4	12	35	10	40	27
HAP1xPDR1	67	82	54	90	41	95	71
HAP1xPDR3	21	2	9	22	11	34	16
HAP1xPHO4	70	74	38	90	20	107	45
HAP1xRDS1	41	15	23	86	16	108	53
HAP1xREB1	63	42	45	73	41	81	81
HAP1xRSC3	70	105	58	82	62	142	92
HAP1xSFP1	48	78	74	81	34	73	56
HAP1xSKN7	56	85	91	96	57	141	91
HAP1xSOK2	148	83	84	79	31	169	107
HAP1xSUT1	61	98	79	66	44	150	92
HAP1xTOS8	4	2	1	6	3	3	2
HAP1xUGA3	33	18	17	52	6	68	29
HAP1xYER184C	82	100	82	75	70	135	130
HAP1xYJL103C	140	137	84	210	80	203	151
HAP1xZMS1	74	86	94	102	61	156	112
HAP2xRDS1	110	98	148	146	85	211	141
HAP2xRSC3	190	312	412	143	322	205	335
HAP2xSUT1	213	297	413	168	284	254	319
HAP2xYLL054C	20	23	20	29	17	42	32
HAP3xHAP4	12	5	3	12	3	6	8
HAP3xMBP1	2	9	6	4	9	4	9
HAP3xMIG3	9	1	8	7	12	19	10
HAP3xPDR8	9	1	5	6	9	10	8
HAP3xRDS1	4	6	4	5	2	19	3
HAP3xRSC3	15	18	12	12	31	15	13
HAP3xSKN7	8	7	10	8	22	24	10
HAP3xSUT1	14	3	16	8	16	26	9
HAP3xSWI6	4	7	9	1	12	6	8
HAP3xUGA3	2	0	4	5	6	5	5
HAP3xYAP3	7	0	1	5	12	8	4
HAP3xYBL054W	9	8	7	4	10	17	10

HAP3xZMS1	10	3	9	9	13	29	12
HAP4xHAP5	14	0	3	16	5	19	9
HAP4xRDS1	73	36	40	97	49	110	56
HAP4xRSC3	115	127	179	139	186	136	125
HAP4xSKN7	125	129	149	128	149	162	120
HAP4xSUT1	149	127	167	116	151	137	123
HAP4xYLL054C	19	12	8	20	8	27	7
HAP5xNHP10	5	0	3	6	2	5	4
HAP5xPHD1	11	13	11	13	12	18	10
HAP5xRDS1	15	4	9	18	0	24	10
HAP5xRSC3	17	9	14	17	18	14	15
HAP5xSKN7	22	10	17	24	9	34	13
HAP5xSTP2	3	1	5	5	2	5	5
HAP5xSUT1	19	11	24	19	9	25	12
HAP5xUGA3	4	0	5	4	5	8	6
HAP5xYAP3	8	2	2	13	10	15	5
HAP5xYBL054W	6	19	7	7	14	17	7
HAP5xYDR026C	12	5	1	12	8	14	6
HCM1xMCM1	301	80	111	263	318	235	159
HCM1xNDD1	270	67	130	237	314	224	122
HMRA2xMATALPHA2	1274	1385	1148	1330	1641	1628	1310
HMRA2xMCM1	106	45	100	115	157	106	86
HMRA2xNHP6A	192	418	402	214	393	331	381
HMRA2xNHP6B	203	427	370	183	379	312	469
HMRA2xSPT15	203	457	427	179	394	321	339
HMS1xMIG1	212	231	165	266	244	411	225
HMS1xMIG3	182	119	115	199	225	319	249
HMS1xNHP10	52	37	33	54	54	83	67
HMS1xRSC3	152	203	187	151	173	253	204
HMS1xSOK2	318	179	179	191	159	425	242
HMS1xSUT1	182	241	174	188	219	307	195
HMS1xTYE7	144	161	78	148	97	252	148
HMS1xYOX1	45	15	22	47	21	37	30
HMS1xZMS1	194	296	214	246	252	419	286
HSF1xXBP1	287	42	33	143	57	58	35

HSF1xYRR1	5	0	0	6	3	1	7	
IME1xMCM1	37	4	10	23	28	29	21	
IME1xNDD1	34	7	7	21	14	29	16	
IME1xPDR1	82	54	74	76	116	126	96	
IME1xPDR3	37	2	9	24	23	33	17	
IME1xRDS1	47	19	46	71	99	77	43	
IME1xRPN4	34	13	25	50	29	73	34	
IME1xRSC30	10	10	6	9	6	30	6	
IME1xRSC3	82	70	84	64	47	161	109	
IME1xSKN7	59	61	62	72	68	115	66	
IME1xSTB2	58	34	62	66	60	77	84	
IME1xSTP1	81	46	53	77	44	160	63	
IME1xSUT1	77	51	71	80	56	143	103	
IME1xUGA3	30	7	11	33	20	49	16	
IME1xUME6	37	2	9	57	6	65	43	
IME1xYLL054C	5	4	4	17	12	43	3	
IME1xYPR196W	72	39	51	63	66	71	69	
INO2xINO4	126	105	59	153	106	148	111	
INO2xMATALPHA2		833	701	456	1052	535	776	692
INO2xOAF1	72	101	38	102	77	95	70	
INO2xPDR3	44	14	22	52	25	55	24	
INO2xPHD1	181	255	168	115	202	247	221	
INO2xPHO4	166	221	89	186	139	234	162	
INO2xRAP1	104	46	45	66	69	73	51	
INO2xRCS1	191	265	185	160	142	229	175	
INO2xRDS1	141	61	50	153	100	173	127	
INO2xREB1	165	129	113	162	156	190	163	
INO2xSTB2	171	205	168	173	154	170	170	
INO2xSTB4	107	115	68	170	75	130	113	
INO2xTYE7	183	341	110	241	177	278	161	
INO2xYAP7	170	69	85	192	63	90	91	
INO2xYDR026C	136	118	81	158	134	160	109	
INO2xYLL054C	16	12	3	25	40	66	31	
INO4xPHO4	118	131	62	154	83	138	122	
INO4xRAP1	61	22	33	64	42	55	42	

INO4xREB1	108	79	72	122	98	121	116	
INO4xSTB2	111	119	106	136	93	121	134	
INO4xTYE7	131	206	84	168	108	178	101	
INO4xXBP1	137	166	138	197	115	152	167	
INO4xYAP7	127	48	70	147	45	72	82	
INO4xYDR026C	88	69	52	109	89	106	77	
INO4xYLL054C	7	10	2	17	22	52	25	
LEU3xMIG3	31	16	8	40	19	62	40	
LEU3xRSC3	17	47	23	24	22	128	47	
LEU3xSKN7	33	14	13	40	24	72	52	
LEU3xSTB1	19	28	12	26	7	61	40	
LEU3xSTP2	16	9	6	14	4	39	14	
LEU3xSUT1	39	28	18	44	18	75	41	
LEU3xSUT2	6	6	5	8	8	4	6	
LEU3xSWI4	31	25	13	18	7	64	33	
LEU3xTYE7	10	31	10	28	7	54	28	
LEU3xUME6	14	0	3	19	9	19	13	
LEU3xYJL103C	56	43	17	85	39	116	66	
LYS14xMCM1	82	48	65	81	131	116	66	
LYS14xMIG1	296	369	388	278	299	415	329	
LYS14xNHP10	73	55	50	51	69	91	85	
LYS14xNRG1	208	240	284	169	245	275	301	
LYS14xPDR3	62	42	42	54	58	75	70	
LYS14xPDR8	211	233	260	191	323	381	287	
LYS14xSKN7	214	351	341	144	297	311	274	
LYS14xSTB4	242	164	134	134	138	229	162	
LYS14xSTB5	328	326	356	144	262	266	277	
LYS14xSTE12	312	366	365	149	230	268	287	
LYS14xSUT1	219	379	386	154	272	281	298	
LYS14xSUT2	80	45	59	63	72	77	70	
LYS14xYJL103C	575	443	390	489	485	686	533	
LYS14xYLL054C	21	26	8	11	24	44	38	
LYS14xYRM1	215	343	370	206	311	412	351	
MATA1xRLM1	242	47	131	119	185	209	133	
MATALPHA2xMCM1		408	154	210	506	398	382	287

MATALPHA2xNHP6A	889	1126	995	939	936	997	1064
MATALPHA2xNHP6B	848	1105	1136	986	909	982	1287
MATALPHA2xSPT15	952	1285	1033	956	844	1067	997
MATALPHA2xSTE12	988	1281	1104	976	1041	1042	1119
MATALPHA2xTYE7	826	1175	537	977	714	909	730
MBP1xMCM1	161	75	84	124	211	151	106
MBP1xMET28	175	366	290	162	224	214	314
MBP1xNDD1	98	66	80	113	198	155	99
MBP1xNDT80	13	15	12	12	7	14	15
MBP1xPHD1	161	494	442	170	274	263	309
MBP1xRDS1	103	105	107	121	95	232	159
MBP1xRDS2	233	466	436	203	291	231	348
MBP1xREB1	173	175	219	200	217	244	282
MBP1xRSC3	172	609	464	207	275	323	495
MBP1xRTG3	162	383	387	162	199	267	279
MBP1xSTB1	266	594	440	221	402	367	489
MBP1xSTB2	205	330	356	229	302	268	380
MBP1xSTB3	159	174	161	164	206	167	157
MBP1xSTP1	185	398	331	185	250	309	340
MBP1xSTP2	56	60	52	78	38	130	109
MBP1xSUM1	77	61	61	102	115	89	71
MBP1xSUT1	221	475	526	171	276	347	402
MBP1xSWI4	340	573	461	238	379	407	442
MBP1xSWI6	327	545	576	331	500	416	459
MBP1xTYE7	209	483	161	182	223	322	264
MBP1xUME6	90	39	69	123	84	153	129
MBP1xYDR026C	144	186	119	167	152	223	152
MCM1xMSN2	129	73	81	125	209	111	78
MCM1xNDD1	98	7	22	69	156	93	28
MCM1xNHP10	29	10	22	36	34	29	15
MCM1xPDR1	86	46	68	53	134	71	68
MCM1xPHD1	102	46	99	96	129	118	77
MCM1xRDS2	110	62	67	94	112	73	87
MCM1xRSC3	98	60	77	71	151	113	82
MCM1xSIP4	4	0	0	3	3	10	4

MCM1xSTB1	143	71	93	102	174	99	88	
MCM1xSTE12	90	63	96	78	128	97	102	
MCM1xSTP1	91	50	57	80	129	99	65	
MCM1xSTP2	28	1	5	40	29	24	20	
MCM1xSUT1	104	57	88	87	192	109	63	
MCM1xSWI4	171	70	100	109	166	108	76	
MCM1xSWI6	148	61	99	107	195	114	86	
MCM1xUGA3	66	18	18	40	39	70	15	
MCM1xUME6	29	6	6	26	47	35	21	
MCM1xXBP1	154	64	91	132	231	134	93	
MCM1xYLL054C		5	0	3	9	15	34	5
MCM1xYOX1	33	10	19	24	35	14	8	
MCM1xYPR196W		85	45	56	75	142	79	66
MET28xPHO4	152	265	123	141	116	240	167	
MET28xRSC3	179	396	334	148	281	247	345	
MET28xSTB1	197	360	262	144	205	230	344	
MET28xSTB5	250	290	312	142	219	202	317	
MET28xSUM1	67	74	75	68	98	105	93	
MET28xSUT1	176	386	330	169	257	299	304	
MET28xSWI4	256	356	253	170	186	256	313	
MET28xSWI6	184	322	288	183	214	244	315	
MET28xTYE7	186	386	225	134	165	262	216	
MET28xYNR063W		4	0	2	6	6	4	4
MET31xMIG1	254	459	336	274	341	468	351	
MET31xMIG3	219	262	225	165	326	320	404	
MET31xMSN4	372	357	332	350	344	479	438	
MET31xPDR1	182	394	370	155	260	279	378	
MET31xPHO4	173	313	121	160	126	261	192	
MET31xRDS1	125	111	105	132	113	260	203	
MET31xRPN4	122	148	84	136	118	159	138	
MET31xRSC3	209	424	310	171	272	343	394	
MET31xSKN7	222	415	330	191	314	318	363	
MET31xSTP3	271	369	380	306	286	307	298	
MET31xTYE7	173	387	173	147	204	281	246	
MET32xMET4	45	21	23	61	69	46	34	

MET32xPHO4	211	225	89	188	158	237	178
MET32xTYE7	244	302	152	215	262	283	212
MET4xRDS1	28	19	12	28	21	57	12
MET4xRSC3	35	50	27	8	35	59	27
MET4xSKN7	37	41	35	34	86	67	26
MET4xSTB2	16	15	31	19	27	40	27
MET4xTYE7	59	43	31	55	45	65	20
MIG1xMIG3	385	344	397	350	442	498	440
MIG1xMSN2	349	675	580	434	472	487	496
MIG1xMSN4	538	588	547	662	472	800	496
MIG1xNHP10	93	98	63	103	103	164	104
MIG1xNRG1	327	413	313	239	332	424	346
MIG1xOAF1	192	194	125	191	144	213	146
MIG1xOPI1	59	83	49	82	65	68	47
MIG1xPDR1	332	507	401	276	344	461	371
MIG1xPDR3	94	32	55	80	83	98	72
MIG1xPDR8	268	366	305	283	319	464	351
MIG1xPHD1	281	545	440	312	360	494	399
MIG1xPHO4	218	335	183	258	150	462	219
MIG1xRCS1	334	563	414	316	355	531	337
MIG1xRDS1	182	182	147	264	167	406	161
MIG1xRDS2	431	622	422	276	344	426	384
MIG1xREB1	198	207	258	220	266	374	256
MIG1xRGM1	942	820	743	1148	820	1390	836
MIG1xRGT1	325	533	502	314	335	432	383
MIG1xRPH1	231	238	199	294	200	527	242
MIG1xRSC30	45	30	23	39	19	162	24
MIG1xRSC3	252	579	493	290	333	665	437
MIG1xSFP1	229	435	393	206	335	373	299
MIG1xSIP4	26	9	7	20	11	19	11
MIG1xSKN7	308	557	452	297	415	595	405
MIG1xSOK2	516	535	486	364	288	701	449
MIG1xSTB2	191	413	370	245	264	347	317
MIG1xSTB5	205	485	414	261	323	366	281
MIG1xSTP1	277	559	367	327	288	524	381

MIG1xSTP2	101	104	77	122	46	196	122
MIG1xSUT1	356	640	567	326	432	583	413
MIG1xSUT2	103	104	86	113	114	99	71
MIG1xUGA3	139	98	82	152	93	237	91
MIG1xUME6	156	31	71	169	75	249	152
MIG1xYBL054W	301	579	384	270	375	468	379
MIG1xYDR026C	180	180	156	223	207	340	163
MIG1xYER184C	510	535	539	367	464	635	521
MIG1xYJL103C	774	709	520	978	584	1014	629
MIG1xYLL054C	51	47	24	56	24	148	55
MIG1xYPL230W	942	820	743	1148	820	1390	836
MIG1xYRM1	219	473	488	310	328	561	429
MIG1xZMS1	403	792	652	450	506	664	542
MIG2xMOT3	17	10	45	21	14	7	45
MIG2xOAF1	11	2	6	15	4	8	11
MIG2xRGM1	49	10	15	69	23	54	44
MIG2xSOK2	33	9	29	26	4	27	21
MIG2xSTP2	2	5	0	9	1	7	9
MIG2xSUT1	13	7	18	32	14	10	22
MIG2xSUT2	5	1	2	6	6	7	2
MIG2xYAP7	3	3	3	8	5	12	6
MIG2xYJL103C	25	11	9	30	33	37	22
MIG2xYPL230W	49	10	15	69	23	54	44
MIG3xMSN2	329	399	379	337	436	380	552
MIG3xMSN4	506	342	362	517	436	622	552
MIG3xNRG1	259	221	195	177	299	282	367
MIG3xOAF1	139	118	86	134	152	157	148
MIG3xOPI1	50	46	36	52	48	37	66
MIG3xPDR1	253	280	267	218	338	326	368
MIG3xPDR8	215	209	189	227	329	355	347
MIG3xPHD1	213	292	273	215	357	339	423
MIG3xRDS1	152	123	102	179	145	283	199
MIG3xRDS2	344	368	278	172	348	249	363
MIG3xREB1	171	139	152	164	236	207	240
MIG3xRGM1	831	484	486	859	766	1030	909

MIG3xRGT1	290	304	322	253	316	300	381	
MIG3xRPH1	220	134	140	218	175	392	254	
MIG3xRPN4	108	96	99	151	115	184	145	
MIG3xRSC3	209	348	339	212	344	403	427	
MIG3xSKN7	259	329	270	198	372	423	404	
MIG3xSOK2	430	282	311	252	265	526	465	
MIG3xSTB2	157	249	216	148	260	201	273	
MIG3xSTB5	159	268	296	152	340	227	284	
MIG3xSTP1	249	321	238	234	271	390	391	
MIG3xSTP2	90	68	48	102	41	132	128	
MIG3xSTP3	273	276	255	281	300	334	259	
MIG3xSUT1	349	361	380	233	413	417	432	
MIG3xSUT2	76	72	63	69	122	67	63	
MIG3xUGA3	116	47	50	100	104	172	102	
MIG3xUME6	125	22	54	121	69	193	156	
MIG3xYDR026C	128	106	95	134	185	219	163	
MIG3xYDR520C	20	12	15	24	31	34	31	
MIG3xYER130C	71	39	46	62	82	82	65	
MIG3xYER184C	430	293	343	241	396	441	507	
MIG3xYJL103C	637	383	330	670	540	672	622	
MIG3xYLL054C	37	27	17	36	33	112	54	
MIG3xYPL230W	831	484	486	859	766	1030	909	
MIG3xYPR196W		232	283	315	217	315	298	354
MIG3xYRM1	204	269	314	213	316	386	423	
MIG3xZMS1	354	431	438	333	510	478	639	
MOT3xSKN7	237	663	726	238	631	355	790	
MOT3xUSV1	7	8	27	14	16	11	30	
MSN2xMSN4	551	636	660	771	514	668	604	
MSN2xNDD1	122	63	90	152	196	130	71	
MSN2xPHD1	272	602	485	248	391	351	436	
MSN2xPHO4	251	392	188	266	218	281	251	
MSN2xREB1	228	277	288	282	341	264	320	
MSN2xRGM1	937	917	929	1219	906	1146	993	
MSN2xRPH1	224	311	232	284	210	318	315	
MSN2xRSC3	244	713	622	266	448	315	471	

MSN2xSKN7	263	597	515	293	428	373	463	
MSN2xSOK2	460	588	573	246	392	538	537	
MSN2xSUT1	320	672	618	287	483	403	471	
MSN2xYDR026C		184	240	167	233	247	226	201
MSN2xYER184C	383	539	570	299	482	455	494	
MSN2xYJL103C	629	650	513	816	597	735	585	
MSN2xYPL230W		937	917	929	1219	906	1146	993
MSN2xZMS1	359	876	733	426	515	514	683	
MSN4xNDD1	154	63	78	203	196	174	71	
MSN4xNRG1	407	319	360	367	360	426	420	
MSN4xPHD1	387	499	438	362	391	568	436	
MSN4xRDS1	265	128	142	327	183	410	188	
MSN4xREB1	294	222	258	377	341	384	320	
MSN4xRGM1	1367	745	788	1854	906	1773	993	
MSN4xRPH1	349	241	204	425	210	537	315	
MSN4xRSC3	359	554	532	433	448	538	471	
MSN4xSKN7	389	491	471	441	428	607	463	
MSN4xSTP1	395	465	362	405	325	542	420	
MSN4xSUT1	499	566	551	468	483	685	471	
MSN4xUGA3	156	90	89	214	108	276	116	
MSN4xXBP1	393	407	386	465	483	447	339	
MSN4xYDR026C		265	196	153	355	247	348	201
MSN4xYER184C	549	471	528	436	482	689	494	
MSN4xYJL103C	883	588	496	1187	597	1102	585	
MSN4xYLL054C	46	34	21	62	49	117	38	
MSN4xYPL230W		1367	745	788	1854	906	1773	993
MSN4xZMS1	521	721	651	673	515	834	683	
NDD1xRDR1	99	30	42	91	67	102	72	
NDD1xRDS2	102	66	69	89	137	70	77	
NDD1xRSC30	18	6	6	9	6	23	10	
NDD1xRSC3	102	58	81	80	182	118	109	
NDD1xSTB1	135	61	86	100	177	117	103	
NDD1xSTP1	75	67	65	59	116	99	90	
NDD1xSTP2	23	8	14	30	29	35	23	
NDD1xSUT1	106	45	87	92	152	134	77	

NDD1xSWI4	135	59	86	97	166	113	90	
NDD1xSWI6	106	61	90	92	172	117	86	
NDD1xXBP1	126	58	85	128	241	122	91	
NDD1xYLL054C	5	1	9	4	13	31	8	
NDT80xSKN7	9	10	14	13	4	8	6	
NHP10xNRG1	72	79	29	61	74	104	108	
NHP10xOAF1	34	22	10	25	38	60	39	
NHP10xPDR1	77	79	56	54	65	121	89	
NHP10xPDR8	75	57	40	58	86	125	111	
NHP10xPHD1	52	82	55	51	82	128	114	
NHP10xRDS1	37	45	18	48	25	131	40	
NHP10xRDS2	113	97	47	69	98	99	90	
NHP10xRGM1	238	153	86	248	153	311	225	
NHP10xRSC30	12	9	0	10	3	40	7	
NHP10xRSC3	91	98	60	57	72	163	130	
NHP10xSKN7	71	80	55	71	85	157	101	
NHP10xSTB5	64	79	55	58	88	80	81	
NHP10xSTP1	69	71	46	52	71	142	82	
NHP10xSTP2	27	12	12	22	9	52	28	
NHP10xSUT1	81	101	78	71	87	148	90	
NHP10xYBL054W		73	94	58	49	66	121	85
NHP10xYER184C		105	80	56	81	85	142	130
NHP10xYJL103C	157	115	47	213	107	218	154	
NHP10xYPL230W		238	153	86	248	153	311	225
NHP10xYPR196W		56	73	54	48	80	92	82
NHP10xYRM1	58	81	52	67	88	145	118	
NHP10xZMS1	101	135	84	87	97	168	134	
NHP6AxNHP6B	398	835	438	179	222	178	430	
NHP6AxPHO2	565	658	406	155	276	378	372	
NHP6AxRLM1	174	120	202	148	204	225	159	
NHP6AxSPT15	255	816	350	188	224	204	297	
NHP6AxSTB5	243	341	350	156	241	230	308	
NHP6BxPHO2	697	764	394	134	240	363	415	
NHP6BxRLM1	205	126	240	174	190	181	297	
NHP6BxSTB5	222	318	382	168	237	195	365	

NRG1xOPI1	45	73	44	83	59	40	51	
NRG1xPDR1	224	339	377	135	292	230	302	
NRG1xPHD1	216	342	354	172	323	326	371	
NRG1xRDR1	206	164	210	160	230	245	287	
NRG1xRDS1	141	107	126	152	147	271	187	
NRG1xREB1	199	176	187	145	268	236	286	
NRG1xRGM1	722	469	509	667	723	790	717	
NRG1xRIM101	137	72	87	104	100	129	102	
NRG1xRSC3	258	348	349	184	345	312	414	
NRG1xSKN7	199	387	335	173	422	302	340	
NRG1xSTB2	203	259	265	147	306	222	299	
NRG1xSUT1	271	431	355	167	342	278	378	
NRG1xTBF1	201	270	249	181	211	254	253	
NRG1xYBL054W		256	318	349	172	422	341	395
NRG1xYDR026C	169	130	108	137	195	202	182	
NRG1xYER184C	340	382	445	218	418	354	433	
NRG1xYJL103C	568	454	381	572	504	586	539	
NRG1xYPL230W		722	469	509	667	723	790	717
NRG1xZMS1	319	382	341	227	398	356	460	
OAF1xOPI1	34	20	12	40	39	37	25	
OAF1xPDR1	124	142	118	101	113	112	143	
OAF1xPDR3	58	5	18	29	36	26	30	
OAF1xPDR8	152	96	77	146	132	186	127	
OAF1xRDS1	61	29	48	80	40	105	65	
OAF1xRGT1	139	129	130	140	136	164	115	
OAF1xSTB5	78	127	114	125	147	131	113	
OAF1xSTP1	124	132	92	110	103	105	120	
OAF1xSUT1	156	132	136	105	124	157	144	
OAF1xTYE7	93	164	51	94	97	90	102	
OAF1xUME6	59	15	29	57	26	44	44	
OAF1xYJL103C	311	180	145	360	181	316	176	
OAF1xYPL230W	337	200	164	439	308	379	256	
OAF1xYPR196W		156	131	131	129	127	167	114
OAF1xZMS1	139	217	141	148	200	160	186	
OPI1xRDS2	59	105	56	57	65	60	60	

OPI1xREB1	42	34	34	55	51	55	49
OPI1xRGM1	124	115	74	193	144	149	127
OPI1xRSC30	9	0	3	13	11	20	5
OPI1xRSC3	66	61	64	98	109	79	46
OPI1xSFP1	44	82	50	52	62	58	55
OPI1xSTB2	38	47	46	45	64	51	50
OPI1xSTP3	70	99	53	110	110	89	60
OPI1xSUT1	51	102	60	49	83	41	46
OPI1xSUT2	17	19	7	26	24	27	17
OPI1xYBL054W	87	84	64	55	86	78	68
OPI1xYDR026C	44	31	28	48	42	51	46
OPI1xYER184C	96	85	69	86	113	120	98
OPI1xYJL103C	165	110	78	222	124	184	134
OPI1xYPL230W	124	115	74	193	144	149	127
OPI1xYPR196W	33	69	48	43	57	56	54
OPI1xZMS1	51	116	46	72	61	57	84
PBF1xPBF2	70	73	33	60	94	106	58
PBF1xSTB3	144	76	80	146	134	173	111
PBF2xRTG1	2	16	4	7	3	11	5
PBF2xSTB3	283	269	253	255	411	491	311
PBF2xXBP1	304	353	210	178	296	329	279
PBF2xYBL054W	217	398	219	163	401	334	322
PDR1xPDR3	109	35	71	77	75	81	77
PDR1xRDR1	233	228	255	252	199	217	250
PDR1xRDS1	116	177	159	176	357	258	170
PDR1xRDS2	301	508	368	188	308	244	360
PDR1xREB1	173	228	241	156	258	242	245
PDR1xRGM1	677	703	605	647	691	903	658
PDR1xRGT1	201	425	380	210	327	216	294
PDR1xRSC30	31	29	25	26	36	75	22
PDR1xRSC3	243	578	446	172	326	364	382
PDR1xRTG3	197	378	408	186	214	257	291
PDR1xSKN7	233	553	467	195	401	356	329
PDR1xSTB1	184	401	381	170	237	233	366
PDR1xSTB2	168	331	324	157	263	255	295

PDR1xSTB4	141	187	157	125	191	183	186
PDR1xSTP1	239	540	337	186	258	401	308
PDR1xSUT1	297	606	474	235	329	432	362
PDR1xSWI4	239	372	387	138	204	265	318
PDR1xUGA3	94	88	83	89	81	148	90
PDR1xUME6	115	43	63	132	73	181	129
PDR1xYDR026C	142	194	124	144	155	208	146
PDR1xYER184C	303	473	472	217	388	398	415
PDR1xYJL103C	499	564	423	576	492	615	520
PDR1xYLL054C	26	46	29	27	43	110	25
PDR1xYPL230W	677	703	605	647	691	903	658
PDR1xYRM1	235	430	399	198	279	297	334
PDR1xZMS1	284	605	449	290	407	458	455
PDR3xPDR8	85	19	37	62	62	84	93
PDR3xPHO4	60	18	19	65	36	87	44
PDR3xRDR1	92	18	40	80	61	62	62
PDR3xRDS1	60	16	28	57	36	88	39
PDR3xRGT1	85	24	48	60	72	48	64
PDR3xRSC3	60	49	50	67	97	102	108
PDR3xRTG3	47	22	48	48	51	60	50
PDR3xSKN7	66	28	40	56	96	90	68
PDR3xSKO1	71	24	35	52	48	80	66
PDR3xSTB5	48	22	49	48	69	58	56
PDR3xSTP1	69	38	53	68	79	97	65
PDR3xSUT1	115	40	64	87	88	124	89
PDR3xSUT2	23	2	2	27	18	11	13
PDR3xTYE7	91	32	30	72	43	92	43
PDR3xUME6	44	3	7	35	21	26	17
PDR3xYER184C	79	38	59	44	75	103	89
PDR3xYJL103C	134	41	48	144	94	154	107
PDR3xYRM1	71	26	48	64	68	82	71
PDR8xPHO4	132	212	107	146	146	251	182
PDR8xRDR1	208	133	184	202	199	290	286
PDR8xRDS1	121	85	110	149	131	270	176
PDR8xREB1	159	129	163	161	254	284	256

PDR8xRGT1	242	333	302	256	302	323	325	
PDR8xRSC30	35	12	24	19	27	64	25	
PDR8xRSC3	221	315	296	199	309	372	358	
PDR8xSTB1	179	317	258	154	237	305	333	
PDR8xSTB5	187	294	264	188	278	321	318	
PDR8xSTP1	198	287	212	210	243	393	292	
PDR8xSUT1	251	336	308	216	267	387	365	
PDR8xSUT2	89	44	39	80	85	80	68	
PDR8xTYE7	168	342	160	150	191	271	216	
PDR8xUME6	98	28	39	90	76	135	102	
PDR8xYER184C	308	278	348	209	367	474	424	
PDR8xYJL103C	516	382	311	577	479	796	511	
PDR8xYPR196W		252	312	297	256	290	336	328
PDR8xYRM1	206	295	289	196	307	468	378	
PHD1xPHO4	210	399	175	170	200	318	244	
PHD1xRDS1	166	149	127	176	184	331	204	
PHD1xREB1	176	168	265	153	235	235	232	
PHD1xRGM1	646	745	623	636	695	1003	740	
PHD1xRPH1	170	257	169	162	172	382	258	
PHD1xRSC30	20	45	12	17	35	107	27	
PHD1xRSC3	186	773	448	174	386	532	436	
PHD1xSKN7	210	523	430	183	402	428	360	
PHD1xSOK2	435	503	459	199	286	569	516	
PHD1xSTB2	168	344	360	149	262	214	291	
PHD1xSTP1	232	504	366	201	301	409	398	
PHD1xSTP2	90	91	78	86	53	161	136	
PHD1xSUT1	265	633	437	227	392	415	397	
PHD1xTYE7	201	468	220	147	277	295	256	
PHD1xUGA3	89	86	72	104	89	197	96	
PHD1xXBP1	207	427	360	200	382	294	315	
PHD1xYDR026C	140	160	125	141	185	214	139	
PHD1xYER184C	245	509	456	209	385	411	440	
PHD1xYJL103C	431	623	438	569	449	657	545	
PHD1xYPL230W	646	745	623	636	695	1003	740	
PHD1xZMS1	262	668	497	273	451	451	506	

PHO2xSPT15	351	709	398	166	203	375	372
PHO4xRAP1	137	63	32	75	55	90	68
PHO4xRDS1	134	92	54	180	125	264	118
PHO4xREB1	185	160	148	208	155	262	202
PHO4xRSC3	181	418	222	191	231	441	280
PHO4xSFP1	202	284	118	168	124	216	187
PHO4xSKN7	175	279	199	161	244	343	260
PHO4xSTB2	184	255	174	205	156	229	220
PHO4xSTB4	135	146	58	144	84	147	99
PHO4xSTP1	192	315	133	169	190	305	251
PHO4xSTP2	46	55	34	77	36	105	77
PHO4xSUT1	192	335	208	175	205	363	245
PHO4xTBF1	162	229	144	154	126	206	163
PHO4xTYE7	260	470	167	276	162	449	219
PHO4xYDR026C	160	139	84	172	118	261	127
PHO4xYLL054C	21	19	5	37	47	89	27
PHO4xYRM1	146	260	152	208	135	320	224
RAP1xRCS1	129	144	92	95	118	130	95
RAP1xRPH1	134	44	35	86	83	153	67
RAP1xRSC3	145	204	74	110	120	135	116
RAP1xSFP1	218	139	108	127	147	119	111
RAP1xSTP1	86	96	56	65	123	137	93
RAP1xSTP2	31	22	18	30	20	44	15
RAP1xTYE7	107	96	38	65	63	104	76
RAP1xYJL103C	289	162	92	291	180	249	147
RAP1xYLL054C	10	20	4	10	11	52	11
RCS1xRPH1	227	270	174	193	155	308	236
RCS1xRSC3	252	647	392	191	322	398	368
RCS1xSFP1	213	486	395	164	257	262	287
RCS1xSOK2	441	600	459	209	257	594	478
RCS1xSTP2	84	95	53	92	36	120	99
RCS1xSUT1	257	571	447	208	350	401	346
RCS1xZMS1	291	713	484	259	407	438	448
RDR1xRGT1	188	226	216	265	200	276	306
RDR1xRSC30	39	6	10	36	25	54	28

RDR1xRSC3	249	200	227	234	234	295	352
RDR1xSKN7	230	235	214	181	225	303	318
RDR1xSTB1	199	253	233	186	182	233	315
RDR1xSTP1	198	227	189	210	194	293	300
RDR1xSUT1	252	257	238	241	185	325	320
RDR1xUGA3	97	42	39	102	68	144	72
RDR1xYRM1	225	202	243	238	185	348	335
RDS1xRDS2	181	171	119	154	163	219	213
RDS1xREB1	106	82	60	154	125	193	126
RDS1xRGM1	451	184	209	580	326	753	337
RDS1xRGT1	88	120	109	158	132	203	148
RDS1xRPH1	89	72	54	130	75	287	142
RDS1xRPN4	66	56	48	100	37	111	78
RDS1xRSC30	14	19	15	10	24	92	10
RDS1xRSC3	108	220	154	143	164	365	195
RDS1xSKN7	154	194	151	231	247	311	176
RDS1xSTB1	102	141	115	145	124	233	178
RDS1xSTB2	108	122	88	132	111	207	177
RDS1xSTB5	115	112	134	135	119	185	171
RDS1xSTP1	177	152	97	182	140	370	161
RDS1xSTP2	40	30	24	67	20	133	53
RDS1xSUT1	167	215	194	218	181	382	195
RDS1xSWI4	153	140	120	118	108	271	158
RDS1xSWI6	100	121	113	143	83	221	200
RDS1xUGA3	63	25	30	78	54	170	44
RDS1xUME6	69	8	8	96	53	125	58
RDS1xXBP1	109	115	102	176	195	235	134
RDS1xYDR026C	89	55	37	134	89	193	96
RDS1xYDR520C	12	4	12	12	12	33	5
RDS1xYER184C	176	166	132	146	218	321	211
RDS1xYJL103C	282	172	140	419	245	517	251
RDS1xYLL054C	22	19	9	43	43	111	26
RDS1xYPL230W	451	184	209	580	326	753	337
RDS1xYRM1	137	115	143	169	123	320	184
RDS1xZMS1	167	174	172	250	167	383	251

RDS2xRGM1	831	742	557	699	728	720	752
RDS2xSKN7	339	488	421	206	344	283	378
RDS2xSNT2	25	4	9	16	4	15	6
RDS2xSTB3	231	200	181	175	231	204	207
RDS2xSTB4	222	219	131	153	166	218	196
RDS2xSTB5	234	433	382	140	300	259	287
RDS2xSTP1	302	527	322	201	282	228	353
RDS2xSUT1	375	568	442	186	337	296	371
RDS2xTYE7	207	506	173	156	190	219	256
RDS2xYDR026C	212	197	150	158	163	194	183
RDS2xYJL103C	725	677	422	621	495	627	574
RDS2xYPL230W	831	742	557	699	728	720	752
RDS2xYPR196W	241	482	415	174	330	230	317
RDS2xZMS1	361	701	415	240	427	308	434
REB1xRGM1	528	355	384	651	634	719	597
REB1xRSC30	18	11	6	13	11	52	21
REB1xRSC3	158	188	226	168	199	278	223
REB1xSKN7	158	182	212	166	303	255	279
REB1xSNT2	15	4	5	7	9	25	7
REB1xSTB1	179	207	200	151	207	231	274
REB1xSTB2	167	151	207	158	201	208	262
REB1xSTB3	168	90	131	163	200	218	196
REB1xSTB5	110	198	251	117	249	219	234
REB1xSTP1	200	197	208	164	211	287	287
REB1xSTP2	45	27	39	49	31	104	67
REB1xSUM1	82	26	69	113	89	118	76
REB1xSUT1	181	191	237	153	258	248	233
REB1xSWI4	240	202	199	139	190	243	250
REB1xSWI6	144	174	214	134	188	215	252
REB1xTBF1	194	155	230	174	331	206	240
REB1xTYE7	211	191	211	212	210	297	253
REB1xUME6	95	13	58	106	85	148	108
REB1xYDR026C	131	113	84	111	152	146	123
REB1xYER184C	216	195	285	161	331	357	338
REB1xYJL103C	390	253	285	487	428	588	432

REB1xYPL230W	528	355	384	651	634	719	597	
REB1xYPR196W	140	190	228	164	207	228	206	
REB1xYRM1	169	186	230	177	254	326	268	
REB1xYRR1	16	0	3	9	12	8	17	
REB1xZMS1	211	257	277	236	328	327	343	
RFX1xRSC3	3	0	3	2	0	9	5	
RGM1xRPH1	626	389	292	770	390	969	536	
RGM1xRSC3	717	879	764	740	764	946	747	
RGM1xSKN7	648	756	675	763	817	1081	762	
RGM1xSOK2	1188	729	752	671	670	1479	958	
RGM1xSTP1	660	632	530	758	569	951	694	
RGM1xSUT1	830	824	790	840	818	1182	820	
RGM1xTBF1	538	541	557	610	629	645	623	
RGM1xUGA3	266	124	118	374	187	458	175	
RGM1xUME6	280	61	115	325	178	409	274	
RGM1xYDR026C		469	290	216	561	440	617	376
RGM1xYER130C	214	74	74	242	129	264	154	
RGM1xYER184C	926	688	729	792	857	1233	907	
RGM1xYJL103C	1519	857	680	2136	1090	1963	1070	
RGM1xYLL054C	80	55	39	113	71	232	72	
RGM1xZMS1	916	1050	903	1124	930	1425	1158	
RGT1xRSC3	174	426	439	151	251	272	343	
RGT1xSKN7	188	381	370	189	313	267	345	
RGT1xSTB1	181	407	340	183	250	224	310	
RGT1xSTB3	158	168	198	187	215	191	205	
RGT1xSTP1	193	422	299	192	238	255	304	
RGT1xSUT1	282	473	410	254	318	283	364	
RGT1xSUT2	54	67	54	62	74	64	60	
RGT1xUGA3	105	82	67	104	68	146	66	
RGT1xUME6	78	42	47	95	68	113	85	
RGT1xYJL103C	512	584	427	620	502	647	558	
RGT1xYPR196W		282	445	459	279	341	284	343
RGT1xYRM1	225	450	422	198	317	398	402	
RGT1xZMS1	297	534	498	318	380	343	487	
RLM1xSPT15	185	118	268	177	184	231	183	

RPH1xRSC3	181	313	169	228	174	377	272	
RPH1xSTP3	217	164	178	281	191	324	174	
RPH1xSUT1	228	223	202	224	190	443	270	
RPH1xYER130C	59	21	16	69	36	66	52	
RPH1xYER184C	259	198	195	188	185	427	302	
RPH1xYJL103C	463	229	188	536	242	641	341	
RPH1xYPL230W	626	389	292	770	390	969	536	
RPH1xZMS1	259	329	234	297	228	505	334	
RPN4xSUT1	107	151	116	135	124	165	138	
RPN4xUME6	71	12	27	82	38	106	58	
RPN4xYDR026C	83	78	33	101	92	107	67	
RPN4xZMS1	125	177	150	185	143	198	173	
RSC30xRSC3	32	54	19	31	37	218	41	
RSC30xSKN7	56	36	38	32	43	149	54	
RSC30xSTB2	19	22	13	20	11	41	26	
RSC30xSTP1	41	35	31	26	19	132	38	
RSC30xSTP2	8	9	5	13	2	62	13	
RSC30xSUT1	31	39	26	21	27	134	35	
RSC30xUGA3	18	4	5	12	10	46	5	
RSC30xUME6	28	0	12	23	7	33	8	
RSC30xYER051W		15	4	5	6	12	13	5
RSC30xYER184C		67	29	33	30	32	108	37
RSC30xYJL103C	110	28	32	78	29	145	40	
RSC30xYRM1	50	8	38	31	14	74	43	
RSC30xZMS1	48	44	24	31	45	106	30	
RSC3xRTG3	180	377	483	167	244	257	363	
RSC3xSFP1	221	529	344	167	299	248	360	
RSC3xSKN7	251	602	630	257	507	615	477	
RSC3xSTB1	197	615	464	118	258	273	386	
RSC3xSTB2	179	422	351	197	196	236	293	
RSC3xSTB5	227	419	472	125	326	265	336	
RSC3xSTP1	266	677	454	195	336	522	532	
RSC3xSTP2	79	149	70	71	50	212	163	
RSC3xSUT1	206	771	600	206	370	553	535	
RSC3xSUT2	65	94	74	68	127	52	59	

RSC3xSWI4	271	552	473	141	229	250	343
RSC3xSWI5	405	652	521	334	441	693	505
RSC3xSWI6	171	540	432	150	208	250	352
RSC3xTHI2	2	6	10	0	2	0	11
RSC3xUGA3	95	116	105	101	106	216	111
RSC3xUME6	177	39	112	136	88	201	186
RSC3xXBP1	244	436	350	271	466	277	362
RSC3xYBL054W	245	546	391	167	330	304	385
RSC3xYDR026C	162	203	152	147	187	262	132
RSC3xYER051W	70	86	92	61	84	60	63
RSC3xYER184C	354	607	579	221	416	594	524
RSC3xYJL103C	622	661	528	647	522	868	651
RSC3xYLL054C	11	61	28	28	53	197	41
RSC3xYPL230W	717	879	764	740	764	946	747
RSC3xYPR196W	173	324	371	155	206	231	338
RSC3xYRM1	210	336	442	188	263	386	412
RSC3xZMS1	286	790	553	267	465	428	505
RTG3xSKN7	180	390	433	186	267	309	320
RTG3xSTB1	186	360	405	168	248	258	365
RTG3xSTB5	242	360	380	150	225	214	303
RTG3xSUM1	80	91	98	97	105	113	87
RTG3xSUT1	178	372	396	202	249	284	344
RTG3xSWI4	230	349	399	166	225	284	333
RTG3xSWI6	162	363	372	174	184	239	291
RTG3xTYE7	178	399	248	184	223	308	227
SFP1xTYE7	192	428	167	161	173	251	220
SIG1xXBP1	281	102	112	248	144	92	85
SIP4xSKN7	23	3	6	31	11	12	7
SIP4xSTP1	21	6	18	14	11	14	14
SKN7xSOK2	389	543	488	236	330	600	459
SKN7xSTB1	160	405	488	165	271	303	345
SKN7xSTB4	167	167	149	143	184	236	171
SKN7xSTB5	141	406	381	187	357	264	297
SKN7xSTP1	196	484	347	215	378	417	383
SKN7xSUT1	252	677	528	241	442	495	404

SKN7xSUT2	61	82	54	70	100	60	59
SKN7xSWI4	218	403	482	138	241	315	289
SKN7xSWI5	364	594	536	442	447	637	494
SKN7xSWI6	142	389	444	179	226	269	319
SKN7xUGA3	119	82	94	110	113	223	110
SKN7xUME6	90	31	53	98	89	165	134
SKN7xYBL054W	221	546	426	190	455	376	398
SKN7xYDR026C	137	197	133	161	232	271	189
SKN7xYDR520C	16	28	14	19	38	33	23
SKN7xYER184C	381	467	485	231	496	466	504
SKN7xYJL103C	580	560	444	588	544	758	569
SKN7xYLL054C	55	50	20	35	70	107	41
SKN7xYPL230W	648	756	675	763	817	1081	762
SKN7xYRM1	177	356	362	206	324	407	398
SKN7xZMS1	276	617	465	286	453	552	504
SKO1xSUM1	80	84	99	139	107	149	119
SNF1xYBL054W	1	0	1	2	18	1	2
SNT2xSTB2	17	4	7	7	12	15	4
SNT2xSTB5	35	4	5	10	2	23	9
SNT2xSTP3	17	4	2	22	6	17	5
SNT2xYER184C	8	3	9	6	11	22	12
SOK2xSUT1	402	552	500	230	290	540	474
SOK2xYPL230W	1188	729	752	671	670	1479	958
SOK2xZMS1	472	599	507	286	326	620	582
SPT15xUPC2	313	550	547	292	250	303	418
SPT23xSTB3	162	200	206	146	186	188	279
SRD1xYER130C	14	6	4	19	12	10	13
STB1xSTB2	170	377	271	143	238	241	370
STB1xSTB3	175	161	156	138	211	230	217
STB1xSTP1	186	429	286	167	201	270	346
STB1xSUT1	197	470	474	164	268	276	344
STB1xSWI4	359	624	495	260	401	368	528
STB1xSWI6	267	602	444	216	393	297	488
STB1xUGA3	102	71	69	78	89	174	106
STB1xXBP1	256	349	379	224	431	282	359

STB1xYDR026C	150	191	140	137	144	178	165
STB1xYER184C	247	408	476	150	301	364	443
STB1xYLL054C	16	28	25	21	23	79	24
STB1xYRM1	184	384	366	186	242	354	375
STB2xSTB3	170	131	178	153	221	219	185
STB2xSTP1	189	328	282	158	223	241	343
STB2xSTP2	46	52	54	53	25	89	98
STB2xSUM1	84	72	81	112	89	90	87
STB2xSUT1	148	329	351	137	277	211	341
STB2xSWI4	233	372	281	137	222	277	343
STB2xSWI6	176	327	317	159	278	267	320
STB2xTBF1	197	290	316	170	329	194	279
STB2xTYE7	206	304	234	202	217	269	282
STB2xUME6	85	25	76	130	93	145	125
STB2xYDR026C	136	150	130	116	159	162	152
STB2xYER184C	227	342	415	170	343	363	422
STB2xYJL103C	402	472	385	513	438	594	509
STB2xYRR1	9	3	4	12	11	11	16
STB2xZMS1	202	507	366	225	345	286	364
STB3xSWI4	223	155	158	156	196	271	193
STB3xSWI6	145	171	147	128	212	172	181
STB3xXBP1	208	199	210	213	242	256	237
STB3xYBL054W	260	290	298	164	407	320	358
STB3xYDR026C	132	70	49	110	138	152	110
STB3xYRR1	8	2	4	9	12	12	10
STB4xSTB5	136	174	134	153	138	194	186
STB4xSUT1	151	239	160	166	169	207	199
STB4xSUT2	36	22	15	49	68	43	39
STB4xUGA3	71	30	33	80	38	94	53
STB4xYER184C	180	186	171	160	235	310	216
STB4xYJL103C	330	231	172	436	297	519	265
STB5xSTP2	72	60	56	77	36	90	67
STB5xSUT1	147	422	471	144	329	257	293
STB5xSUT2	45	87	58	67	96	74	65
STB5xYDR520C	16	20	19	26	24	27	19

STB5xYER184C	248	415	475	201	369	349	372
STB5xYJL103C	453	555	447	535	432	610	476
STP1xSTP2	76	108	63	90	61	175	110
STP1xSTP3	283	369	332	292	307	395	309
STP1xSUM1	95	52	54	85	68	97	92
STP1xSUT1	233	601	462	285	325	529	407
STP1xSWI4	245	415	296	156	189	319	296
STP1xSWI6	156	380	286	162	206	266	287
STP1xTHI2	10	3	6	3	1	0	6
STP1xTYE7	180	463	172	146	249	276	258
STP1xUGA3	92	86	55	95	70	218	81
STP1xUME6	110	46	81	137	71	238	145
STP1xYDR026C	160	206	123	176	201	251	179
STP1xYER184C	280	493	417	219	357	465	427
STP1xYJL103C	481	578	354	628	407	698	541
STP1xYLL054C	22	42	17	31	37	167	35
STP1xYPL230W	660	632	530	758	569	951	694
STP1xZMS1	266	638	425	353	342	496	487
STP2xSUM1	28	7	9	29	14	48	26
STP2xSUT1	78	123	79	106	63	175	134
STP2xYER184C	84	94	84	73	53	182	119
STP2xYJL103C	153	107	73	225	49	288	137
STP2xYLL054C	4	15	7	18	6	60	19
STP2xZMS1	94	128	82	136	48	165	150
STP3xSUT1	283	423	447	304	317	381	292
SUM1xSWI6	84	73	62	70	120	81	57
SUT1xSUT2	71	78	68	64	103	75	55
SUT1xSWI4	244	444	495	154	237	302	295
SUT1xSWI6	183	423	460	176	217	237	328
SUT1xTYE7	215	418	231	182	284	320	304
SUT1xUGA3	125	95	107	97	98	215	102
SUT1xUME6	140	30	96	148	83	241	186
SUT1xYDR026C	149	168	164	152	237	248	162
SUT1xYDR520C	31	22	36	16	27	35	38
SUT1xYER130C	70	59	67	63	72	94	61

SUT1xYER184C	444	542	556	237	475	510	507
SUT1xYJL103C	635	623	514	644	540	764	598
SUT1xYLL054C	51	60	37	50	50	171	44
SUT1xYPL230W	830	824	790	840	818	1182	820
SUT1xYPR196W	238	449	427	200	257	297	323
SUT1xYRM1	216	426	441	208	294	409	421
SUT1xZMS1	353	745	645	355	478	577	568
SUT2xXBP1	75	77	69	96	131	46	64
SUT2xYER184C	104	103	69	78	96	86	85
SUT2xYJL103C	175	127	56	208	131	151	117
SUT2xZMS1	78	98	96	111	161	85	84
SWI4xSWI6	353	586	462	257	372	355	449
SWI4xUGA3	126	66	71	63	85	163	93
SWI4xXBP1	339	352	383	245	407	296	332
SWI4xYDR026C	204	182	139	124	132	185	148
SWI4xYLL054C	28	24	28	26	19	75	21
SWI5xYLL054C	62	36	39	71	52	137	65
SWI6xUGA3	99	61	57	62	81	135	71
SWI6xYDR026C	150	182	130	136	133	185	135
TBF1xTYE7	179	328	202	133	193	253	190
TBF1xYDR026C	144	129	105	128	248	155	135
TBF1xYPL230W	538	541	557	610	629	645	623
TBF1xYRR1	17	1	7	9	24	9	15
TEC1xUPC2	369	526	434	323	266	300	508
TEC1xYER130C	61	56	55	52	58	61	39
TYE7xUGA3	39	87	25	67	57	163	59
TYE7xUME6	125	44	68	109	79	182	124
TYE7xYDR026C	172	152	115	154	166	255	171
TYE7xYDR520C	25	40	6	24	7	15	22
TYE7xYER184C	246	387	247	186	304	411	361
TYE7xYJL103C	415	558	192	517	358	609	399
TYE7xYLL054C	21	29	5	43	49	93	27
TYE7xYRM1	188	438	209	215	231	316	269
TYE7xYRR1	11	5	9	7	6	22	15
UGA3xYER184C	144	88	89	124	70	192	129

UGA3xYJL103C	223	123	71	290	104	335	151	
UGA3xYLL054C	16	3	2	9	21	75	15	
UGA3xYPL230W		266	124	118	374	187	458	175
UME6xYDR026C		69	20	30	92	44	110	64
UME6xYER184C	140	41	82	91	102	195	139	
UME6xYLL054C	20	4	9	31	10	58	10	
UME6xYPL230W		280	61	115	325	178	409	274
UME6xYPR196W		73	24	62	79	68	93	98
UME6xZMS1	132	64	99	164	90	233	207	
USV1xZMS1	18	12	24	22	11	26	32	
XBP1xYBL054W	412	516	350	191	416	356	373	
YAP3xYAP7	194	34	87	215	118	117	111	
YBL054WxYLL054C		12	30	21	17	19	123	31
YDR026CxYER130C		43	23	17	48	34	52	24
YDR026CxYER184C		235	199	167	150	289	313	233
YDR026CxYJL103C		384	244	187	437	330	474	270
YDR026CxYLL054C		18	14	10	35	35	50	18
YDR026CxYPL230W		469	290	216	561	440	617	376
YDR026CxZMS1	182	225	170	194	240	273	229	
YDR520CxZMS1	28	29	26	42	34	33	44	
YER130CxYPL230W		214	74	74	242	129	264	154
YER130CxZMS1	94	68	84	110	85	128	99	
YER184CxYJL103C		788	639	544	696	689	974	788
YER184CxYLL054C		59	37	31	21	62	117	47
YER184CxYPL230W		926	688	729	792	857	1233	907
YER184CxYRM1	267	385	465	208	374	524	506	
YER184CxZMS1	441	579	567	317	448	574	629	
YJL103CxYLL054C		77	41	21	80	52	181	59
YJL103CxYPL230W		1519	857	680	2136	1090	1963	1070
YJL103CxYPR196W		493	522	404	522	468	605	504
YJL103CxYRM1	432	514	461	586	471	846	623	
YJL103CxZMS1	683	684	503	828	638	888	766	
YKL222CxYRM1	17	10	10	13	9	23	9	
YLL054CxYPL230W		80	55	39	113	71	232	72
YLL054CxZMS1	42	65	43	56	38	152	66	

YPL230WxZMS1		916	1050	903	1124	930	1425	1158
YPR196WxYRM1		198	404	399	207	318	356	389
YRM1xZMS1	230	498	461	310	396	480	547	
